# High-coverage genomes to elucidate the evolution of penguins

**DOI:** 10.1093/gigascience/giz117

**Published:** 2019-09-18

**Authors:** Hailin Pan, Theresa L Cole, Xupeng Bi, Miaoquan Fang, Chengran Zhou, Zhengtao Yang, Daniel T Ksepka, Tom Hart, Juan L Bouzat, Lisa S Argilla, Mads F Bertelsen, P Dee Boersma, Charles-André Bost, Yves Cherel, Peter Dann, Steven R Fiddaman, Pauline Howard, Kim Labuschagne, Thomas Mattern, Gary Miller, Patricia Parker, Richard A Phillips, Petra Quillfeldt, Peter G Ryan, Helen Taylor, David R Thompson, Melanie J Young, Martin R Ellegaard, M Thomas P Gilbert, Mikkel-Holger S Sinding, George Pacheco, Lara D Shepherd, Alan J D Tennyson, Stefanie Grosser, Emily Kay, Lisa J Nupen, Ursula Ellenberg, David M Houston, Andrew Hart Reeve, Kathryn Johnson, Juan F Masello, Thomas Stracke, Bruce McKinlay, Pablo García Borboroglu, De-Xing Zhang, Guojie Zhang

**Affiliations:** 1 BGI-Shenzhen, Beishan Industrial Zone, Yantian District, Shenzhen 518083, China; 2 State Key Laboratory of Genetic Resources and Evolution, Kunming Institute of Zoology, Chinese Academy of Sciences, Kunming, China; 3 Section for Ecology and Evolution, Department of Biology, University of Copenhagen, DK-2100 Copenhagen, Denmark; 4 Manaaki Whenua Landcare Research, PO Box 69040, Lincoln, Canterbury 7640, New Zealand; 5 Department of Zoology, University of Otago, PO Box 56, Dunedin, Otago 9054, New Zealand; 6 China National Genebank, BGI-Shenzhen, Shenzhen, Guangdong, China; 7 Center for Excellence in Animal Evolution and Genetics, Chinese Academy of Sciences, Kunming 650223, China; 8 Bruce Museum, Greenwich, CT 06830, USA; 9 Department of Zoology, University of Oxford, 11a Mansfield Road, Oxford OX1 3SZ, UK; 10 Department of Biological Sciences, Bowling Green State University, Bowling Green, OH 43403, USA; 11 The Wildlife Hospital Dunedin, School of Veterinary Nursing, Otago Polytechnic, Dunedin, Otago 9016, New Zealand; 12 Copenhagen Zoo, Roskildevej 38, DK-2000 Frederiksberg, Denmark; 13 Department of Veterinary and Animal Sciences, University of Copenhagen, Copenhagen, Denmark; 14 Center for Ecosystem Sentinels, Department of Biology, University of Washington, Seattle, WA 98195, USA; 15 Centre d'Etudes Biologiques de Chizé (CEBC), UMR 7372 du CNRS-La Rochelle Université, 79360 Villiers-en-Bois, France; 16 Research Department, Phillip Island Nature Parks, PO Box 97, Cowes, Phillip Island, Victoria, 3922, Australia; 17 Department of Zoology, University of Oxford, Peter Medawar Building for Pathogen Research, South Parks Road, Oxford OX1 3SY, UK; 18 Hornby Veterinary Centre, 7 Tower Street, Hornby, Christchurch, Canterbury 8042, New Zealand; 19 South Island Wildlife Hospital, Christchurch, Canterbury, New Zealand; 20 National Zoological Garden, South African National Biodiversity Institute, P.O. Box 754, Pretoria 0001, South Africa; 21 Division of Pathology and Laboratory Medicine, University of Western Australia, Crawley, Western Australia 6009, Australia; 22 Institute for Marine and Antarctic Studies, University of Tasmania, Hobart, Tasmania 7001, Australia; 23 Department of Biology, University of Missouri St. Louis, St Louis, MO 63121, USA; 24 British Antarctic Survey, Natural Environment Research Council, High Cross, Cambridge, UK; 25 Justus-Liebig-Universität Giessen, Heinrich-Buff-Ring 26, 35392 Giessen, Germany; 26 FitzPatrick Institute of African Ornithology, University of Cape Town, Rondebosch 7701, South Africa; 27 Vet Services Hawkes Bay Ltd, 801 Heretaunga Street, Hastings, New Zealand; 28 Wairoa Farm Vets, 77 Queen Street, Wairoa 4108, New Zealand; 29 National Institute of Water and Atmospheric Research Ltd., Private Bag 14901, Kilbirnie, Wellington 6241, New Zealand; 30 Section for Evolutionary Genomics, The GLOBE Institute, Faculty of Health and Medical Sciences, University of Copenhagen, Øster Farimagsgade 5A, Copenhagen, Denmark; 31 NTNU University Museum, Trondheim, Norway; 32 Museum of New Zealand Te Papa Tongarewa, PO Box 467, Wellington 6140, New Zealand; 33 Division of Evolutionary Biology, Faculty of Biology, LMU Munich, Großhaderner Str. 2, 82152 Planegg-Martinsried, Germany; 34 Wildbase, Massey University, Private Bag 11 222, Palmerston North 4442, New Zealand; 35 Wellington Zoo, 200 Daniell St, Newtown, Wellington 6021, New Zealand; 36 National Zoological Gardens of South Africa, Pretoria, South Africa; 37 Department of Ecology, Environment and Evolution, La Trobe University, Melbourne, Victoria, Australia; 38 Global Penguin Society, University of Washington, Seattle, WA, USA; 39 Biodiversity Group, Department of Conservation, Auckland, New Zealand; 40 Department of Biology, Natural History Museum of Denmark, University of Copenhagen, Copenhagen, Denmark; 41 Biodiversity Group, Department of Conservation, Dunedin, New Zealand; 42 Global Penguin Society, Puerto Madryn 9120, Argentina; 43 CESIMAR CCT Cenpat-CONICET, Puerto Madryn 9120, Chubut, Argentina; 44 Center for Computational and Evolutionary Biology, Institute of Zoology, Chinese Academy of Sciences, 1 Beichen West Road, Beijing 100101, China

**Keywords:** genomics, Sphenisciformes, comparative evolution, phylogenetics, speciation, biogeography, demography, climate change, Antarctica, evolution

## Abstract

**Background:**

Penguins (Sphenisciformes) are a remarkable order of flightless wing-propelled diving seabirds distributed widely across the southern hemisphere. They share a volant common ancestor with Procellariiformes close to the Cretaceous-Paleogene boundary (66 million years ago) and subsequently lost the ability to fly but enhanced their diving capabilities. With ∼20 species among 6 genera, penguins range from the tropical Galápagos Islands to the oceanic temperate forests of New Zealand, the rocky coastlines of the sub-Antarctic islands, and the sea ice around Antarctica. To inhabit such diverse and extreme environments, penguins evolved many physiological and morphological adaptations. However, they are also highly sensitive to climate change. Therefore, penguins provide an exciting target system for understanding the evolutionary processes of speciation, adaptation, and demography. Genomic data are an emerging resource for addressing questions about such processes.

**Results:**

Here we present a novel dataset of 19 high-coverage genomes that, together with 2 previously published genomes, encompass all extant penguin species. We also present a well-supported phylogeny to clarify the relationships among penguins. In contrast to recent studies, our results demonstrate that the genus *Aptenodytes* is basal and sister to all other extant penguin genera, providing intriguing new insights into the adaptation of penguins to Antarctica. As such, our dataset provides a novel resource for understanding the evolutionary history of penguins as a clade, as well as the fine-scale relationships of individual penguin lineages. Against this background, we introduce a major consortium of international scientists dedicated to studying these genomes. Moreover, we highlight emerging issues regarding ensuring legal and respectful indigenous consultation, particularly for genomic data originating from New Zealand Taonga species.

**Conclusions:**

We believe that our dataset and project will be important for understanding evolution, increasing cultural heritage and guiding the conservation of this iconic southern hemisphere species assemblage.

## Data Description

### Context

**Figure 1:**
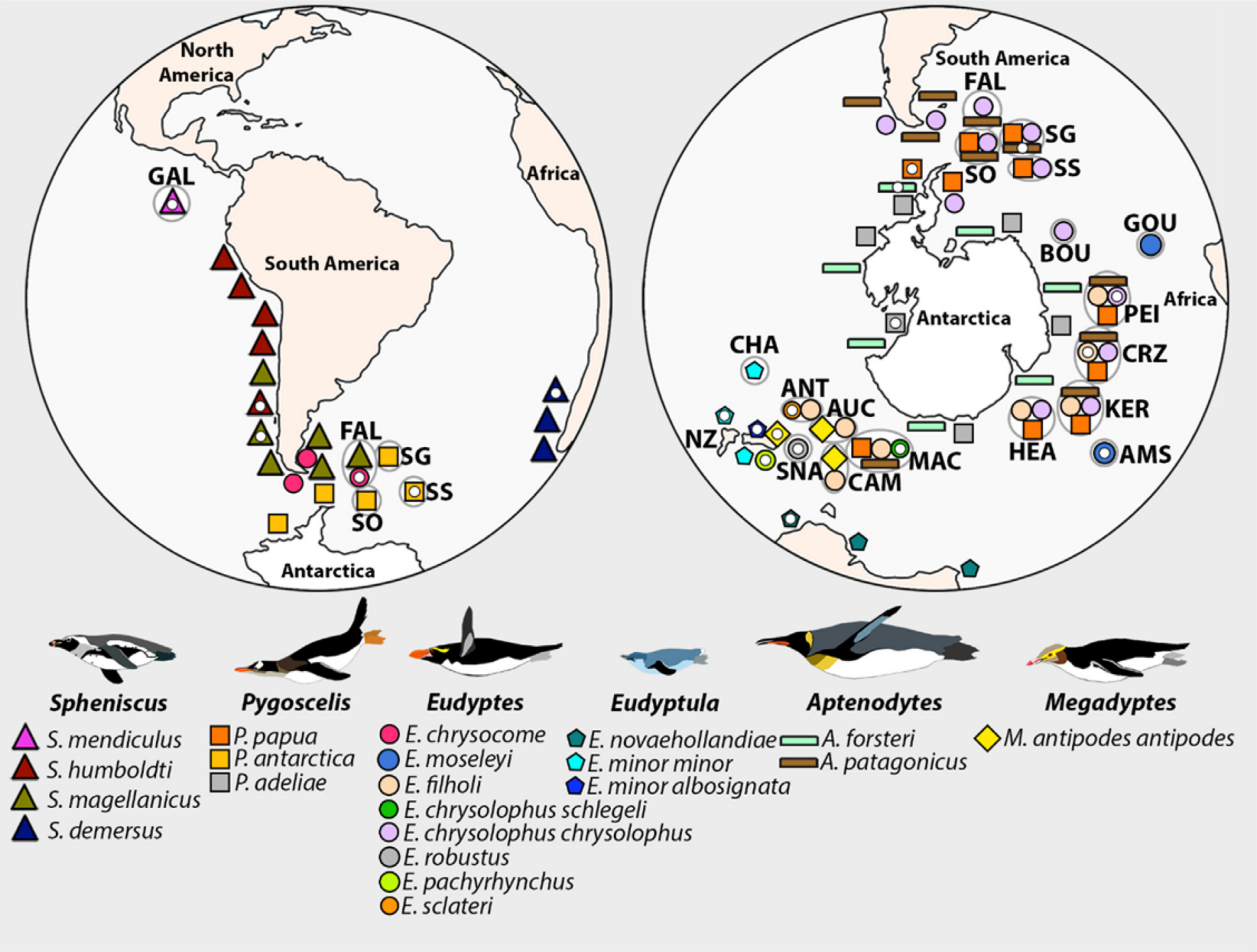
Locations of breeding colonies of penguins and sampling sites for the final genomes, adapted from Ksepka et al. [1]. Sampling locations are shown with a small white ellipse. Note that the sampling location of the humboldt penguin (*Spheniscus humboldti*) is unclear because this individual was bred in the Copenhagen zoo, with ancestors imported from Peru and Chile in 1972. AMS: Amsterdam Island; ANT: Antipodes Islands; AUC: Auckland Islands; BOU: Bouvet; CAM: Campbell Island; CHA: Chatham Islands; CRZ: Crozet; FAL: Falkland Islands/Malvinas; GAL: Galapagos Islands; GOU: Gough Island; HEA: Heard Island; KER: Kerguelen; MAC: Macquarie Island; NZ: New Zealand; PEI: Prince Edward/Marion Island; SG: South Georgia; SNA: The Snares; SO: South Orkney Islands; SS: South Sandwich Islands.

Penguins (Sphenisciformes) are a unique order of seabirds distributed widely across the southern hemisphere (Fig. 1). Approximately 20 extant penguin species are recognized across 6 well-defined genera (*Aptenodytes, Pygoscelis, Eudyptula, Spheniscus, Eudyptes*, and *Megadyptes* [1–3]). Debate has surrounded species/lineage boundaries in a few key areas:

1. Divisions between New Zealand little blue (*Eudyptula minor minor*), New Zealand white-flippered (*Eudyptula minor albosignata*), and Australian fairy penguins (*Eudyptula novaehollandiae*) [4–6].
2. Divisions between northern rockhopper (*Eudyptes moseleyi*), western rockhopper (*Eudytes chrysocome*), and eastern rockhopper penguins (*Eudyptes filholi*) [3, 7, 8].
3. Divisions between Fiordland crested (*Eudyptes pachyrhynchus*) and Snares crested penguins (*Eudyptes robustus*) [9, 10].
4. Divisions between macaroni (*Eudyptes chrysolophus chrysolophus*) and royal penguins (*Eudyptes chrysolophus schlegeli*) [3, 8, 11].

Penguins have an extensive fossil record, with >50 extinct species documented to date [3, 12, 13], extending back >60 million years [12]. Extant penguins span a modest range of sizes [14, 15], with the emperor penguin (*Aptenodytes forsteri*) the largest (30 kg) and *Eudyptula* penguins the smallest (1 kg). In contrast, the fossil record reveals that many extinct penguin species were giants (surpassing 100 kg in body mass [13]).

The radiation of penguins provides an excellent case study for researching biogeographic impacts on speciation processes. Penguins inhabit every major coastline in the southern hemisphere, and almost every island archipelago in the Southern Ocean [16]. Their range extends to unique ecological niches, from the tropical Galápagos Islands (Galápagos penguin, *Spheniscus mendiculus*) to the oceanic temperate forests of New Zealand (*Eudyptes pachyrhynchus*), rocky coastlines of the sub-Antarctic islands (*E. filholi*), and the sea ice around Antarctica (*Aptenodytes forsteri*) [17]. For this reason, penguins have evolved many unique adaptations, specific to the variety of ecological environments. Previous studies have suggested that global climate change during the Eocene [18, 19], substantial oceanographic currents [7], and geological island uplift [3] were key drivers of penguin diversification. Although the phylogenetic relationships within penguins are relatively well understood [1, 3, 18, 20], it remains uncertain which lineage first diverged from other penguins. Molecular analyses have differed on whether *Aptenodytes, Pygoscelis*, or both together represent the sister taxa to all other extant penguins [3]. Both of these genera are endemic to coastal Antarctica and Antarctic and subantarctic islands, and thus a sequential branching pattern would suggest a polar ancestral area for extant penguins. In contrast, morphological data and the fossil record suggest that the more temperate-adapted genus *Spheniscus* was the first to diverge [3, 20]. Understanding the evolutionary diversification of penguins in respect to geological and climatic changes remains a substantial gap in understanding the biogeographic history of these iconic birds.

Although penguins are tied to landmasses for breeding and nesting [21], all species spend most of their lives at sea [22] and are therefore important components of terrestrial, coastal, and marine ecosystems [23]. While some taxa inhabit environments with strong winds and extreme cold temperatures, experiencing seasonal fluctuations in the length of daylight across the breeding and chick-rearing seasons [24], others inhabit relatively temperate or even tropical climates, with little variation in day length. The unique morphological and physiological adaptations that have evolved within penguins include the complete loss of aerial flight, where penguins instead use their flipper-like wings in wing-propelled diving [25], densely packed waterproof and insulating feathers [26, 27], visual sensitivity of the eye lens for underwater predation [28–30], dense bones, stiff wing joints and reduced distal wing musculature to overcome buoyancy in water [31–33], enhanced thermoregulation for extreme low temperatures, long-term fasting, ability to digest secreted food, delayed digestion [34–40], different plumage [41] and crest ornaments [42], and catastrophic moult [43]. As such, penguins are an excellent system to study comparative evolution of adaptive traits.

Penguins are also sentinels of the Southern Ocean [16], being particularly sensitive to human and environmental change [44, 45]. Extensive demographic monitoring programs have indicated that many penguin species are declining in response to global warming [44–46], pollution, environmental degradation, and competition with fisheries, which are considered key drivers of these population declines [47–50]. Demographic coalescent models have demonstrated dramatic population declines during the Pleistocene ice ages, followed by rapid population expansions in response to global warming [51–54]. Future global warming is predicted to cause significant population declines [44, 55–57]. Understanding past demographic histories and inferring future demographic trajectories therefore remain important steps for predicting ecosystem-wide changes in this rapidly warming part of the planet.

Although penguins are a relatively well-studied group, previous evolutionary studies have been limited by the genetic markers used, such as short mitochondrial [2, 10, 58–60] or nuclear sequences [1, 8, 61, 62], microsatellites [63, 64], partial mitochondrial genomes [3, 65], or single-nucleotide polymorphisms [11, 53, 54, 66–68]. Several studies have hinted at associations between biological patterns and climate change [51–54, 60, 69]. Only a few studies have explored genome-wide evolutionary processes among penguins [51, 70] or between penguins and other birds [71–73], and these studies have focussed on just 2 Antarctic taxa: the Adélie penguin (*Pygoscelis adeliae*) and *Aptenodytes forsteri*. These previous studies have created a basic framework to understand the timing of penguin diversification, identify population fluctuations during past climate cycles, and have hinted at the molecular basis for a range of physiological and morphological adaptations [51]. The molecular genomic basis for the unique morphological and physiological adaptations of penguins, compared to other aquatic and terrestrial birds, remains largely unknown. No previous study has attempted to explore the evolution of all penguins under a comparative genomic or evolutionary framework. In this Data Note, we present 19 new high-quality genomes that, together with the 2 previously reported genomes [51], encompass all extant penguin species. We demonstrate the quality and application of this new dataset by constructing a well-supported phylogenomic tree of penguins. These data provide a critical resource for understanding the drivers of penguin evolution, the molecular basis of morphological and physiological adaptations, and demographic characteristics. For species naming, we follow standard nomenclature; however, for *Eudyptula* we follow Grosser et al. [5, 74] and for *Eudyptes* and *Megadyptes* we follow Cole et al. [3].

### Methods

#### Sample collection, library construction, and sequencing

While it is possible to recover genome sequences from historical museum samples [75], such genomes are often of low quality and/or fragmented [76], limiting the ability of downstream analyses. Our project design (see below) relies on high-coverage genomes with little missing data (see Li et al. [51]). Therefore, we designed our sample collection to include only high-quality blood samples. We collected 94 blood samples spanning 19 different penguin species (1–28 samples per species; Supplementary Table 1). Samples were derived from the wild, zoological parks, or wildlife hospitals and were obtained according to strict permitting procedures, animal ethics, and consultation with indigenous representatives (Supplementary Table 1).

DNA was extracted from each sample at 1 of 3 laboratories as follows: we used the HiPire Blood DNA Midi Kit II at BGI (Hong Kong), the Qiagen DNeasy Blood and Tissue Kit (Qiagen, Valencia, CA, USA) at the University of Oxford (United Kingdom), and the KingFisher Cell and Tissue Kit in combination with the KingFisher Duo Prime Purification System at the University of Copenhagen (Denmark). All downstream methods were conducted at BGI. We diluted each DNA extraction to 20 μL using Tris-EDTA buffer. The quality and quantity of each DNA extraction was assessed by first estimating the concentration of 1 μL DNA extraction on a Microplate Reader, and DNA fragment size was evaluated by pulse gel electrophoresis or 1% agarose gel electrophoresis. Following quality control, a single sample per species was chosen for genomic library construction (Table 1).

**Table 1:** Sample collection information for the 21 penguin genomes (including 2 obtained in Li et al. (51)

Latin name	Common name	Sample type	Sampling location	Sample label	Date extracted
*Eudyptes chrysolophus schlegeli*	Royal	Wild	Green Gorge, Macquarie Island	4458	October 2017
*Eudyptes chrysolophus chrysolophus*	Macaroni	Wild	Marion Island, Prince Edward Islands	MP PEI 1	October 2017
*Eudyptes pachyrhynchus*	Fiordland-crested	Wild	Harrison Cove, Milford Sound, New Zealand South Island	MS 9	May 2017
*Eudyptes robustus*	Snares-crested	Dunedin Wildlife Hospital	The Snares, New Zealand sub-Antarctic	68M 28/09/13	September 2018
*Eudyptes sclateri*	Erect-crested	Wild	Antipodes Island, New Zealand sub-Antarctic	Ant 5	September 2018
*Eudyptes filholi*	Eastern rockhopper	Wild	Crozet Island	GS 12	May 2016
*Eudyptes chrysocome*	Western rockhopper	Wild	Falkland Islands/Malvinas	RH 110–1	May 2016
*Eudyptes moseleyi*	Northern rockhopper	Wild	Amsterdam Island	NRP 118–1	May 2016
*Megadyptes antipodes antipodes*	Yellow-eyed	Wild	Otago Peninsula, New Zealand South Island	OT 2 9/2/18	August 2018
*Spheniscus magellanicus*	Magellanic	Wild	Chiloe Island, Chile	AH 6	May 2016
*Spheniscus demersus*	African	Wild	Luderitz, Namibia	AP 173	July 2018
*Spheniscus mendiculus*	Galápagos	Wild	Galápagos Islands	GAPE 212	October 2017
*Spheniscus humboldti*	Humboldt	Copenhagen Zoo	Peru and Chile lineage	Z-67–15	October 2016
*Eudyptula minor albosignata*	White-flippered	Christchurch Antarctic Centre	Banks Peninsula, Canterbury, New Zealand South Island	Fred	July 2018
*Eudyptula minor minor*	Little blue	National Aquarium of New Zealand	New Zealand North Island	Gonzo	August 2018
*Eudyptula novaehollandiae*	Fairy	Wild	Phillip Island, Victoria, Australia	10/9/18–1	October 2018
*Pygoscelis adeliae*	Adélie	Wild	Inexpressible Island, Antarctica	[51]	NA
*Pygoscelis papua*	Gentoo	Wild	West Antarctic Peninsula, Antarctica	Gentoo penguin DNA -4	January 2018
*Pygoscelis antarctica*	Chinstrap	Wild	Thule Island, South Sandwich Islands	CP TH 060	November 2017
*Aptenodytes patagonicus*	King	Wild	Fortuna Bay, South Georgia	KP FORT 001	November 2017
*Aptenodytes forsteri*	Emperor	Wild	Emperor Island, Antarctica	[51]	NA

We constructed 1 or more genomic libraries for each of the 19 penguin species depending on the DNA quality. For species that we could obtain high molecular weight DNA with the main band longer than 40 kb, we constructed 10X Genomics genomic libraries to produce 100× coverage sequencing data (Table 2). To do this, we attached a specific unique barcode to 1 end of short DNA fragments that are broken from 1 long DNA fragment, using standard protocols provided by Chromium™ Genome Solution. Because this protocol encompasses >1 million specific barcodes in a single solution, it decreases the chance of short DNA fragments with the same barcode being derived from unrelated long DNA fragments. For those species with shorter DNA fragments (<40 kb), we constructed genomic libraries following Illumina (San Diego, CA, [77]) or BGIseq 500 [78] protocols. Those protocols resulted in several paired-end libraries with insert sizes of either 250 or 500 bp, in addition to several mate-pair libraries with insert sizes ranging from 2 to 10 kb (Table 2). We further generated 100–320× coverage sequencing data for these species. Furthermore, we did not find any significant difference in the assembly quality between Illumina and BGIseq, while the 10x strategy normally produced better assembly than the other strategy with multiple insert-sized libraries (Table 3). Following sequencing, we generated 3.24 Tb sequencing reads encompassing all 19 penguin species, obtaining >111 Gb data per species (Table 2).

**Table 2:** Details of the sequencing platform used and the data statistics for 21 penguin genomes

Species	Library construction strategy	Sequencing platform	Raw data (Gb)	Clean data (Gb)
*Eudyptes chrysolophus chrysolophus*	10X	BGIseq500	145.9	126.9
*Megadyptes antipodes antipodes*	10X	BGIseq500	111.9	104.1
*Spheniscus demersus*	10X	BGIseq500	141.1	131.3
*Spheniscus mendiculus*	10X	BGIseq500	112.2	104.4
*Eudyptula minor albosignata*	10X	BGIseq500	132.5	124.8
*Eudyptula minor minor*	10X	BGIseq500	121.4	112.7
*Eudyptula novaehollandiae*	10X	BGIseq500	180.4	168.5
*Pygoscelis papua*	10X	BGIseq500	134.5	124.0
*Pygoscelis antarctica*	10X	BGIseq500	154.5	139.7
*Aptenodytes patagonicus*	10X	BGIseq500	147.6	134.0
*Eudyptes chrysolophus schlegeli*	250 bp, 2 kb, 5 kb, 10 kb	BGIseq500	402.6	296.6
*Eudyptes pachyrhynchus*	250 bp, 2 kb, 5 kb, 10 kb	HiSeq X ten and HiSeq 4000	146.4	104.7
*Eudyptes robustus*	250 bp, 2 kb	HiSeq X ten and HiSeq 4000	171.2	107.6
*Eudyptes sclateri*	250 bp, 2 kb, 5 kb	HiSeq X ten and HiSeq 4000	156.2	103.2
*Eudyptes filholi*	250 bp, 2 kb, 5 kb, 10 kb	HiSeq X ten and HiSeq 4000	195.0	146.8
*Eudyptes chrysocome*	250 bp, 2 kb, 5 kb	HiSeq X ten and HiSeq 4000	195.1	111.6
*Eudyptes moseleyi*	250 bp, 2 kb, 5 kb, 10 kb	HiSeq X ten and HiSeq 4000	173.6	133.1
*Spheniscus magellanicus*	250 bp, 2 kb, 5 kb, 10 kb	HiSeq X ten and HiSeq 4000	212.6	150.7
*Spheniscus humboldti*	250 bp, 2 kb, 5 kb, 10 kb	HiSeq X ten and HiSeq 4000	208.8	137.2

HiSeq X ten was used for sequencing small insert size libraries; HiSeq 4000 was used for sequencing mate-pair libraries.

**Table 3:** Assembly statistics and BUSCO results for 21 penguin genomes within a total of 4,915 conserved avian orthologs

Library construction strategy	Species	Contig N50 (bp)	Scaffold N50 (bp)	Genome size (bp)	Complete	Duplication	Fragmented	Missing
10x	*Eudyptes chrysolophus chrysolophus*	163,848	13,794,837	1,368,663,695	85.40%	7.70%	4.40%	2.50%
	*Megadyptes antipodes antipodes*	83,954	23,315,117	1,317,732,923	91.80%	1.20%	4.20%	2.80%
	*Spheniscus demersus*	101,408	15,386,364	1,278,371,924	91.30%	0.90%	4.70%	3.10%
	*Spheniscus mendiculus*	72,552	380,950	1,300,348,609	88.90%	1.60%	5.70%	3.80%
	*Eudyptula minor albosignata*	95,773	21,866,543	1,374,338,381	85.60%	7.40%	4.20%	2.80%
	*Eudyptula minor minor*	88,190	21,127,646	1,466,686,831	84.00%	8.60%	4.60%	2.80%
	*Eudyptula novaehollandiae*	122,461	29,280,209	1,357,427,560	89.00%	4.70%	3.80%	2.50%
	*Pygoscelis papua*	93,785	2,780,837	1,309,329,553	90.70%	1.50%	5.00%	2.80%
	*Pygoscelis antarctica*	118,336	6,180,260	1,265,661,676	91.30%	1.20%	4.60%	2.90%
	*Aptenodytes patagonicus*	116,769	2,903,810	1,256,739,118	91.50%	1.10%	4.20%	3.20%
Multi-libraries	*Eudyptes chrysolophus schlegeli*	24,191	1,877,548	1,310,605,488	93.20%	1.50%	3.30%	2.00%
	*Eudyptes pachyrhynchus*	33,319	8,795,033	1,310,923,788	80.20%	7.70%	4.30%	7.80%
	*Eudyptes robustus*	29,712	363,310	1,248,618,553	87.30%	1.10%	5.10%	6.50%
	*Eudyptes sclateri*	69,562	1,921,244	1,211,737,899	93.60%	1.10%	3.20%	2.10%
	*Eudyptes filholi*	74,280	6,429,221	1,223,976,468	93.20%	1.00%	3.60%	2.20%
	*Eudyptes chrysocome*	66,005	1,949,323	1,231,067,970	93.80%	1.00%	3.00%	2.20%
	*Eudyptes moseleyi*	21,362	2,248,088	1,306,699,575	93.60%	1.20%	3.00%	2.20%
	*Spheniscus magellanicus*	41,455	12,679,469	1,262,636,738	93.10%	1.30%	3.50%	2.10%
	*Spheniscus humboldti*	19,849	6,229,819	1,243,403,142	93.30%	1.10%	3.50%	2.10%
	*Pygoscelis adeliae*	22,195	5,118,896	1,216,600,033	92.80%	0.60%	4.00%	2.60%
	*Aptenodytes forsteri*	31,730	5,071,598	1,254,347,440	93.20%	0.80%	3.60%	2.40%

#### Genome assembly and quality evaluation

Sequences obtained from the 250-bp insert size libraries and the 10x libraries were used to evaluate the genome size for each penguin using a *k*-mer approach [79]. Reads were scanned using a 17-bp window with 1 bp sliding and the frequency of each 17 *k*-mer was recorded. After all the reads were scanned, the *k*-mer frequency distributions were plotted and the depth with the highest frequency (K_dep) was defined. The genome size was estimated as the read number * (read length – 17 + 1)/K_dep. The filtered reads for the 10x libraries were only used for estimating the genome size with 17 *k*-mer, while all reads were used for Supernova assembly.

Sequencing errors have a major effect on subsequent genome assembly because they both introduce mistakes in the assembly and also decrease the assembly continuities. Several features can be linked to sequencing noise, including low-quality bases, adaptor contamination, and duplication [80]. To remove the potential biases introduced by sequencing noise, we filtered our raw sequencing reads prior to genome assembly, following strict standards including (i) discarding paired-end reads containing overlaps, (ii) removing reads with >20% low-quality bases as the quality score was <10, (iii) removing reads with >5% ambiguous N bases, (iv) removing paired-end reads containing identical sequences likely to be PCR duplicates, and (v) removing reads with adaptor sequences. Following filtering, each genome contained >104 Gb data. Overall, we obtained a total of 2.56 Tb high-quality data for all 19 penguin genomes (Table 2).

Both SOAPdenovo v. 2–2.04 (SOAPdenovo2, RRID:SCR_014986) [81] and Allpaths-LG (ALLPATHS-LG, RRID:SCR_010742) [82] were used to assemble the genomic libraries from the various insert sizes. For SOAPdenovo, paired-end reads from small insert size libraries were used to construct de Bruijn graphs, with various *k*-mer ranging from 23 to 47. Contigs were subsequently constructed using contig modular with the “-D 1 -g” parameter to remove edges containing coverages no larger than 1. Following this, “map -k 35 -g” was used to map mate-pair reads into contigs, with *k*-mer size 35. Finally, we conducted scaffolding with parameters “scaff -g -F” to assemble the contigs into longer linkages. The best version, in terms of various *k*-mer in the graph construction step, was chosen as the SOAPdenovo representative for each species. In addition, we also assembled genomic libraries from various insert sizes using Allpaths-LG following the default parameters. By comparing the assemblies from both SOAPdenovo and Allpaths-LG, according to both the scaffold N50 and the total length, we chose the best assembler as a representative for each of the 19 penguin species. Supernova v. 2.0 [83], recommended for 10x genomic data [83], was used to assemble those species with 10x genomic libraries, following the default parameters. The optimal assembly strategy chosen for each penguin species is listed in Supplementary Table 2. For each assembly, we used GapCloser v. 1.12 (GapCloser, RRID:SCR_015026) [81] to locally assemble and close gaps within each scaffold following the default parameters.

**Figure 2:**
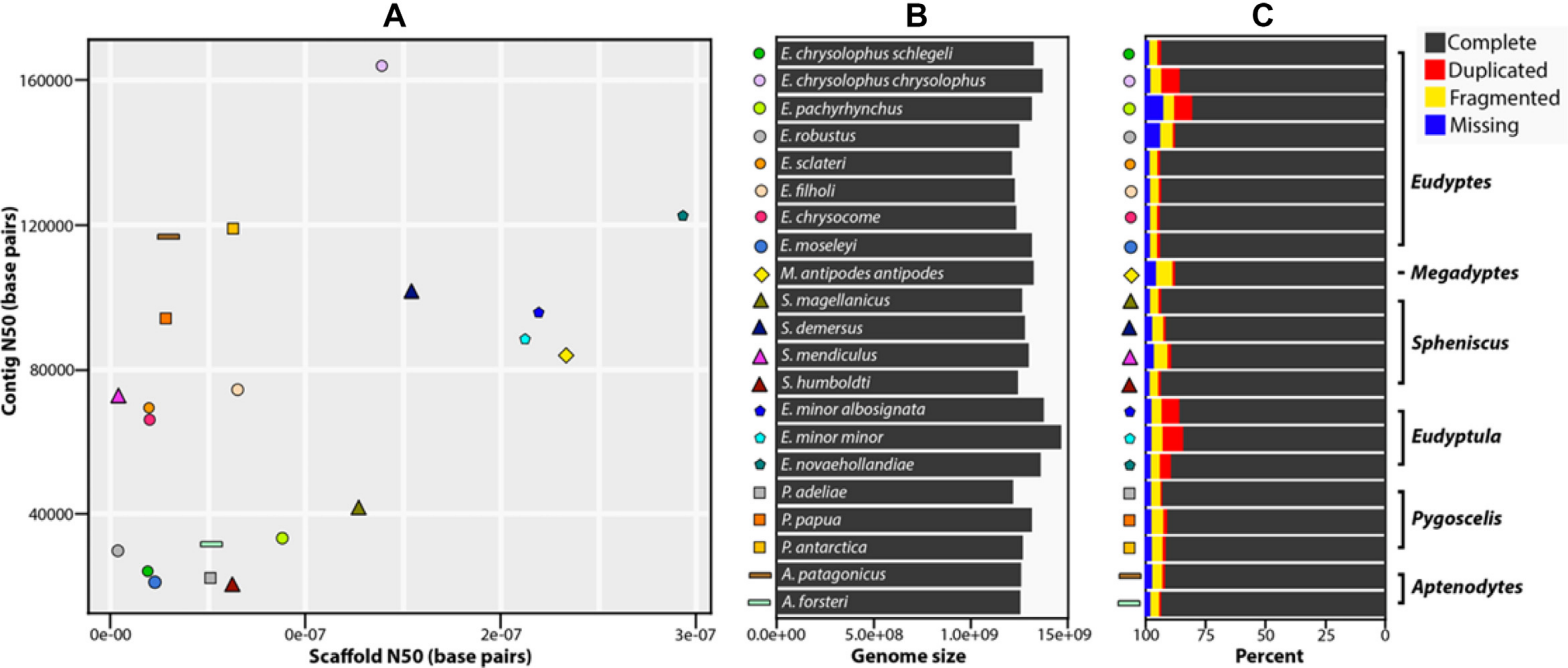
Genome assembly statistics of all penguin species. A, Dot plot of the quality of each index showing contig N50 (maximum is *Eudyptes chrysolophus chrysolophus* with 163,848 bp; minimum is *Spheniscus humboldti* with 19,849 bp) and scaffold N50 (maximum is *Eudyptula novaehollandiae* with 29,280,209 bp; minimum is *Eudyptes robustus* with 363,310 bp). Each symbol indicates a penguin species, the x-axis indicates the scaffold N50, and the y-axis indicates the contig N50 for each species. B, Genome size for each penguin species (maximum is *Eudyptula minor* with 1,466,686,831 bp; minimum is *Eudyptes sclateri* with 1,211,737,899 bp). C, BUSCO assessments of all penguin genomes, showing the percentage of complete, duplicated, fragmented, or missing data. See Table   3 for more details. The symbols for each penguin species correspond to the symbols used in Fig. 1. and Fig. 3.

All penguins (including those obtained in Li et al. [51]) were estimated to have a ∼1.3-Gb genome (Fig.   2), containing little variances. Most assemblies have both a longer scaffold N50 and contig N50 than the *Aptenodytes forsteri* and *Pygoscelis adeliae* assemblies obtained by Li et al. [51] (Fig. 2). In total, the 21 genomes contained a scaffold N50 >1 Mb, and of those, 13 genomes contained a scaffold N50 >3 Mb. All penguin genomes contain a contig N50 >19 kb and 15 of the genomes are >30 kb. The maximum contig N50 extends to 163 kb for the macaroni penguin (*Eudyptes chrysolophus chrysolophus*) (Fig. 2). The highest-quality genome is *Eudyptula novaehollandiae*, encompassing a 29.3-Mb scaffold N50. Therefore, our results demonstrate consistency and high quality among all 21 penguin genomes (Fig. 2).

The genome assembly completeness provides an evaluation of the assembly quality. We used BUSCO v. 3.0.2 (BUSCO, RRID:SCR_015008) [84] to evaluate our newly assembled penguin genomes with the avian database aves_odb9, which encompasses 4,915 conserved avian orthologs (Table 3). Only ∼3% of the core genes in aves_odb9 could not be annotated on the 21 penguin genomes (ranging between 2% and 7.8%). This demonstrates that all 21 penguin genomes are near-complete, containing only a few gaps. We identified an average of 90% complete core genes on each of the 21 penguin genomes, with the richest being 93.8% on *Eudyptes chrysocome*. Furthermore, when several genes were annotated in >1 copy, we considered them to be duplications. Duplication rates among the 21 penguin genomes varied only between 0.6% and 8.6%. In addition, only ∼4% of the core genes were partly annotated on each of the 21 penguin genomes (Fig. 2). Overall, we obtained almost-complete, high-quality genomes. Our genomic dataset (including those obtained in Li et al. [51]) encompasses all extant penguin species, representing a comprehensive dataset.

#### Repeat annotation

We used RepeatMasker v. 4.0.7 (RepeatMasker, RRID:SCR_012954) [85, 86], TRF v. 4.09 [87], and RepeatModeler v. 1.0.8 (RepeatModeler, RRID:SCR_015027) [88, 86] to identify repetitive sequences in each of the penguin genomes. We compared our genomes to 5 avian outgroups: wedge-rumped storm petrel (*Hydrobates tethys*), Wilson's storm petrel (*Oceanites oceanicus*), Atlantic yellow-nosed albatross (*Thalassarche chlororhynchos*), zebra finch (*Taeniopygia guttata*), and chicken (*Gallus gallus*). Genome sequences were aligned to RepBase23.04 [89] through RepeatMasker, and each hit was further classified into detailed categories. Tandem repeats, which are a series of DNA sequences containing >2 adjacent copies, were identified with TRF using the default parameters. In addition, we used RepeatModeler in a *de novo* repeat family identifying approach. All identified repeat elements were classified into 7 categories (DNA, long interspersed nuclear element [LINE], short interspersed nuclear element [SINE], long terminal repeat [LTR], other, unknown, tandem repeat) according to classification in repeat databases. Repeat annotations using the 3 methods were combined into a non-redundant repeat annotation for each penguin genome and the 5 outgroups.

Approximately 10% of the genome sequences were identified as repeat elements on each penguin genome, which is similar to the 5 outgroups (Table 2). Although all penguin genomes had similar repeat content, they varied in content for each category. In all penguins and outgroups, the most abundant repeat category was LINE. *E. moseleyi* has the richest tandem repeats of 3.52%, which is substantially greater than *Aptenodytes forsteri*, which has a richest tandem repeats of 2.24% and contains the second richest tandem repeats repeat in all penguins. *Eudyptula minor minor* had the most genome sequences identified as LTR (4.26%). See Table 4 for specific details on repeat annotations for each species.

**Table 4:** Repeat annotation results for 21 penguins and 5 outgroups

Species	DNA		LINE		SINE		LTR		Other		Unknown		TRF		Total	
	Length (bp)	% in genome	Length (bp)	% in genome	Length (bp)	% in genome	Length (bp)	% in genome	Length (bp)	% in genome	Length (bp)	% in genome	Length (bp)	% in genome	Length (bp)	% in genome
*Eudyptes chrysolophus schlegeli*	10,967,993	0.84	56,600,258	4.32	1,886,042	0.14	23,772,820	1.81	1,709	0.00013	7,181,843	0.55	27,041,073	2.06	122,778,314	9.37
*Eudyptes chrysolophus chrysolophus*	9,840,577	0.72	81,007,897	5.92	2,325,630	0.17	42,950,488	3.14	2,109	0.00015	6,349,669	0.46	7,624,752	0.56	147,221,283	10.80
*Eudyptes pachyrhynchus*	9,700,549	0.74	57,537,411	4.39	1,761,671	0.13	26,951,871	2.06	7,163	0.00055	8,778,995	0.67	15,315,109	1.17	115,154,499	8.78
*Eudyptes robustus*	10,035,161	0.80	54,876,908	4.40	1,694,896	0.14	21,900,240	1.75	1,197	0.000096	6,793,784	0.54	13,082,350	1.05	105,161,038	8.42
*Eudyptes sclateri*	9,603,106	0.79	57,388,336	4.74	1,648,534	0.14	22,555,283	1.86	2,155	0.00018	5,455,896	0.45	7,045,858	0.58	101,615,942	8.39
*Eudyptes filholi*	9,447,824	0.77	58,471,185	4.78	1,894,915	0.16	23,146,953	1.89	2,662	0.00022	8,146,713	0.67	7,812,634	0.64	104,766,914	8.56
*Eudyptes chrysocome*	9,067,962	0.74	58,040,264	4.71	1,608,644	0.13	22,515,809	1.83	2,095	0.00017	7,321,722	0.60	7,332,611	0.60	103,276,447	8.39
*Eudyptes moseleyi*	9,367,954	0.72	58,805,425	4.50	1,990,469	0.15	23,593,767	1.81	2,664	0.00020	9,786,633	0.75	45,959,293	3.52	141,103,330	10.80
*Megadyptes antipodes antipodes*	9,608,349	0.73	78,978,618	5.99	1,728,524	0.13	46,464,418	3.53	1,059	0.000080	8,168,785	0.62	7,802,048	0.59	148,977,693	11.30
*Spheniscus magellanicus*	10,393,349	0.82	65,351,067	5.18	1,812,355	0.14	26,759,543	2.12	1,546	0.00012	9,851,237	0.78	10,398,934	0.82	118,099,179	9.35
*Spheniscus demersus*	9,811,467	0.77	72,969,293	5.71	1,610,171	0.13	34,709,683	2.72	1,509	0.00012	20,385,557	1.59	6,712,698	0.53	130,219,709	10.2
*Spheniscus mendiculus*	10,792,037	0.83	80,340,773	6.18	1,694,428	0.13	43,906,026	3.38	2,265	0.00017	13,023,335	1.00	7,421,979	0.57	147,721,431	11.4
*Spheniscus humboldti*	9,850,523	0.80	63,427,971	5.10	2,095,439	0.17	26,032,187	2.09	2,610	0.00021	7,051,364	0.57	10,846,563	0.87	115,794,679	9.31
*Eudyptula minor albosignata*	10,287,254	0.75	86,732,446	6.31	2,230,442	0.16	49,548,759	3.61	2,285	0.00017	10,370,641	0.76	8,661,285	0.63	160,541,239	11.70
*Eudyptula minor minor*	10,691,141	0.73	95,293,482	6.50	1,790,448	0.12	62,515,534	4.26	2,245	0.00015	8,460,299	0.58	9,083,782	0.62	183,740,284	12.5
*Eudyptula novaehollandiae*	10,542,998	0.78	87,757,466	6.46	1,654,900	0.12	53,144,657	3.92	1,522	0.00011	12,914,720	0.95	8,531,830	0.63	164,989,801	12.20
*Pygoscelis adeliae*	8,905,965	0.73	52,089,816	4.28	1,643,684	0.14	17,580,686	1.45	1,685	0.00014	6,938,950	0.57	8,565,483	0.70	93,839,128	7.71
*Pygoscelis papua*	10,878,036	0.83	79,578,503	6.08	1,683,574	0.13	47,004,788	3.59	2,163	0.00017	8,393,877	0.64	7,857,958	0.60	151,240,877	11.60
*Pygoscelis antarctica*	10,021,109	0.79	75,467,782	5.96	1,660,023	0.13	36,515,988	2.89	1,645	0.00013	5,649,521	0.45	6,850,733	0.54	133,620,728	10.60
*Aptenodytes patagonicus*	9,883,830	0.79	72,143,844	5.74	1,669,248	0.13	33,210,718	2.64	2,273	0.00018	5,987,857	0.48	6,868,165	0.55	126,913,554	10.10
*Aptenodytes forsteri*	9,648,988	0.77	47,421,228	3.78	1,755,252	0.14	14,998,979	1.20	1,055	0.000084	5,984,114	0.48	28,075,518	2.24	103,411,467	8.24
*Hydrobates tethys*	10,174,835	0.85	43,642,750	3.65	1,593,248	0.13	13,363,132	1.12	1,780	0.00015	6,044,078	0.51	10,375,034	0.87	82,871,365	6.93
*Oceanites oceanicus*	8,172,757	0.69	53,982,174	4.58	1,518,213	0.13	19,561,601	1.66	2,202	0.00019	6,101,243	0.52	10,501,141	0.89	97,111,623	8.24
*Thalassarche chlororhynchos*	10,390,449	0.93	41,856,139	3.74	1,766,094	0.16	14,374,696	1.29	2,035	0.00018	5,822,959	0.52	6,943,803	0.62	79,491,403	7.11
*Taeniopygia guttata*	5,985,051	0.49	51,144,902	4.15	883,324	0.072	50,817,604	4.12	4,713	0.00038	13,099,829	1.06	25,800,776	2.09	137,289,217	11.10
*Gallus gallus*	13,929,789	1.33	78,779,279	7.52	571,067	0.055	21,043,114	2.01	1,638	0.00016	20,514,532	1.96	10,603,861	1.01	129,394,288	12.40

#### Protein-coding gene annotation

We used the annotation methods developed by The Bird 10,000 Genomes (B10K) consortium [90] to annotate the 21 penguin genomes. Prior to annotating the protein-coding genes, a non-redundant avian reference gene set, consisting of protein sequences from *Taeniopygia guttata* and *Gallus gallus*, was generated [71]. Whole-genome protein sequences of Ensembl gene sets (release-85) of *Taeniopygia guttata*and *Gallus gallus* were then used to identify 12,337 orthologs based on whole-genome synteny relationships that were downloaded from the UCSC Genome Browser [91]. For both *Taeniopygia guttata* and *Gallus gallus*, we compared the 2 proteins in each ortholog and chose the longer homologous sequence with the human ortholog protein sequence in the reference gene set. Within 12,337 orthologs, 6,888 from *Taeniopygia guttata* and 5,449 from *Gallus gallus* were selected as the reference gene set. Following this, specific genes of *Taeniopygia guttata* or *Gallus gallus* were added to the reference gene set. This reference gene set comprised 5,084 *Taeniopygia guttata* genes without *Gallus gallus* orthologs and 3,158 *G. gallus* genes that had not been identified as ortholog genes to *Taeniopygia guttata*. Finally, protein sequences were filtered if they contained <50 amino acids, consisted of function as transposons/retrotransposons, or contained only a single non-functional exon. The final avian reference gene set therefore contained 20,181 protein-coding genes.

To annotate the protein-coding genes from the penguin genomes, protein sequences from the avian reference gene set were then mapped to each of the 21 penguin genomes. First, protein sequences were aligned to each penguin genome using TBLASTN v. 2.2.2 (TBLASTN, RRID:SCR_011822) [92] with a 1e−5 e-value cut-off. Multiple adjacent hits from the same protein were then linked together using genBlastA v. 1.0.4 [93] to obtain the candidate gene boundary. A candidate hit was removed if a protein had <30% amino acids aligned to the penguin genome. For each candidate hit for each protein, we extracted genomic sequences covering this hit with 2 kb upstream and downstream of the extension. Extracted genome sequences and corresponding homologous protein sequences were then prepared as input for GeneWise v. 2.4.1 (GeneWise, RRID:SCR_015054) [94] to the annotated protein-coding gene models, which included exon and intron boundaries. Coding sequences for each annotated gene model were extracted from each genome according to the annotated gene model, and then each coding sequence was translated into the protein sequence. This annotated protein sequence was then aligned with the corresponding homolog protein sequence using MUSCLE v. 3.8.31 (MUSCLE, RRID:SCR_011812) [95], while removing annotated proteins with <40% identity with the corresponding homolog protein sequence. Annotated proteins with <30 amino acids and annotated proteins containing >2 frame shifts or 1 premature stop codon were then removed. If a genome locus had been annotated using several gene models, the gene model with the highest identity with the corresponding homolog protein was selected. Therefore, the annotated gene set for our penguin genomes contained no overlapping genes.

Protein sequences from human (hg38) and avian transcripts were also mapped to each penguin genome and the annotated gene models (as above). For the avian transcripts dataset, we obtained 71 avian transcriptomic samples from NCBI [96] (Supplementary Table 3) and assembled those into transcripts using either Newbler v2.9 [97] for 454 sequencing assemblies or Trinity v20140717 [98] for Illumina sequencing assemblies. We used ORFfinder [96] to identify open reading frames (ORFs) for transcripts, and the protein sequences were then translated from the ORF. The protein sequences translated from the transcripts were then mapped to the avian reference gene set and the human protein sequences, while removing those with similarity to the avian reference gene set or the human protein sequences. Transcripts with ORF length <150 bp were also removed. Protein sequences from 5,257 transcripts were then used for annotation. Three gene model sets annotated from the avian reference gene set, the human protein sequences, and transcriptome were then combined into a final non-redundant gene set. We prioritized 3 gene model sets in the following order: avian reference gene set > human protein > transcriptome.

After applying the above methods, we annotated the 19 newly assembled penguin genomes, as well as the 2 previously published penguin genomes [51]. We identified ∼16,000 genes on each penguin genome, which is similar to the genomes of *Taeniopygia guttata* and *Gallus gallus*. The average gene length and coding sequence length are ∼19 and 1.3 kb, respectively. Each gene encompasses ∼8 exons, with an average length of 170 bp. Intron lengths are an average length of 2.6 kb (Table 5).

**Table 5:** Protein-coding gene statistics of all 21 penguin genomes and 5 outgroups

Species	Number of protein-coding genes	Mean gene length (bp)	Mean coding sequence length (bp)	Mean exons per gene	Mean exon length (bp)	Mean intron length (bp)
*Eudyptes chrysolophus schlegeli*	17,191	18,860	1,351	7.9	171	2,540
*Eudyptes chrysolophus chrysolophus*	16,311	20,248	1,392	8.2	170	2,623
*Eudyptes pachyrhynchus*	19,170	17,394	1,306	7.4	178	2,535
*Eudyptes robustus*	17,126	16,254	1,295	7.4	174	2,329
*Eudyptes sclateri*	15,786	19,627	1,402	8.2	171	2,527
*Eudyptes filholi*	15,963	19,959	1,407	8.2	171	2,562
*Eudyptes chrysocome*	16,280	19,436	1,382	8.1	171	2,555
*Eudyptes moseleyi*	16,812	19,767	1,370	8.0	171	2,621
*Megadyptes antipodes antipodes*	16,563	18,509	1,334	7.8	171	2,533
*Spheniscus magellanicus*	16,795	19,311	1,381	8.1	171	2,535
*Spheniscus demersus*	16,134	19,029	1,344	7.8	171	2,584
*Spheniscus mendiculus*	16,390	17,097	1,311	7.6	172	2,382
*Spheniscus humboldti*	16,587	19,642	1,387	8.1	170	2,558
*Eudyptula minor albosignata*	17,424	18,837	1,338	7.8	172	2,574
*Eudyptula minor minor*	17,802	19,078	1,349	7.8	172	2,598
*Eudyptula novaehollandiae*	17,188	19,271	1,355	7.9	172	2,609
*Pygoscelis adeliae*	14,463	20,595	1,385	8.3	168	2,648
*Pygoscelis papua*	16,698	18,276	1,333	7.8	172	2,503
*Pygoscelis antarctica*	15,488	19,520	1,381	8.1	171	2,558
*Aptenodytes patagonicus*	15,195	19,596	1,384	8.1	170	2,552
*Aptenodytes forsteri*	15,593	19,844	1,381	8.1	170	2,584
*Hydrobates tethys*	15,915	17,898	1,344	8.1	165	2,323
*Oceanites oceanicus*	16,055	17,936	1,356	8.0	170	2,377
*Thalassarche chlororhynchos*	13,347	10,029	1,110	6.4	175	1,667
*Taeniopygia guttata*	19,174	14,787	1,196	7.2	167	2,198
*Gallus gallus*	17,883	16,965	1,414	8.3	171	2,135

#### Gene function annotation

To assign functions to each gene, we aligned each gene to 3 functional databases: Swiss-Prot release-2019_03 [99], InterPro v. 68.0 (InterPro, RRID:SCR_006695) [100], and KEGG v89.1 (KEGG, RRID:SCR_012773) [101]. Protein sequences of each gene were aligned to Swiss-Prot database using BLASTP [92], and the function of the best hit was selected as the function annotation for this gene. We then searched InterPro databases that encompass ProDom, PRINTS, Pfam, SMART, PANTHER, ProSiteProfiles, and ProSitePatterns to obtain the motifs and domains for each gene. Gene Ontology [102] terms for each gene were obtained from the corresponding InterPro entry. To identify the pathways in which the gene might be involved, protein sequences for each gene were then aligned against the KEGG database using BLASTP. For each penguin genome, a total of >99% of the protein-coding genes were assigned ≥1 function annotation in each penguin, which is similar to the 5 outgroups (Table 6). Overall, >95% of the protein genes were assigned a Swiss-Prot function, demonstrating high-quality gene sets.

**Table 6:** Function annotation results for protein-coding genes for 21 penguins and 5 outgroups

Species	Swissprot		KEGG		Interpro		Overall	
	Number	%	Number	%	Number	%	Number	%
*Eudyptes chrysolophus schlegeli*	16,739	97.37	15,347	89.27	16,916	98.40	17,064	99.26
*Eudyptes chrysolophus chrysolophus*	15,863	97.25	14,646	89.79	16,051	98.41	16,191	99.26
*Eudyptes pachyrhynchus*	18,680	97.44	17,250	89.98	18,873	98.45	19,028	99.26
*Eudyptes robustus*	16,580	96.81	15,500	90.51	16,816	98.19	16,988	99.19
*Eudyptes sclateri*	15,383	97.45	14,172	89.78	15,540	98.44	15,664	99.23
*Eudyptes filholi*	15,555	97.44	14,362	89.97	15,696	98.33	15,840	99.23
*Eudyptes chrysocome*	15,692	96.39	14,732	90.49	15,977	98.14	16,148	99.19
*Eudyptes moseleyi*	16,377	97.41	15,153	90.13	16,540	98.38	16,688	99.26
*Megadyptes antipodes antipodes*	15,755	95.12	14,993	90.52	16,264	98.19	16,445	99.29
*Spheniscus magellanicus*	16,371	97.48	15,136	90.12	16,532	98.43	16,670	99.26
*Spheniscus demersus*	15,388	95.38	14,579	90.36	15,839	98.17	16,001	99.18
*Spheniscus mendiculus*	15,714	95.88	14,801	90.31	16,090	98.17	16,254	99.17
*Spheniscus humboldti*	16,172	97.50	14,954	90.15	16,319	98.38	16,460	99.23
*Eudyptula minor albosignata*	16,615	95.36	15,778	90.55	17,098	98.13	17,297	99.27
*Eudyptula minor minor*	16,994	95.46	16,073	90.29	17,476	98.17	17,663	99.22
*Eudyptula novaehollandiae*	16,423	95.55	15,561	90.53	16,892	98.28	17,060	99.26
*Pygoscelis adeliae*	13,964	96.55	13,054	90.26	14,220	98.32	14,348	99.20
*Pygoscelis papua*	15,931	95.41	15,097	90.41	16,378	98.08	16,553	99.13
*Pygoscelis antarctica*	15,050	97.17	13,853	89.44	15,224	98.30	15,360	99.17
*Aptenodytes patagonicus*	14,808	97.45	13,493	88.80	14,954	98.41	15,063	99.13
*Aptenodytes forsteri*	15,053	96.54	14,112	90.50	15,308	98.17	15,478	99.26
*Hydrobates tethys*	15,493	97.35	14,273	89.68	15,628	98.20	15,775	99.12
*Oceanites oceanicus*	15,622	97.30	14,412	89.77	15,775	98.26	15,919	99.15
*Thalassarche chlororhynchos*	12,958	97.09	11,881	89.02	13,072	97.94	13,219	99.04
*Taeniopygia guttata*	18,367	95.79	17,115	89.26	18,537	96.68	18,918	98.66
*Gallus gallus*	16,760	93.72	15,585	87.15	17,079	95.50	17,263	96.53

#### Phylogenomic reconstruction

To understand the evolutionary history of all extant penguins, we created a phylogeny of penguins using the genomic-level orthologs with coalescent-based ExaML and concatenation-based methods MP-EST and ASTRAL [103–105]. We first applied rigorous filtering steps to obtain 7,235 high-quality orthologs. This was achieved by filtering ∼13,214 orthologs (BLAST reciprocal best hits [RBHs]) that were present in the *Taeniopygia guttata* genome and the 21 penguins/5 avian outgroup genomes (described above), retaining orthologs with no missing data, and removing sequences containing internal stop codons. We aligned and filtered our alignment data using several methods: (i) protein sequences were aligned using MAFTT v. 7.313 [106] following “linsi” parameters for local, iterative progressive alignment; (ii) we also applied column-based alignment filtering using trimAl v. 1.4.rev22 [107], using the parameter “automated1” to heuristically choose trimming parameters based on input alignment characters; (iii) nucleic acid alignments were also obtained using trimAl, using the parameter “backtrans” to obtain a back-translation for a given amino acid alignment. Alignment filtering was applied to (i) the column-based alignments, by removing all missing data, and retaining alignment lengths >50 bp (resulting in 7,229 orthologs, the “TrimAl data” set); and (ii) applying a full-matrix occupancy to the no missing dataset (retaining 7,011 orthologs, the “No missing data” set) following the pipeline published previously [108]. Loci containing no missing taxa were then retained, by removing alignment columns containing gaps, undetermined bases (Ns), or ambiguity characters and loci with a post-filtering alignment length <200 bp.

We constructed gene trees for each locus using RAxML v8.2.12 (RAxML, RRID:SCR_006086) [109] and then constructed phylogenomic trees using 2 coalescent-based methods, MP-EST v. 2.0 and ASTRAL-III, based on the gene trees. First, we used RAxML v. 8.2.12 to infer the highest-scoring maximum likelihood tree from unpartitioned alignments for each locus using a GTR+GAMMA substitution model, 20 independent tree searches beginning from random starting tree topologies, and 500 bootstrap replicates for each locus. Resulting gene trees were rooted with *Gallus gallus* using the “ape” package in R v. 3.5.2 [110]. We then created a coalescent-based phylogenetic tree using MP-EST v. 2.0 [104] by estimating trees from a set of rooted gene trees by maximizing a pseudo-likelihood function. Species tree and bootstrap topology searches were achieved over 3 independent replicates, using a different starting seed and with 10 independent tree searches per run. The highest-scoring tree in 10 tree searches was kept as the result for each replicate. Because the 3 final trees from MP-EST replicates shared the same tree topology, we kept the highest-scoring tree as the final tree for further analysis. Branch lengths were re-estimated in coalescent units of substitutions per site by constraining alignments to the MP-EST tree topology using the “-f E” option in ExaML v.3.0.21 [103]. Bootstrap values were plotted using RAxML based on the bootstrap replicates, and trees were outgroup-rooted with *G. gallus*. In addition, we used the coalescent-based method ASTRAL-III [105] with default parameters to obtain the tree with the maximum number of shared induced quartet trees in the set of unrooted gene trees, constrained by the set of bipartitions in the tree based on a predefined set of partitions. The inferenced trees also shared the same tree topology with the MP-EST results. Then, the concatenation-based phylogenomic inference was conducted using ExaML v3.0.21. This was achieved using a GTR+GAMMA substitution model on the partitioned (each locus as a separate partition), concatenated alignments, and inferring the topology from 21 full maximum likelihood tree searchers: 20 beginning with random starting trees, and a single search beginning with the random stepwise addition order parsimony tree conducted using RAxML. For each dataset, 100 ExaML bootstrap replicates were conducted and convergence was assessed according to the bootstrapping analysis and applying a majority-rule consensus tree criterion in RAxML with option “-I autoMRE”. We then compared the resulting trees obtained using the “TrimAl data” and the “No missing data” from coalescent-based MP-EST and ASTRAL with concatenation-based ExaML (Supplementary Fig. 1).

**Figure 3:**
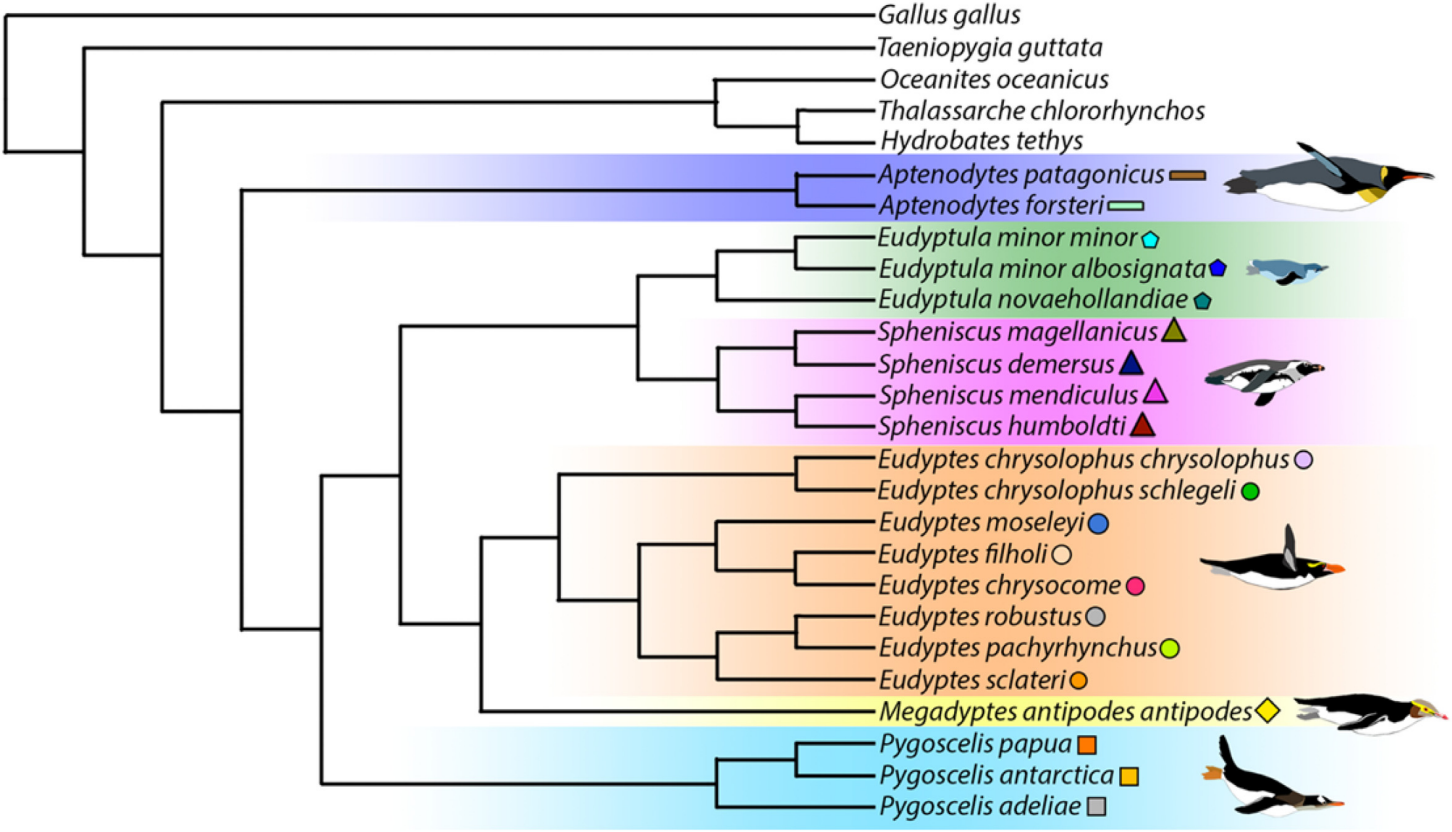
Phylogenomic reconstruction of penguins inferred by the ExaML method with no missing data. The topology of all clades was strongly supported (bootstrap support: 100). The topology and support were identical using the MP-EST and ASTRAL methods (with no missing data) except for the outgroup (bootstrap support for the split between *Hydrobates tethys* and *Oceanites oceanicus*: 37) and within the penguin genus *Spheniscus* (bootstrap support for the split between the African penguin [*Spheniscus demersus*] and the magellanic penguin [*S. magellanicus*]: 97).

While the resulting topologies of the outgroups *Hydrobates tethys, Oceanites oceanicus*, and *Thalassarche chlororhynchos* are slightly different between coalescent-based and concatenation-based methods, the topologies of our penguin genomes are identical using both methods (Fig. 3). Our final phylogeny (Fig. 3) encompassing all extant penguin genomes is slightly different to a recent phylogenetic study using mitochondrial genomes [3]. Specifically, while the mitochondrial phylogeny suggested that *Aptenodytes*+*Pygoscelis* are sister to all other penguins, our full genome phylogeny suggests that *Aptenodytes* alone is sister to all other penguins. This result confirms earlier results combining data from a small set of mitochondrial genes and the nuclear *RAG-1*gene [1, 62] and provides intriguing new evidence on the historical biogeographical and evolutionary patterns of adaptation to Antarctica. We expect this novel genomic dataset to provide further important insights into the evolution of penguins in the southern hemisphere.

## Re-use Potential

### Consortium organization and further research plans

The 19 high-coverage genomes presented here, along with the *Aptenodytes forsteri* and *Pygoscelis adeliae* genomes presented by members of our consortium in 2014 [51], provide an exciting resource for understanding evolutionary diversification, the molecular basis for unique functional adaptation, and demographic histories of penguins. The Penguin Genome Consortium is an international team of scientists with backgrounds in marine ornithology, ecology, molecular biology, evolutionary and comparative genomics, phylogenetics, physiology, palaeontology, veterinary science, and bioinformatics. The diverse skills encompassed within our highly collaborative consortium will be essential to study these genomes under comparative genomic and evolutionary frameworks. In doing so, we will expand on [51] by investigating 3 key areas related to penguin evolution and adaptation.

#### Evolutionary relationships and taxonomic boundaries

With a deep evolutionary history, and diverse radiation, penguins provide an exciting system to understand the evolutionary drivers of diversification [3]. Moreover, robust taxonomic frameworks can be crucial for directing limited conservation resources for maximum gains. Significant uncertainty remains regarding species/lineage boundaries between some closely related penguin taxa. The genomes generated here therefore provide an exciting new dataset to examine taxonomic, phylogenomic, and biogeographical patterns for understanding penguin evolution.

#### Comparative genomics and adaptation

Penguins provide an excellent system to study comparative evolutionary adaptation [51]. We will use our genomes to explore comparative evolution among penguins, and between penguins and other avian orders. By examining loci under positive selection, we shall reveal the molecular basis for the unique physiological and morphological adaptations to different environments and ecologies that are exhibited by penguins.

#### Penguins in a changing world

Penguins are sensitive indicators of environmental change [44, 45]. It is predicted that future climate change will lead to significant declines in many penguin populations [47–50]. Conservation management decisions can be guided by demographic assessments. However, there remains a substantial gap in predicting ecosystem-wide changes to future climate change. As such, demographic analyses of these genomes will be critical for conservation management of penguins and other Southern Ocean assemblages.

### Cultural significance

The context in which wildlife research in New Zealand is undertaken is evolving rapidly and heading into new legal and novel cultural contexts [111–114]. Recent initiatives such as the bestowing of the rights of an individual on Te Urewera, a former national park, set an international precedent for this change in approach [115]. Therefore, it is critical that research permissions be obtained and appropriate indigenous consultation with Iwi, Rūnanga, Whānau, and Hapū be conducted. The regulatory arm of the New Zealand government in this process, the Department of Conservation, is legally required to give effect to the Principles of the Treaty of Waitangi [116] in its administration of the legislation pursuant to which Authorities are issues.

At another level the Ngāi Tahu Deed of Settlement Act recognizes all native penguin species as Taonga, or treasured possessions [117]. Consequently, not only is it a legal requirement to undertake rigorous Māori consultation when studying Taonga [118, 119], the Department of Conservation has to have particular regard to the views of Iwi, Rūnanga, Whānau, or Hapū when considering whether to authorize any application. Recent discussions have also emphasized that Taonga genomes are sacred (tapu) because they are considered to contain both the living and the future generations (whakapapa, mauri, and wairua of tipuna), engendering Māori concerns surrounding the commercialization, ownership, storage, and modification of Taonga genomes [120]. We generated Taonga genomes encompassing hoiho (yellow-eyed penguin, *Megadyptes antipodes antipodes*), kororā (little penguin, *Eudyptula* spp.), pokotiwha (Snares-crested penguin, *E. robustus*), tawaki (Fiordland-crested penguin, *E. pachyrhynchus*), and erect-crested penguin (*Eudyptes sclateri*). These genomes were obtained following rigorous Department of Conservation permitting procedures (including collection, holding, and exporting permits) and following Department of Conservation Iwi, Rūnanga, Whānau, or Hapū consultation (Supplementary Table 1). Several of the Taonga genomes studied here were collected alongside broader research projects, and additional consultation efforts were undertaken for those projects. We emphasize that there will be no commercialization, ownership, or modification of any of the genomes presented here. While these Taonga genomes will be publicly available, it is critical that new researchers studying these genomes take the appropriate steps to seek additional Māori permissions and consultation, which will ensure respect of New Zealand cultural values.

The emerging issues surrounding the generation and use of Taonga genomes also highlight that Māori consultation should also be undertaken when obtaining genomes from Taonga housed in overseas museum collections. We hope that the data and our research questions presented here, and our future research outputs using these genomes will be valuable for both cultural heritage and for conservation management of penguin populations.

### Early-release use of the data

The Fort Lauderdale [121] and Toronto [122] agreements state that in exchange for early release of datasets, the data producers retain the right to be the first to describe and analyse the complete datasets in peer-reviewed publications. Comparative and evolutionary genomic analyses are currently being carried out, and the consortium welcomes new members interested in contributing to this work. While this work is still underway we have published these 19 penguin genomes to provide early access, while requesting researchers intending to use these data for similar cross-species comparisons to continue to follow the long-running Fort Lauderdale and Toronto rules.

## Conclusions

Genomics is prohibitively costly—it requires high-quality samples and extensive laboratory and bioinformatic skills. The genomics era has been boosted by global research consortiums, which bring together contextual, technical, and analytical skills spanning a network of international collaborations [123–126]. Our consortium and dataset introduced here are no exception, and as such, we expect our future research using these genomes to bring together additional collaborators that encompass a wide range of expertise regarding penguin biology and physiology. At another level, collecting high-quality fresh blood samples from some of the most remote regions in the Southern Ocean remains technically and logistically difficult, requiring the efforts and long-term organization from many collaborations and expedition programs. While this study is an exciting development for understanding the evolution of penguins, the global efforts involved in designing our study, obtaining samples, and developing appropriate sequencing and bioinformatic pipelines have been extensive. The dataset and project design introduced here highlight the need for transparent research projects and global collaborations, which together maximize the use of samples, minimizing sequencing costs, and laboratory and analytical efforts.

In this study we have presented 19 new high-coverage penguin genomes. Together with 2 genomes previously obtained by members of our consortium [51], this combined dataset encompasses the genomes of all extant penguin species. We have also constructed a comprehensive phylogenomic tree encompassing all extant penguins. We will use these datasets to address a range of evolutionary, adaptive, biogeographic, and demographic questions regarding penguins. As such, we hope not only that our ongoing projects that encompass these genomes will provide novel insights for understanding the broad evolution and adaptation of avifauna to different environments but also that this knowledge will increase cultural heritage and aid conservation management decisions for remote Southern Ocean regions.

## Availability of supporting data and materials

The genome sequencing data and assemblies of this study have been deposited in the CNSA (https://db.cngb.org/cnsa/) of the CNGBdb database with the accession number CNP0000605, as well as the NCBI database with the Bioproject ID PRJNA556735 (*Aptenodytes patagonicus*: SAMN12384866; *Eudyptes chrysolophus chrysolophus*: SAMN12384869; *E. c. schlegeli*: SAMN12384870; *E. chrysocome*: SAMN12384872; *E. filholi*: SAMN12384873; *E. moseleyi*: SAMN12384871; *E. pachyrhynchus*: SAMN12384875; *Eudyptes robustus*: SAMN12384876; *E. sclateri*: SAMN12384874; *Eudyptula minor albosignata*: SAMN12384880; *E. m. minor*: SAMN12384879; *E. novaehollandiae*: SAMN12384878; *Megadyptes antipodes antipodes*: SAMN12384877; *Pygoscelis antarctica*: SAMN12384868; *P. papua*: SAMN12384867; *Spheniscus demersus*: SAMN12384881; *S. humboldti*: SAMN12384883; *S. magellanicus*: SAMN12384882; *S. mendiculus*: SAMN12384884. Data from all of the penguin species are also available from the *GigaScience* GigaDB database [127].

## Additional files

Supplementary Figure 1: Phylogenomic trees.

Supplementary Table 1: Sampling and permitting details of all penguin samples tested.

Supplementary Table 2: Assemblers and Kmer sizes used for each penguin.

Supplementary Table 3: Information of 71 avian transcriptomic samples downloaded from NCBI.

giz117_GIGA-D-19-00280_Original_Submission

giz117_GIGA-D-19-00280_Revision_1

giz117_Response_to_Reviewer_Comments_Original_Submission

giz117_Reviewer_1_Report_Original_Submission Hyun Park -- 8/14/2019 Reviewed

giz117_Reviewer_2_Report_Original_Submission Taras K Oleksyk, Ph.D. -- 8/26/2019 Reviewed

giz117_Supplemental_File

## Abbreviations

BLAST: Basic Local Alignment Search Tool; bp: base pairs; BUSCO: Benchmarking Universal Single-Copy Orthologs; CNSA: CNGB Nucleotide Sequence Archive; ExaML: Exascale Maximum Likelihood; Gb: gigabase pairs; kb: kilobase pairs; KEGG: Kyoto Encyclopedia of Genes and Genomes; LINE: long interspersed nuclear element; LTR: long terminal repeat; Mb: megabase pairs; NCBI: National Center for Biotechnology Information; ORF: open reading frame; RAxML: Randomized Axelerated Maximum Likelihood; SINE: short interspersed nuclear element; TRF: Tandem Repeat Finder; UCSC: University of California Santa Cruz.

## Ethics approval and consent to participate

All samples were obtained under valid animal ethics permits.

## Competing interests

The authors declare that they have no competing interests.

## Funding

This project was supported by the National Key R&D Program of China (MOST) grant 2018YFC1406901 and by the Science, Technology and Innovation Commission of Shenzhen Municipality grant No. JCYJ20170817150721687 and JCYJ20170817150239127. T.L.C. was supported by an Otago University postgraduate publishing bursary. G.Z. was supported by the Lundbeckfonden (grant No. R190–2014-2827), Carlsbergfondet (grant No. CF CF16–0663), the Villum Foundation (grant No. 25900), and by the Strategic Priority Research Program of the Chinese Academy of Science (grant No. XDB13000000, XDB31020000). M.T.P.G. was supported by the ERC Consolidator Grant 681396 “Extinction Genomics”.

## Authors’ contributions

G.Z. developed the concept; G.Z., D.-X.Z., T.L.C., and H.P. designed the project and wrote the manuscript; L.S.A., J.L.B., M.F.B., P.D.B., T.L.C., Y.C., P.D., U.E., S.R.F., S.G., D.M.H., P.H., T.H., E.K., K.L., G.M., T.M., L.J.N., P.P., P.G.R., D.R.T., H.T., and M.J.Y. collected and/or provided samples; J.L.B., T.L.C., A.H.R., T.H., K.J., B.M., T.S., D.R.T., and G.Z. facilitated sample collection; H.P., S.R.F., M.R.E., M.-H.S.S., and G.P. undertook laboratory work. H.P., X.B., M.F., C.Z., and Z.Y. undertook the bioinformatics work; G.Z., T.L.C., H.P., D.T.K., C.-A.B., M.R.E., P.G.B., M.T.P.G., T.H., J.F.M., R.A.P., A.J.D.T., L.D.S., M.-H.S.S., and P.Q. helped design sampling and project directions. All authors contributed to the final manuscript.
