# Correction to: High-coverage genomes to elucidate the evolution of penguins

**DOI:** 10.1093/gigascience/giaa031

**Published:** 2020-03-19

**Authors:** Hailin Pan, Theresa L Cole, Xupeng Bi, Miaoquan Fang, Chengran Zhou, Zhengtao Yang, Daniel T Ksepka, Tom Hart, Juan L Bouzat, Lisa S Argilla, Mads F Bertelsen, P Dee Boersma, Charles-Andre Bost, Yves Cherel, Peter Dann, Steven R Fiddaman, Pauline Howard, Kim Labuschagne, Thomas Mattern, Gary Miller, Patricia Parker, Richard A Phillips, Petra Quillfeldt, Peter G Ryan, Helen Taylor, David R Thompson, Melanie J Young, Martin R Ellegaard, M Thomas P Gilbert, Mikkel-Holger S Sinding, George Pacheco, Lara D Shepherd, Alan J D Tennyson, Stefanie Grosser, Emily Kay, Lisa J Nupen, Ursula Ellenberg, David M Houston, Andrew Hart Reeve, Kathryn Johnson, Juan F Masello, Thomas Stracke, Bruce McKinlay, Pablo Garc´ıa Borboroglu, De-Xing Zhang, Guojie Zhang

**Affiliations:** 1 BGI-Shenzhen, Beishan Industrial Zone, Yantian District, Shenzhen 518083, China; 2 State Key Laboratory of Genetic Resources and Evolution, Kunming Institute of Zoology, Chinese Academy of Sciences, Kunming, China; 3 Section for Ecology and Evolution, Department of Biology, University of Copenhagen, DK-2100 Copenhagen, Denmark; 4 Manaaki Whenua Landcare Research, PO Box 69040, Lincoln, Canterbury 7640, New Zealand; 5 Department of Zoology, University of Otago, PO Box 56, Dunedin, Otago 9054, New Zealand; 6 China National Genebank, BGI-Shenzhen, Shenzhen, Guangdong, China; 7 Center for Excellence in Animal Evolution and Genetics, Chinese Academy of Sciences, Kunming 650223, China; 8 Bruce Museum, Greenwich, CT 06830, USA; 9 Department of Zoology, University of Oxford, 11a Mansfield Road, Oxford OX1 3SZ, UK; 10 Department of Biological Sciences, Bowling Green State University, Bowling Green, OH 43403, USA; 11 The Wildlife Hospital Dunedin, School of Veterinary Nursing, Otago Polytechnic, Dunedin, Otago 9016, New Zealand; 12 Copenhagen Zoo, Roskildevej 38, DK-2000 Frederiksberg, Denmark; 13 Department of Veterinary and Animal Sciences, University of Copenhagen, Copenhagen, Denmark; 14 Center for Ecosystem Sentinels, Department of Biology, University of Washington, Seattle, WA 98195, USA; 15 Centre d'Etudes Biologiques de Chize (CEBC), UMR 7372 du CNRS-La Rochelle Universite, 79360 Villiers-en-Bois, France; 16 Research Department, Phillip Island Nature Parks, PO Box 97, Cowes, Phillip Island, Victoria, 3922, Australia; 17 Department of Zoology, University of Oxford, Peter Medawar Building for Pathogen Research, South Parks Road, Oxford OX1 3SY, UK; 18 Hornby Veterinary Centre, 7 Tower Street, Hornby, Christchurch, Canterbury 8042, New Zealand; 19 South Island Wildlife Hospital, Christchurch, Canterbury, New Zealand; 20 National Zoological Garden, South African National Biodiversity Institute, P.O. Box 754, Pretoria 0001, South Africa; 21 Division of Pathology and Laboratory Medicine, University of Western Australia, Crawley, Western Australia 6009, Australia; 22 Institute for Marine and Antarctic Studies, University of Tasmania, Hobart, Tasmania 7001, Australia; 23 Department of Biology, University of Missouri St. Louis, St Louis, MO 63121, USA; 24 British Antarctic Survey, Natural Environment Research Council, High Cross, Cambridge, UK; 25 Justus-Liebig-Universitat Giessen, Heinrich-Buff-Ring 26, 35392 Giessen, Germany; 26 FitzPatrick Institute of African Ornithology, University of Cape Town, Rondebosch 7701, South Africa; 27 Vet Services Hawkes Bay Ltd, 801 Heretaunga Street, Hastings, New Zealand; 28 Wairoa Farm Vets, 77 Queen Street, Wairoa 4108, New Zealand; 29 National Institute of Water and Atmospheric Research Ltd., Private Bag 14901, Kilbirnie, Wellington 6241, New Zealand; 30 Section for Evolutionary Genomics, The GLOBE Institute, Faculty of Health and Medical Sciences, University of Copenhagen, Øster Farimagsgade 5A, Copenhagen, Denmark; 31 NTNU University Museum, Trondheim, Norway; 32 Museum of New Zealand Te Papa Tongarewa, PO Box 467, Wellington 6140, New Zealand; 33 Division of Evolutionary Biology, Faculty of Biology, LMU Munich, Großhaderner Str. 2, 82152 Planegg-Martinsried, Germany; 34 Wildbase, Massey University, Private Bag 11 222, Palmerston North 4442, New Zealand; 35 Wellington Zoo, 200 Daniell St, Newtown, Wellington 6021, New Zealand; 36 National Zoological Gardens of South Africa, Pretoria, South Africa; 37 Department of Ecology, Environment and Evolution, La Trobe University, Melbourne, Victoria, Australia; 38 Global Penguin Society, University of Washington, Seattle, WA, USA; 39 Biodiversity Group, Department of Conservation, Auckland, New Zealand; 40 Department of Biology, Natural History Museum of Denmark, University of Copenhagen, Copenhagen, Denmark; 41 Biodiversity Group, Department of Conservation, Dunedin, New Zealand; 42 Global Penguin Society, Puerto Madryn 9120, Argentina; 43 CESIMAR CCT Cenpat-CONICET, Puerto Madryn 9120, Chubut, Argentina; 44 Center for Computational and Evolutionary Biology, Institute of Zoology, Chinese Academy of Sciences, 1 Beichen West Road, Beijing 100101, China

This is a correction to: GigaScience, Volume 8, Issue 9, September 2019, giz117, https://doi.org/10.1093/gigascience/giz117

In the original version of the article “High-coverage genomes to elucidate the evolution of penguins” by Hailin Pan *et al*. [1], the authors received a request to make some clarifications and changes in the acknowledgements, the following is the modified version:

“We thank the following: John Cockrem, Scott Flemming, Helen McConnell, Chris Rickard, Sarah Fraser, Otto Whitehead, Kyle Morrison, and Amy Van Buren for help collecting samples; Jonathan Banks, Kirsten Rodgers, and Jo Hiscock for sample information; Manuel Paredes Oyarzún and Hernán Rivera Meléndez for facilitating permits and sample collection; Lauren Tworkowski, Richard O'Rorke, and Joanna Sumner for facilitating sample collection; Adrian Smith for providing laboratory support to extract 2 DNA samples; Peter Dearden, Neil Fowke, Michael Knapp, Hoani Langsbury, Claire Porima, Nic Rawlence, Paul Scofield, Jonathan Waters, Janet Wilmshurst, and Jamie Wood for informal discussions regarding the New Zealand indigenous consultation; The New Zealand Department of Conservation for facilitating New Zealand indigenous consultation and approving permits, particularly Neil Fowke and Jesse Mason for facilitating permits and/or obtaining past permit details; Brett Gartrell and Pauline Nijman for providing animal ethics details; and the China National Genebank for contributing the sequencing resources for this project. The Penguin Genome Consortium welcomes participation and collaboration for our ongoing work regarding comparative and evolutionary genomics of penguins.”

## References

[1] Pan H, Cole T L, Bi X et al. High-coverage genomes to elucidate the evolution of penguins. GigaScience. 2019;8(9):1–17. 10.1093/gigascience/giz117 PMC6904868 31531675

