# Supplementary material for: High-coverage genomes to elucidate the evolution of penguins
Source: Gigascience. 2019 Sep 18;8(9):giz117. doi: 10.1093/gigascience/giz117 (PMC6904868; doi:10.1093/gigascience/giz117)
Supplement: giz117_GIGA-D-19-00280_Original_Submission [file giz117_giga-d-19-00280_original_submission.pdf]

GigaScience

High-coverage genomes to elucidate the evolution of penguins

--Manuscript Draft--

Manuscript Number:	GIGA-D-19-00280	
Full Title:	High-coverage genomes to elucidate the evolution of penguins	
Article Type:	Data Note	
Funding Information:	National Key R&D Program of China (2018YFC1406901)	Prof. Guojie Zhang
	Lundeckfonden (R190-2014-2827)	Prof. Guojie Zhang
	Carlsbergfondet (CF CF16-0663)	Prof. Guojie Zhang
	Villum Foundation (25900)	Prof. Guojie Zhang
	Strategic Priority Research Program of the Chinese Academy of Science (XDB13000000)	Prof. Guojie Zhang
	Strategic Priority Research Program of the Chinese Academy of Science (XDB31020000)	Prof. Guojie Zhang
	ERC Consolidator Grant (681396 ‘Extinction Genomics’)	Prof. M. Thomas P. Gilbert
Abstract:	Penguins (Sphenisciformes) are a highly diverse order of seabirds distributed widely across the Southern Hemisphere. They shared a common ancestor with Procellariiformes about 60 million years ago, and have subsequently transited from flying seabirds to flightless marine divers. Approximately 20 extant penguin species are recognised across six well-defined genera, ranging from the Galápagos Islands on the equator, to the oceanic temperate forests of New Zealand, the rocky coastlines of the sub-Antarctic islands, and the sea-ice around Antarctica. To inhabit such diverse and extreme environments, this speciose order has evolved many physiological and morphological adaptations. However, penguins are also highly sensitive to climate change, and most species are already declining or are predicted to decline under future climate change scenarios. Therefore, penguins are an exciting system for understanding the evolutionary processes of speciation, adaptation and demography. Genomic data are an emerging resource for addressing questions about such processes. Here we present a novel dataset of 19 high-coverage genomes that, together with two previously published genomes, encompass all extant penguin species. As such, this dataset provides a novel resource for understanding the evolutionary history within penguins, and between penguins and other avifauna. Against this background, we introduce a major consortium of international scientists dedicated to studying these genomes. Moreover, we highlight emerging issues on ensuring legal and respectful indigenous consultation, particularly for genomic data originating from New Zealand Taonga species. We believe that our dataset and project will be important for cultural heritage and the conservation of this iconic Southern Hemisphere species assemblage.	
Corresponding Author:	Guojie Zhang<br><br>DENMARK	
Corresponding Author Secondary Information:		
Corresponding Author's Institution:		
Corresponding Author's Secondary Institution:		
First Author:	Hailin Pan	
First Author Secondary Information:		

Powered by Editorial Manager® and ProduXion Manager® from Aries Systems Corporation

**Order of Authors:**	Hailin Pan
	Theresa Cole
	Xupeng Bi
	Miaoquan Fang
	Chengran Zhou
	Zhengtao Yang
	Tom Hart
	Juan L. Bouzat
	Lisa S. Argilla
	Mads F. Bertelsen
	P. Dee Boersma
	Charles-André Bost
	Yves Cherel
	Peter Dann
	Steven R. Fiddaman
	Pauline Howard
	Kim Labuschagne
	Thomas Mattern
	Gary Miller
	Patricia Parker
	Richard A. Phillips
	Petra Quillfeldt
	Peter G. Ryan
	Helen Taylor
	David R. Thompson
	Melanie J. Young
	Martin R. Ellegaard
	M. Thomas P. Gilbert
	Mikkel-Holger S. Sinding
	George Pacheco
	Lara D. Shepherd
	Alan J. D. Tennyson
	Stefanie Grosser
	Emily Kay
	Lisa J. Nupen
	Ursula Ellenberg
	David M. Houston
	Andrew Hart Reeve
	Kathryn Johnson
	Juan F. Masello
	Thomas Stracke

Powered by Editorial Manager® and ProduXion Manager® from Aries Systems Corporation

	Bruce McKinlay
	De Xing Zhang
	Guojie Zhang

Order of Authors Secondary Information:	
Additional Information:	
**Question**	**Response**
Are you submitting this manuscript to a special series or article collection?	No
**Experimental design and statistics**<br><br>Full details of the experimental design and statistical methods used should be given in the Methods section, as detailed in our Minimum Standards Reporting Checklist. Information essential to interpreting the data presented should be made available in the figure legends.<br><br>Have you included all the information requested in your manuscript?	Yes
**Resources**<br><br>A description of all resources used, including antibodies, cell lines, animals and software tools, with enough information to allow them to be uniquely identified, should be included in the Methods section. Authors are strongly encouraged to cite Research Resource Identifiers (RRIDs) for antibodies, model organisms and tools, where possible.<br><br>Have you included the information requested as detailed in our Minimum Standards Reporting Checklist?	Yes
**Availability of data and materials**<br><br>All datasets and code on which the conclusions of the paper rely must be either included in your submission or deposited in publicly available repositories (where available and ethically	Yes

Powered by Editorial Manager® and ProduXion Manager® from Aries Systems Corporation

appropriate), referencing such data using a unique identifier in the references and in the "Availability of Data and Materials" section of your manuscript.<br><br>Have you have met the above requirement as detailed in our Minimum Standards Reporting Checklist?	

Powered by Editorial Manager® and ProduXion Manager® from Aries Systems Corporation

Manuscript Click here to download Manuscript Main_File.docx

Gigascience, Data Note

High-coverage genomes to elucidate the evolution of penguins

Hailin Pan[1,2,3,†], Theresa L. Cole[4,5,†], Xupeng Bi[6,7], Miaoquan Fang[6,7], Chengran Zhou[1,6], Zhengtao Yang[1,6], Tom Hart[8], Juan L. Bouzat[9], Lisa S. Argilla[10], Mads F. Bertelsen[11,12], P. Dee Boersma[13], Charles-André Bost[14], Yves Cherel[14], Peter Dann[15], Steven R. Fiddaman[16], Pauline Howard[17,18], Kim Labuschagne[19], Thomas Mattern[5], Gary Miller[20,21], Patricia Parker[22], Richard A. Phillips[23], Petra Quillfeldt[24], Peter G. Ryan[25], Helen Taylor[26,27], David R. Thompson[28], Melanie J. Young[5], Martin R. Ellegaard[29], M. Thomas P. Gilbert[29,30], Mikkel-Holger S. Sinding[29], George Pacheco[29], Lara D. Shepherd[31], Alan J. D. Tennyson[31], Stefanie Grosser[5,32], Emily Kay[33,34], Lisa J. Nupen[35,25], Ursula Ellenberg[36,37], David M. Houston[38], Andrew Hart Reeve[3,39], Kathryn Johnson[34], Juan F. Masello[24], Thomas Stracke[18], Bruce McKinlay[40], De-Xing Zhang[41], Guojie Zhang[1,2,3,7*]

[1]China National Genebank, BGI-Shenzhen, Shenzhen, Guangdong, China.
[2]State Key Laboratory of Genetic Resources and Evolution, Kunming Institute of Zoology, Chinese Academy of Sciences, Kunming, China.
[3]Section for Ecology and Evolution, Department of Biology, University of Copenhagen, DK-2100 Copenhagen, Denmark.
[4]Manaaki Whenua Landcare Research, PO Box 69040, Lincoln, Canterbury 7640, New Zealand.
[5]Department of Zoology, University of Otago, PO Box 56, Dunedin, Otago 9054, New Zealand.
[6]BGI-Shenzhen, Shenzhen 518083, China.
[7]Center for Excellence in Animal Evolution and Genetics, Chinese Academy of Sciences, Kunming 650223, China.
[8]Department of Zoology, University of Oxford, 11a Mansfield Road, South Parks Road, OX1 3SZ, United Kingdom.
[9]Department of Biological Sciences, Bowling Green State University, Bowling Green, OH 43403, United States of America.
[10]The Wildlife Hospital Dunedin, School of Veterinary Nursing, Otago Polytechnic, Dunedin, New Zealand.
[11]Copenhagen Zoo, Roskildevej 38, DK-2000 Frederiksberg, Demark.
[12]Department of Veterinary and Animal Sciences, University of Copenhagen, Denmark.
[13]Center for Ecosystem Sentinels, Department of Biology, University of Washington, Seattle, WA, 98195 United States of America.
[14]Centre d'Etudes Biologiques de Chizé (CEBC), UMR 7372 du CNRS-La Rochelle Université, 79360 Villiers-en-Bois, France.
[15]Research Department, Phillip Island Nature Parks, PO Box 97, Cowes, Phillip Island, VIC 3922, Australia.
[16]Department of Zoology, University of Oxford, Peter Medawar Building for Pathogen Research, South Parks Road, OX1 3SY, United Kingdom.
[17]Hornby Veterinary Centre, 7 Tower Street, Hornby, Christchurch, Canterbury 8042, New Zealand.
[18]South Island Wildlife Hospital, Christchurch, Canterbury, New Zealand.
[19]National Zoological Garden, South African National Biodiversity Institute,,P.O. Box 754, Pretoria, 0001, South Africa.
[20]Division of Pathology and Laboratory Medicine, University of Western Australia, Crawley, Western Australia 6009, Australia.
[21]Institute for Marine and Antarctic Studies, University of Tasmania, Hobart, Tasmania 7001, Australia.
[22]University of Missouri St. Louis, St Louis, MO 63121, United States of America.
[23]British Antarctic Survey, Natural Environment Research Council, High Cross, Cambridge, United Kingdom.
[24]Justus-Liebig-Universität Giessen, Heinrich-Buff-Ring 26, 35392, Giessen, Germany.
[25]FitzPatrick Institute of African Ornithology, University of Cape Town, Rondebosch 7701, South Africa.
[26]Vet Services Hawkes Bay Ltd, 801 Heretaunga Street, Hastings, New Zealand.
[27]Wairoa Farm Vets, 77 Queen Street, Wairoa 4108, New Zealand.
[28]National Institute of Water and Atmospheric Research Ltd., Private Bag 14901, Kilbirnie, Wellington 6241. New Zealand.

[29]Section for Evolutionary Genomics, The GLOBE Institute, Faculty of Health and Medical Sciences, University of Copenhagen, Øster Farimagsgade 5A, Copenhagen, Denmark.
[30]NTNU University Museum, Trondheim, Norway.
[31]Museum of New Zealand Te Papa Tongarewa, PO Box 467, Wellington 6140, New Zealand.
[32]Division of Evolutionary Biology, Faculty of Biology, LMU Munich, Großhaderner Str. 2, 82152 Planegg-Martinsried, Germany.
[33]Wildbase, Massey University, Private Bag 11 222, Palmerston North 4442, New Zealand.
[34]Wellington Zoo, 200 Daniell St, Newtown, Wellington 6021, New Zealand.
[35]National Zoological Gardens of South Africa, Pretoria, South Africa.
[36]Department of Ecology, Environment and Evolution, La Trobe University, Melbourne, Victoria, Australia.
[37]Global Penguin Society, University of Washington, Seattle, United States of America.
[38]Biodiversity Group, Department of Conservation, Auckland, New Zealand.
[39]Department of Biology, Natural History Museum of Denmark, University of Copenhagen, Copenhagen, Denmark.
[40]Biodiversity Group, Department of Conservation, Dunedin, New Zealand.
[41]State Key Laboratory of Integrated Management of Pest Insects and Rodents, Institute of Zoology, Chinese Academy of Sciences, 1 Beichen West Road, Beijing 100101, China.

[†]HP and TLC contributed equally to this work

[*]Corresponding author
<Guojie.Zhang@bio.ku.dk>

Abstract

Penguins (Sphenisciformes) are a highly diverse order of seabirds distributed widely across the Southern Hemisphere. They shared a common ancestor with Procellariiformes about 60 million years ago, and have subsequently transited from flying seabirds to flightless marine divers. Approximately 20 extant penguin species are recognised across six well-defined genera, ranging from the Galápagos Islands on the equator, to the oceanic temperate forests of New Zealand, the rocky coastlines of the sub-Antarctic islands, and the sea-ice around Antarctica. To inhabit such diverse and extreme environments, this speciose order has evolved many physiological and morphological adaptations. However, penguins are also highly sensitive to climate change, and most species are already declining or are predicted to decline under future climate change scenarios. Therefore, penguins are an exciting system for understanding the evolutionary processes of speciation, adaptation and demography. Genomic data are an emerging resource for addressing questions about such processes. Here we present a novel dataset of 19 high-coverage genomes that, together with two previously published genomes, encompass all extant penguin species. As such, this dataset provides a novel resource for understanding the evolutionary history within penguins, and between penguins and other avifauna. Against this background, we introduce a major consortium of international scientists dedicated to studying these genomes. Moreover, we highlight emerging issues on ensuring legal and respectful indigenous consultation, particularly for genomic data originating from New Zealand Taonga species. We believe that our dataset and project will be important for cultural heritage and the conservation of this iconic Southern Hemisphere species assemblage.

Key words

Genomics, Sphenisciformes, Comparative evolution, Phylogenetics, Speciation, Biogeography, Demography, Climate change, Antarctica, Evolution

Data Description

Context

Penguins (Sphenisciformes) are a highly diverse order of seabirds distributed widely across the Southern Hemisphere (Figure 1). Approximately 20 extant penguin species are recognised across six well-defined genera (*Aptenodytes*, *Pygoscelis*, *Eudyptula*, *Spheniscus*, *Eudyptes* and *Megadyptes*; [1-3]), with some debate about the species/lineage boundaries between New Zealand little blue, New Zealand white-flippered and Australian fairy penguins (*Eudyptula minor minor*, *E. m. albosignata, E. novaehollandiae*) [4-6), northern rockhopper, western rockhopper and eastern rockhopper penguins (*Eudyptes moseleyi*, *E. chrysocome, E filholi* [3, 7-8]), Fiordland crested and Snares crested penguins (*E. pachyrhynchus*, *E. robustus* [9-10]) and macaroni and royal penguins (*E. chrysolophus chrysolophus*, *E. c. schlegeli* [3, 8, 11]). The group also has an extensive fossil record, with more than 50 extinct penguins documented to date (3, 12-13), extending back more than 60 million years (12). In contrast to the many fossil penguins that were giants (approximately 101 kilograms [kg] [13]), extant penguins span a more modest range of sizes (14-15), with the emperor penguin (*Aptenodytes forsteri*) the largest (30 kg) and *Eudyptula* penguins the smallest (1 kg).

The radiation of crown-group penguins provides an excellent case study for researching biogeographic impacts on speciation processes. Penguins inhabit every major coastline in the Southern Hemisphere, and almost every island archipelago in the Southern Ocean (16. Their range extends to unique ecological niches, from the tropical Galápagos Islands (Galápagos penguin; *Spheniscus mendiculus*), to the oceanic temperate forests of New Zealand (*Eudyptes pachyrhynchus*), rocky coastlines of the sub-Antarctic islands (*E. filholi*) and the sea-ice around

Antarctica (*Aptenodytes forsteri*) (17). For this reason, penguins have evolved many unique adaptations, specific to the variety of ecological environments. Previous studies have suggested that global climate change during the Eocene (18-19), substantial oceanographic currents (7) and geological island uplift (3) were key drivers of penguin diversification. Although the phylogenetic relationships within penguins are relatively well understood (1, 3, 20), it remains uncertain whether *Aptenodytes* and/or *Pygoscelis*, which are both endemic to coastal Antarctica and Antarctic and subantarctic islands, are sister taxa to all other extant penguins (3), many of which are endemic to isolated Southern Ocean islands. Understanding the evolutionary diversification of penguins in respect to geological and climatic changes remains a substantial gap in understanding the biogeographic history of these iconic birds.

Although penguins are tied to landmasses to breed (21), all spend most of their lives at sea (22), and are therefore important components of terrestrial, coastal and marine ecosystems (23). While some taxa inhabit environments with strong winds and extreme cold temperatures, experiencing seasonal fluctuations in the length of daylight across the breeding and chick-rearing seasons (24), others inhabit relatively temperate, or even tropical climates, with little variation in day length. The unique morphological and physiological adaptations that have evolved within penguins include the complete loss of aerial flight, where penguins instead employ their flipper-like wings in wing-propelled diving (25), densely-packed waterproof and insulating feathers (26-27), visual sensitivity of the eye lens for underwater predation (28-30), dense bones, stiff wing joints and reduced distal wing musculature to overcome buoyancy in water (31-33), enhanced thermoregulation for extreme low temperatures, long-term fasting, ability to digest secreted food, delayed digestion (34-40), different plumage and crest ornaments (41) and catastrophic moult (42). As such, penguins are an excellent system to study comparative evolution of adaptive traits.

Penguins are also sentinels of the Southern Ocean (16), being particularly sensitive to human and environmental change (43-44). Extensive demographic monitoring programs have indicated that many penguin species are declining in response to global warming (43-45), pollution, environmental degradation and competition with fisheries, which are considered key drivers of these population declines (46-49). Demographic coalescent models have demonstrated dramatic population declines during the Pleistocene ice ages, followed by rapid population expansions in response to global warming (50-53). Future global warming is predicted to cause significant population declines (43; 54-56). Understanding past demographic histories and inferring future demographic trajectories therefore remains important steps for predicting ecosystem-wide changes in this rapidly warming part of the planet.

Although penguins are a relatively well-studied seabird assemblage, previous evolutionary studies have been limited by the genetic markers employed, such as short mitochondrial (2, 10, 57-59) or nuclear sequences (1, 8, 60-61), microsatellites (62-63), partial mitochondrial genomes (3, 64), or single nucleotide polymorphisms (11, 52-53, 65-67). Several studies have hinted at associations between biological patterns and climate change (50-53, 59, 68). Only a few studies have explored genome-wide evolutionary processes among penguins (50, 69), or between penguins and other birds (70-72) and these studies have focussed on just two Antarctic taxa: the Adélie penguin (*P. adeliae*) and *Aptenodytes forsteri*. However, these studies have created a basic framework to understand the timing of penguin diversification, identify population fluctuations during past climate cycles, and have hinted at the molecular basis for a range of physiological and morphological adaptations (50). The molecular genomic basis for the unique morphological and physiological adaptations of penguins, compared to other aquatic

and terrestrial avifauna, remains largely unknown. No previous study has attempted to explore the evolution of all penguins under a comparative genomic or evolutionary framework. In this Data Note, we present 19 new high-quality genomes that, together with the two genomes obtained by (50), encompass all extant penguin taxa. These data provide a critical resource for understanding the drivers of penguin evolution, the molecular basis of morphological and physiological adaptations, and demographics. For species naming, we follow standard nomenclature, however for *Eudyptula* we follow (5, 73) and for *Eudyptes* and *Megadyptes* we follow (3).

Methods

Sample collection, library construction, and sequencing

While it is possible to recover genome sequences from historical museum samples (74), such genomes are often low quality and/or fragmented (75), limiting the ability of downstream analyses. Our project design (see below) relies on high-coverage genomes with little missing data (see [50]). Therefore, we designed our sample collection to include only high-quality blood samples. We collected 94 blood samples spanning 19 different penguin species (1 – 28 samples per species; Supplementary Table 1). Samples were derived from the wild, zoological parks or wildlife hospitals, and were obtained according to strict permitting procedures, animal ethics and consultation with indigenous representatives (Supplementary Table 1).

DNA was extracted from each sample at one of three laboratories as follows: we used the HiPire Blood DNA Midi Kit II at BGI (Hong Kong), the Qiagen DNeasy Blood and Tissue Kit (Qiagen, Valencia–CA, USA) at the University of Oxford (United Kingdom) and the KingFisher Cell and Tissue Kit in combination with the KingFisher Duo Prime Purification

System the University of Copenhagen (Denmark). All downstream methods were conducted at BGI. We diluted each DNA extraction to 20 μl using TE buffer. The quality and quantity of each DNA extraction was assessed by first estimating the concentration of 1 μl DNA extraction on a Microplate Reader and DNA fragment size was evaluated by pulse gel electrophoresis or on a 1% agarose gel electrophoresis. Following quality control, a single sample per species was chosen for genomic library construction (Table 1).

We constructed one or more genomic libraries for each of the 19 penguin species. We constructed 10X genomic libraries for each species with DNA fragments longer than 40 Kbp. To do this, we attached a specific unique barcode to one end of short DNA fragments which are broken from one long DNA fragment, using standard protocols provided by Chromium™ Genome Solution. As this protocol encompasses >1 million specific barcodes in a single solution, it decreases the chance of short DNA fragments with the same barcode being derived from unrelated long DNA fragments. For those species with shorter DNA fragments (<40 Kbp), we constructed genomic libraries following Illumina (San Diego, CA) or BGIseq 500 (76) protocols. Those protocols resulted in several paired-end libraries with insert sizes either 250 bp or 500 bp, in addition to several mate-pair libraries with insert sizes ranging from 2 kbp – 20 Kbp. Following library construction, those using Illumina protocols were sequenced on a HiSeq X ten or a HiSeq 4000 platform (77), and those using BGIseq 500 and 10X genomic protocols were sequenced on a BGIseq 500 platform (78) (Table 2). Following sequencing, we generated 3.24 Tb sequencing reads encompassing all 19 penguin species, obtaining >111 Gb data per species (Table 2).

Genome assembly and quality evaluation

Sequences obtained from the 250 bp insert size libraries and the 10X libraries were used to evaluate the genome size for each penguin using a k-mer approach (79). Reads were scanned using a 17 bp window with 1 bp sliding and the frequency of each 17 k-mer was recorded. After scanning all the reads, the k-mer frequency distributions were plotted and the depth with the highest frequency (K_dep) was defined. The genome size was estimated as the read number * (read length – 17 + 1)/K_dep.

Sequencing errors have a major impact on subsequent genome assembly, as they introduce both mistakes in the assembly and also decrease the assembly continuities. Several features can be linked to sequencing noises, including low quality bases, adaptor contamination and duplication (80). To remove the potential biases introduced by sequencing noises, we filtered our raw sequencing reads prior to genome assembly, following strict standards including: 1) discarding paired-end reads containing overlaps; 2) removing reads with >20% low quality bases as the quality score was smaller than 10; 3) removing reads with >5% ambiguous N bases; 4) removing paired-end reads containing identical sequences likely to be Polymerase Chain Reaction duplicates; and 5) removing reads with adaptor sequences. Following filtering, each genome contained >104 Gb data. Overall, we obtained a total of 2.56 Tb high-quality data for all 19 penguin genomes (Table 2).

Both SOAPdenovo v. 2-2.04 (81) and Allpaths-lg (82) were used to assemble the genomic libraries from the various insert sizes. For SOAPdenovo, paired-end reads from small insert size libraries were used to construct de Bruijn graphs, with various k-mer ranging from 23 – 47. Contigs were subsequently constructed using contig modular with the “-D 1 -g” parameter to remove edges containing coverages no larger than 1. Following this, “map -k 35 -g” was used to map mate-pair reads into contigs, with k-mer size 35. Finally, we conducted scaffolding

with parameters “scaff –g –F” to assemble the contigs into longer linkages. The best version, in terms of various k-mer in the graph construction step was chosen as the SOAPdenovo representative for each species. In addition, we also assembled genomic libraries from various insert sizes using Allpaths-lg following the default parameters. By comparing the assemblies from both SOAPdenovo and Allpaths-lg, according to both the scaffold N50 and the total length, we chose the best assembler as a representative for each of the 19 penguin species. Supernova v. 2.0 (83), recommended for 10X genomic data (83), was used to assemble those species with 10X genomic libraries, following the default parameters. The optimal assembly strategy chosen for each penguin species is listed in Supplementary Table 2. For each assembly, we used GapCloser v. 1.12 (81) to locally assemble and close gaps within each scaffold following the default parameters.

All penguins (including those obtained in [50]) were estimated to have approximately a 1.3 Gb genome (Figure 2), containing little variances. Most assemblies have both a longer scaffold N50 and contig N50 than the *Aptenodytes forsteri* and *Pygoscelis adeliae* assemblies obtained in (50) (Figure 2). In total, the 21 genomes contained a scaffold N50 >1 Mb, and of those, 13 genomes contained a scaffold N50 >3 Mb. All penguin genomes contain a contig N50 >19 kb and 15 of the genomes are >30 kb. The maximum contig N50 extends to 163 kb for the macaroni penguin (*Eudyptes chrysolophus chrysolophus*) (Figure 2). The highest-quality genome is *Eudyptula novaehollandiae* encompassing a 29.3 Mb scaffold N50. Therefore, our results demonstrate consistency and high-quality among all 21 penguin genomes (Figure 2).

The genome assembly completeness provides an evaluation of the assembly quality. We used Benchmarking Universal Single-Copy Orthologs v. 3.0.2 (BUSCO) (84) to evaluate our newly assembled penguin genomes with the avian database aves_odb9 database (84) which

encompasses 4,915 conserved avian orthologs. Only about 3% of the core genes in aves_odb9 could not be annotated on the 21 penguin genomes (ranging between 2% – 7.8%). This demonstrates that all 21 penguin genomes are near-complete, containing only a few gaps. We identified an average of 90% complete core genes on each of the 21 penguin genomes, with the richest being 93.8% on *Eudyptes chrysocome*. Furthermore, when several genes were annotated in more than one copy, we considered them as duplications. Duplication rates among the 21 penguin genomes varied only between 0.6% – 8.6%. In addition, only about 4% of the core genes were partly annotated on each of the 21 penguin genomes (Figure 2). Overall, we obtained almost-complete, high-quality genomes. Our genomic dataset (including those obtained in [50]) encompass all extant penguin species, representing a comprehensive dataset.

Repeat annotation

We used RepeatMasker v. 4.0.7 (85) <http://www.repeatmasker.org>, TRF v. 4.09 (86) and RepeatModeler v. 1.0.8 (87) <http://www.repeatmasker.org> to identify repetitive sequences in each of the penguin genomes. We compared our genomes to five avian outgroups; wedge-rumped storm petrel (*Hydrobates tethys*), Wilson's storm petrel (*Oceanites oceanicus*), Atlantic yellow-nosed albatross (*Thalassarche chlororhynchos*), zebra finch (*Taeniopygia guttata*) and chicken (*Gallus gallus*). Genome sequences were aligned to RepBase23.04 (88) through RepeatMasker, and each hit was further classified into detailed categories. Tandem repeats, which are a series of DNA sequences containing >2 adjacent copies were identified using TRF using the default parameters. In addition, we used RepeatModeler in a *de novo* repeat family identifying approach. All identified repeat elements were classified into seven categories (DNA, LINE, SINE, LTR, Other, Unknown, TRF) according to classification in repeat databases. Repeat annotations using the three methods were combined into a non-redundant repeat annotation for each penguin genome and the five outgroups.

About 10% of the genome sequences were identified as repeat elements on each penguin genome, which is similar to the five outgroups (Table 2). Although all penguin genomes had similar repeat content, they varied in content for each category. In all penguins and outgroups, the most abundant repeat category was LINE. *Eudyptes moseleyi* has the richest TRF of 3.52%, which is substantially greater than *Aptenodytes forsteri*, which has a TRF of 2.24% and contains the second richest TRF repeat in all penguins. *Eudyptula minor minor* had the most genome sequences identified as LTR (4.26%). See Table 3 for specific details on repeat annotations for each species.

Protein coding gene annotation

We used the annotation methods developed by The Bird 10,000 Genomes (B10K) consortium <http://b10k.genomics.cn/> to annotate the 21 penguin genomes. Prior to annotating the protein coding genes, a non-redundant avian reference gene set, consisting of protein sequences from *Taeniopygia guttata* and *Gallus gallus* was generated (see [70]). Whole genome protein sequences of Ensembl gene sets (release-85) of *Taeniopygia guttata* and *Gallus gallus* were then used to identify 12,337 orthologs based on whole genome synteny relationships that were downloaded from the UCSC Genome Browser <ftp://hgdownload.soe.ucsc.edu/goldenPath/galGal4/vsTaeGut2/>. For both *Taeniopygia guttata* and *Gallus gallus*, we compared the two proteins in each ortholog, and chose the longer homologous sequence with the human ortholog protein sequence in the reference gene set. Within 12,337 orthologs, 6,888 from *Taeniopygia guttata* and 5,449 from *Gallus gallus* were selected as the reference gene set. Following this, specific genes of *Taeniopygia guttata* or *Gallus gallus* were added to the reference gene set. This reference gene set comprised of 5,084 *Taeniopygia guttata* genes without *Gallus gallus* orthologs, and 3,158 *G. gallus* genes that had

not been identified as ortholog genes to *Taeniopygia guttata*. Finally, protein sequences were filtered if they contained <50 amino acids, consisted of function as transposons/retrotransposons, or contained only a single non-functional exon. The final avian reference gene set therefore contained 20,181 protein coding genes.

To annotate the protein coding genes from the penguin genomes, protein sequences from the avian reference gene set were then mapped to each of the 21 penguin genomes. First, protein sequences were aligned to each penguin genome using tblastn v. 2.2.2 (89) with a 1e-5 e-value cut-off. Multiple adjacent hits from the same protein were then linked together using genBlastA v. 1.0.4 (90) to obtain the candidate gene boundary. A candidate hit was removed if a protein had <30% amino acids aligned to the penguin genome. For each candidate hit for each protein, we extracted genomic sequences covering this hit with 2 kbp upstream and downstream of the extension. Extracted genome sequences and corresponding homologous protein sequences were then prepared as input for GeneWise v. 2.4.1 (91) to the annotated protein coding gene models, which included exon and intron boundaries. Coding sequences for each annotated gene model was extracted from each genome according to the annotated gene model, and then each coding sequence was translated into the protein sequence. This annotated protein sequence was then aligned with the corresponding homolog protein sequence using MUSCLE v. 3.8.31 (92), while removing annotated proteins with <40% identity with the corresponding homolog protein sequence. Annotated proteins with <30 amino acids and annotated proteins containing >2 frame shifts or one premature stop codon were then removed. If a genome locus had been annotated using several gene models, the gene model with the highest identity with the corresponding homolog protein was selected. Therefore, the annotated gene set for our penguin genomes contained no overlapping genes.

Protein sequences from human (hg38) and avian transcripts were also mapped to each penguin genome and the annotated gene models (as above). For the avian transcripts dataset, we obtained 71 avian transcriptomic samples from NCBI (93) (Supplementary Table 3), and assembled those into transcripts using either Newbler v2.9 (94) for 454 sequencing assemblies or Trinity v20140717 (95) for Illumina sequencing assemblies. We used ORFfinder (93) to identify open reading frames (ORF) for transcripts, and the protein sequences were then translated from the ORF. The protein sequences translated from the transcripts were then mapped to the avian reference gene set and the human protein sequences, while removing those with similarity to the avian reference gene set or the human protein sequences. Transcripts with ORF length <150 bp were also removed. Protein sequences from 5,257 transcripts were then used for annotation. Three gene model sets annotated from the avian reference gene set, the human protein sequences and transcriptome were then combined into a final non-redundant gene set. We prioritized three gene model sets in the following order: avian reference gene set > human protein > transcriptome.

After applying the above methods, we annotated the 19 newly assembled penguin genomes, as well as the two previously published penguin genomes (50). We identified about 16,000 genes on each penguin genome, which is similar to the genomes of *Taeniopygia guttata* and *Gallus gallus*. The average gene length and coding sequence length are approximately 19 kbp and 1.3 kbp, respectively. Each gene encompasses approximately eight exons, with an average length of 170 bp. Intron lengths are an average length of 2.6 kbp (Table 4).

Gene function annotation

To assign functions to each gene, we aligned each gene to three functional databases: Swiss-Prot release-2019_03 (96), InterPro v. 68.0 (97) and KEGG v89.1 (98). Protein sequences of

each gene were aligned to Swiss-Prot database using BLASTP (89) and the function of the best hit was selected as the function annotation for this gene. We then searched InterPro databases which encompass ProDom, PRINTS, Pfam, SMART, PANTHER, ProSiteProfiles and ProSitePatterns to obtain the motifs and domains for each gene. Gene Ontology (99) terms for each gene were obtained from the corresponding InterPro entry. To identify the pathways in which the gene might be involved, protein sequences for each gene were then aligned against the KEGG database using BLASTP. For each penguin genome, a total of >99% of the protein coding genes were assigned at least one function annotation in each penguin, which is similar to the five outgroups (Table 5). Overall, >95% of the protein genes were assigned a Swiss-Prot function, demonstrating high-quality gene sets.

Re-use Potential

Consortium organization and further research plans

The 19 high-coverage genomes presented here, along with the *Aptenodytes forsteri* and *Pygoscelis adeliae* genomes presented by members of our consortium in 2014 (50), provide an exciting resource for understanding evolutionary diversification, the molecular basis for unique functional adaptation, and demographic histories of penguins. The Penguin Genome Consortium is an international team of scientists with backgrounds in marine ornithology, ecology, molecular biology, evolutionary and comparative genomics, physiology, palaeontology, veterinary science and bioinformatics. The diverse skills encompassed within our highly-collaborative consortium will be essential to study these genomes under comparative genomic and evolutionary frameworks. In doing so, we will expand on (50) by investigating three key areas related to penguin evolution and adaptation.

Evolutionary relationships and taxonomic boundaries

With a deep evolutionary history, and diverse radiation, penguins provide an exciting system to understand the evolutionary drivers of diversification (3). Moreover, robust taxonomic frameworks can be crucial for directing limited conservation resources for maximum gains. Significant uncertainty remains regarding species/lineage boundaries between some closely related penguin taxa. The genomes generated here therefore provide an exciting new dataset to examine taxonomic and phylogenomic patterns for understanding penguin evolution.

Comparative genomics and adaptation

Penguins provide an excellent system to study comparative evolutionary adaptation (50). We shall use our genomes to explore comparative evolution among penguins, and between penguins and other avian orders. By examining loci under positive selection, we shall reveal the molecular basis for the unique physiological and morphological adaptations to different environments and ecologies that are exhibited by penguins.

Penguins in a changing world

Penguins are sensitive indicators of environmental change (43-44). It is predicted that future climate change will lead to significant declines in many penguin populations (46-49). Conservation management decisions can be guided by demographic assessments. However, there remains a substantial gap in predicting ecosystem-wide changes to future climate change. As such, demographic analyses of these genomes will be critical for conservation management of penguins and other Southern Ocean assemblages.

Cultural significance

The context in which wildlife research in New Zealand is undertaken is evolving rapidly and

heading into new legal and novel cultural contexts (101-103). Recent initiatives such as the bestowing of the rights of an individual on Te Urewera, a former National Park, set an international precedent for this change in approach (104). Therefore, it is critical that research permissions are obtained and appropriate indigenous consultation with Iwi, Whānau and Hapū is conducted. The regulatory arm of the Government in this process, the Department of Conservation, is legally required to give effect to the Principles of the Treaty of Waitangi <http://www.waitangitribunal.govt.nz/> in its administration of the legislation pursuant to which Authorities are issues.

At another level the Ngāi Tahu Deed of Settlement Act recognises all penguin species as Taonga or treasured possessions (105). Consequently, not only is it a legal requirement to undertake rigorous Māori consultation when studying Taonga (106-107), the Department of Conservation has to have particular regard to the views of Iwi, Whānau or Hapū when considering whether to authorise any application. Recent discussions have also emphasised that Taonga genomes are sacred (tapu), as they are considered to contain both the living and the future generations (whakapapa, mauri and wairua of tipuna), with Māori concerns surrounding the commercialisation, ownership, storage and modification of Taonga genomes (108). We generated Taonga genomes encompassing hoiho (yellow-eyed penguin, *Megadyptes antipodes antipodes*), kororā (little penguin, *Eudyptula* spp.), pokotiwha (Snares-crested penguin; *Eudyptes robustus*), tawaki (Fiordland-crested penguin; *Eudyptes pachyrhynchus*) and erect-crested penguin (*Eudyptes sclateri*). These genomes were obtained following rigorous Department of Conservation permitting procedures (including collection, holding and exporting permits) and following Department of Conservation Iwi, Whānau or Hapū consultation (Supplementary Table 1). Several of the Taonga genomes studied here were collected alongside broader research projects, and additional consultation efforts were

undertaken for those projects. We emphasise that there will be no commercialisation, ownership or modification of any of the genomes presented here. While these Taonga genomes will be publically available, it is critical that new researchers studying these genomes take the appropriate steps to seek additional Māori permissions and consultation, which will ensure respect of New Zealand cultural values.

The emerging issues surrounding the generation and use of Taonga genomes also highlight that Māori consultation should also be undertaken when obtaining genomes from Taonga housed in overseas museum collections. We hope that the data and our research questions presented here, and our future research outputs using these genomes will be valuable for both cultural heritage and for conservation management of penguin populations.

Early-release use of the data

The Fort Lauderdale (109) and Toronto (110) declarations state that in exchange for early-release of datasets, the data producers retain the right to be the first to describe and analyse the complete datasets in peer-reviewed publications. Comparative and evolutionary genomic analyses are currently being carried out, and the consortium welcomes new members interested in contributing to this work. While this work is still underway we have published these 19 penguin genomes to provide early-release, while requesting researchers intending to use this data for similar cross-species comparisons to continue to follow the Fort Lauderdale and Toronto rules.

Conclusions

Genomics is prohibitively costly, requires high-quality samples and extensive laboratory and bioinformatic skills. The genomics era has been boosted by global research consortiums, which

bring together contextual, technical and analytical skills spanning a network of international collaborations (111-114). Our consortium and dataset introduced here is no exception, and as such, we expect our future research using these genomes to bring together additional collaborators that encompass a wide range of expertise regarding penguin biology and physiology. At another level, collecting high-quality fresh blood samples from some of the most remote regions in the Southern Ocean remains technically and logistically difficult, requiring the efforts and long-term organisation from many collaborations and expedition programs. While this study is an exciting development for understanding the evolution of penguins, the global efforts involved in designing our study, obtaining samples and developing appropriate sequencing and bioinformatic pipelines have been extensive. The dataset and project design introduced here highlights the need for transparent research projects and global collaborations, which together maximise the use of samples, minimising sequencing costs, and laboratory and analytical efforts.

In this study we have presented 19 new high-coverage penguin genomes. Together with two genomes previously obtained by members of our consortium in (50), this combined dataset encompasses the genomes of all extant penguin species. We will use this dataset to address a range of evolutionary, adaptive, biogeographic and demographic questions regarding penguins. As such, we not only hope that our ongoing projects which encompass these genomes will provide novel insights for understanding the broad evolution and adaptation of avifauna to different environments, but also that this knowledge will increase cultural heritage and aid conservation management decisions for remote Southern Ocean regions.

Availability of supporting data

The genome sequencing data and assemblies of this study have been deposited in the CNSA (https://db.cngb.org/cnsa/) of the CNGBdb database with the accession number CNP0000605, as well as the NCBI database with the Bioproject ID PRJNA556735 (*Aptenodytes patagonicus*: SAMN12384866; SAMN12384872; *Eudyptes chrysolophus chrysolophus*: SAMN12384869; *E. c. schlegeli*: SAMN12384870; *E. chrysocome*: *E. filholi*: SAMN12384873; *E. moseleyi*: SAMN12384871; *E. pachyrhynchus*: SAMN12384875; *Eudyptes robustus*: SAMN12384876; *E. sclateri*: SAMN12384874; *Eudyptula minor albosignata*: SAMN12384880; *E. m. minor*: SAMN12384879; *E. novaehollandiae*: SAMN12384878; *Megadyptes antipodes antipodes*: SAMN12384877; *Pygoscelis antarctica*: SAMN12384868; *P. papua*: SAMN12384867; *Spheniscus demersus*: SAMN12384881; *S. humboldti*: SAMN12384883; *S. magellanicus*: SAMN12384882; *S. mendiculus*: SAMN12384884.

Declarations

Ethics approval and consent to participate

All samples were obtained under valid animal ethics permits.

Competing interests

The authors declare that they have no competing interests.

Funding

This project was supported by the National Key R&D Program of China (MOST) grant 2018YFC1406901. TLC was supported by an Otago University postgraduate publishing bursary. GZ was supported by the Lundbeckfonden (grant No. R190-2014-2827), Carlsbergfondet (grant No. CF CF16-0663), the Villum Foundation (grant No. 25900) and by

the Strategic Priority Research Program of the Chinese Academy of Science (grant No. XDB13000000, XDB31020000). MTPG was supported by the ERC Consolidator Grant 681396 'Extinction Genomics'.

Authors' contributions

GZ developed the concept; GZ, TLC and HP designed the project; GZ, TLC and HP wrote the manuscript; LSA, JLB, MFB, PDB, TLC, YC, PD, UE, SRF, SG, DMH, PH, TH, EK, KL, GM, TM, LJN, PP, PGR, DRT, HT and MJY collected and/or provided; JLB, TLC, AHR, TH, KJ, BM, TS, DRT and GZ facilitated sample collection; HP, SRF, MRE, M-HSS and GP undertook laboratory work. HP, XB, MF, CZ, ZY undertook the bioinformatics work; GZ, TLC, HP, C-AB, MRE, MTPG, TH JFM, RAP, AJDT, LDS, M-HSS and PQ helped design sampling directions. All authors contributed to the final manuscript.

Acknowledgements

We thank the following: John Cockrem, Scott Flemming, Helen McConnell, Chris Rickard, Sarah Fraser, Otto Whitehead, Kyle Morrison and Amy Van Buren for help collecting samples; Jonathan Banks, Kirsten Rodgers and Jo Hiscock for sample information; Manuel Paredes Oyarzún and Hernán Rivera Meléndez for facilitating permits and sample collection; Lauren Tworkowski, Richard O'Rorke and Joanna Sumner for facilitating sample collection, Adrian Smith for providing laboratory support to extract two DNA samples; Peter Dearden, Neil Fowke, Michael Knapp, Claire Porima, Paul Scofield, Ben Te Aika, Jonathan Waters, Janet Wilmshurst and Jamie Wood for discussions regarding New Zealand indigenous consultation; Neil Fowke and Jesse Mason for facilitating New Zealand Department of Conservation permits and/or obtaining past permit details; and the China National Genebank for contributing the sequencing resources for this project. The Penguin Genome Consortium welcomes

participation and collaboration for our ongoing work regarding comparative and evolutionary genomics of penguins.

References

1. Ksepka DT, Bertelli S & Giannini NP. The phylogeny of the living and fossil Sphenisciformes (penguins). Cladistics. 2006; 22(5):412–441.

2. Cole TL, Waters J, Shepherd LD, et al. Ancient DNA reveals that the 'extinct' Hunter Island penguin (*Tasidyptes hunteri*) is not a distinct taxon. Zool J Linn Soc-Lond. 2018; 182(2):459–464.

3. Cole TL, Ksepka DT, Mitchell KJ, et al. Mitogenomes uncover extinct penguin taxa and reveal island formation as a key driver of speciation. Mol Biol Evol. 2019; 36(4):784–797.

4. Challies CW & Burleigh RR. Abundance and breeding distribution of the white-flippered penguin (*Eudyptula minor albosignata*) on Banks Peninsula, New Zealand. Notornis. 2004; 51(1):1–6.

5. Grosser S, Rawlence NJ, Anderson CNK, et al. Invader or resident? Ancient-DNA reveals rapid species turnover in New Zealand little penguins. P Roy Soc B-Biol Sci. 2016; 283(1824):20152879.

6. Mattern T & Wilson K-J. New Zealand penguins – current knowledge and research priorities. A report compiled for Birds New Zealand. 2018; July.

7. Banks J, Van Buren A, Cherel Y, et al. Genetic evidence for three species of rockhopper penguins, *Eudyptes chrycosome*. Polar Biol. 2006; 30(1):61–67.

8. Frugone M-J, Lowther A, Noll D, et al. Contrasting phylogeographic pattern among *Eudyptes* penguins around the Southern Ocean. Sci Rep-UK. 2018; 8(1):17481.

9. Christidis L, Boles WE. Systematics and Taxonomy of Australian Birds. Canberra: CSIRO Publishing. 2008; pp 98.

10. Cole TL, Rawlence NJ, Dussex N, et al. Ancient DNA of crested penguins: Testing for temporal genetic shifts in the world's most diverse penguin clade. Mol Phylogenet Evol. 2019; 131:72–79.

11. Frugone M-J, López ME, Segovia NI, et al. More than the eye can see: Genomic insights into the drivers of genetic differentiation in Royal/Macaroni penguins across the Southern Ocean. Mol Phylogenet Evol. 2019; 106563.

12. Slack KE, Jones CM, Ando T, et al. Early penguin fossils, plus mitochondrial genomes, calibrate avian evolution. Mol Biol Evol. 2006; 23(6):1144–1155.

13. Mayr G, Scofield RP, De Pietri VL, et al. A Paleocene penguin from New Zealand substantiates multiple origins of gigantism in fossil Sphenisciformes. Nat Commun. 2017; 8(1):1927.

14. Stonehouse B. The general biology and thermal balances of penguins. In Adv Ecol Res. 1967; pp. 131–196.

15. Marchant S, Higgins PJ. (1990). Handbook of Australian, New Zealand and Antarctic birds. Vol. 1, Pt. B. Oxford University Press, Melbourne.

16. Boersma PD. Penguins as marine sentinels. BioScience. 2008; 58(7):597–607.

17. Ropert-Coudert Y, Hindell MA, Phillips R, et al. Biogeographic patterns of birds and mammals. In. The Biogeographic Atlas of the Southern Ocean. Scientific Committee on Antarctic Research. (2014):pp. 364–387.

18. Baker AJ, Pereira SL, Haddrath OP, et al. Multiple gene evidence for expansion of extant penguins out of Antarctica due to global cooling. P Roy Soc B-Biol Sci. 2006; 273(1582):11–17.

19. Acosta Hospitaleche C, Reguero M, Scarano A. Main pathways in the evolution of the Paleogene Antarctic Sphenisciformes. J S Am Earth Sci. 2013; 43:101–111.

20. Bertelli S & Giannini NP. A phylogeny of extant penguins (Aves: Sphenisciformes) combining morphology and mitochondrial sequences. Cladistics. 2005; 21(3):209–239.

21. Garcia Borboroglu P, Boersma PD. Penguins: Natural History and Conservation. University of Washington Press. Seattle, USA. 2013; pp 328.

22. Thiébot JB, Cherel Y, Trathan PN, et al. Coexistence of oceanic predators on wintering areas explained by population-scale foraging segregation in space or time. Ecology. 2012; 93(1):12–130.

23. Woehler EJ, Cooper J, Croxall JP, et al. A statistical assessment of the status and trends of Antarctic and sub-Antarctic seabirds. Scientific Committee on Antarctic Research, Cambridge, UK. 2011.

24. Goldsmith R, Sladen WJ. Temperature regulation of some Antarctic penguins. J Physiol. 1961;157:251–262.

25. Ksepka DT, Ando T. Penguins past, present, and future: trends in the evolution of the Sphenisciformes. In: Dyke G, Kaiser G, editors. Living Dinosaurs. Oxford: Wiley; 2011. pp. 155–186.

26. Watson M. Report on the Anatomy of the Spheniscidae Collected by HMS Challenger, During the Years 1873–1876. Edinburgh: Neill and Company; 1883.

27. Taylor JRE. Thermal insulation of the down and feathers of pygoscelid penguin chicks and the unique properties of penguin feathers. Auk. 1986;103:160–168.

28. Sivak JG. The role of a flat cornea in the amphibious behaviour of the blackfoot penguin (*Spheniscus demersus*) Can J Zool. 1976;54:1341–1345.

29. Sivak JG, Millodot M. Optical performance of the penguin eye in air and water. J Comp Physiol. 1977;119:241–247.

30. Bowmaker JK, Martin GR. Visual pigments and oil droplets in the penguin, *Spheniscus humboldti*. J Comp Physiol A. 1985;156:71–77

31. Meister W. Histological structure of the long bones of penguins. Anat Rec. 1962;143:377–387.

32. Raikow RJ, Bicanovsky L, Bledsoe AH. Auk. 1988. Forelimb joint mobility and the evolution of wing-propelled diving in birds; pp. 446–451.

33. Schreiweis DO. A comparative study of the appendicular musculature of penguins (Aves: Sphenisciformes) Smithsonian Contrib Zool. 1982;341:1–46.

34. Frost PGH, Siegfried WR, Greenwood PJ. Arterio-venous heat exchange systems in the Jackass penguin *Spheniscus demersus*. J Zool. 1975;175:231–241.

35. Groscolas R. Metabolic adaptations to fasting in emperor and king penguins. In: Davis LS, Darby JT, editors. Penguin Biology. San Diego: Academic; 1990. pp. 269–296.

36. Cherel Y, Gilles J, Handrich Y, Le Maho Y. Nutrient reserve dynamics and energetics during long-term fasting in the king penguin (*Aptenodytes patagonicus*) J Zool. 1994;234:1–12.

37. Groscolas R, Robin JP. Long-term fasting and re-feeding in penguins. Comp Biochem Physiol A Mol Integr Physiol. 2001;128:645–655.

38. Gauthier-Clerc M, Le Maho Y, Clerquin Y, et al. Seabird reproduction in an unpredictable environment: how King penguins provide their young chicks with food. Mar Ecol Prog Ser. 2002;237:291–300.

39. Thouzeau C, Le Maho Y, Froget G, et al. Spheniscins, avian β-defensins in preserved stomach contents of the king penguin, *Aptenodytes patagonicus*. J Biol Chem. 2003;278:51053–51058.

40. Thomas DB, Fordyce RE. The heterothermic loophole exploited by penguins. Aust J Zool. 2008;55:317–321.

41. Cairns DK. Plumage Colour in Pursuit-Diving Seabirds: Why Do Penguins Wear Tuxedos? Bird Behav. 1986;6(2)58–65.

42. Croxall JP. Energy costs of incubation and moult in petrels and penguins. J Anim Ecol. 1982;177–194.

43. Barbraud C & Weimerskirch H. Emperor penguins and climate change. Nature. 2001; 411(6834):183–186.

44. Forcada J, Trathan PN, Reid K, et al. Contrasting population changes in sympatric penguin species in association with climate warming. Glob Change Biol. 2006; 12(3):411–423.

45. Fretwell PT & Trathan PN. Emperors on thin ice: three years of breeding failure at Halley Bay. Antarct Sci. In Press.

46. Trivelpiece WZ, Hinke JT, Miller AK, et al. Variability in krill biomass links harvesting and climate warming to penguin population changes in Antarctica. P Natl Acad Sci USA. 2011; 108(18):7625–7628.

47. Lynch HJ, Naveen R, Trathan PN, et al. Spatially integrated assessment reveals widespread changes in penguin populations on the Antarctic Peninsula. Ecology. 2012; 93(6):1367–1377.

48. Mattern T, Meyer S, Ellenberg U, et al. Quantifying climate change impacts emphasises the importance of managing regional threats in the endangered Yellow-eyed penguin. PeerJ. 2017; 5:e3272.

49. Heerah K, Dias MP, Delord K, et al. Important areas and conservation sites for a community of globally threatened marine predators of the Southern Indian Ocean. Biol Conserv. 2019; 234(1):192–201.

50. Li C, Zhang Y, Li J, et al. Two Antarctic penguin genomes reveal insights into their evolutionary history and molecular changes related to the Antarctic environment. GigaScience. 2014; 3(1):27.

51. Trucchi E, Gratton P, Whittington JD, et al. King penguin demography since the last glaciation inferred from genome-wide data. P Roy Soc B-Biol Sci. 2016; 281(1787):20140528.

52. Cristofari R, Bertorelle G, Ancel A, et al.. Full circumpolar migration ensures evolutionary utility in the Emperor penguin. Nat Commun. 2016; 7:pp.11842.

53. Cristofari R, Liu X, Bonadonna F, et al. Climate-driven range shifts of the king penguin in a fragmented ecosystem. Nat Clim Change. 2018; 8(3):pp.245.

54. Le Bohec C, Durant JM, Gauthier-Clerc M, et al. King penguin population threatened by Southern Ocean warming. P Roy Soc B-Biol Sci. 2008; 105(7):2493–2497.

55. Jenouvrier S, Caswell H, Barbraud C, et al. Demographic models and IPCC climate projections predict the decline of an emperor penguin population. P Natl Acad Sci USA. 2009; 106(6):1844–1847.

56. Jenouvrier S, Holland M, Stroeve J, et al. Projected continent-wide declines of the emperor penguin under climate change. Nat Clim Change. 2014; 4(8):715–718.

57. Boessenkool S, Austin JA, Worthy TH, et al. Relict or colonizer? Extinction and range expansion of penguins in southern New Zealand. P Roy Soc B-Biol Sci. 2008; 276(1658):815–821.

58. Clucas GV, Dunn MJ, Dyke G, et al. A reversal of fortunes: climate change ‘winners’ and ‘losers’ in Antarctic Peninsula penguins. Sci Rep-UK. 2014; 4:pp.5024.

59. Younger JL, Clucas GV, Kooyman G, et al. Too much of a good thing; sea ice extent may have forced emperor penguins into refugia during the last glacial maximum. Glob Change Biol. 2015; 21(6):2215–2226.

60. Subramanian S, Beans-Picón G, Swaminathan SK, et al. Evidence for a recent origin of penguins. Biol Letters. 2013: 9(6): 20130748.

61. Gavryushkina A, Heath TA, Ksepka DT, et al. Bayesian total evidence dating reveals the recent crown radiation of penguins. Syst Biol. 2017; 66(1):57–73.

62. Grosser S, Burridge CP, Peucker AJ, et al. Coalescent Modelling Suggests Recent Secondary-Contact of Cryptic Penguin Species. PLoS One. 2015; 10(12):e0144966.

63. Vianna JA, Noll D, Mura-Jornet I, et al. Comparative genome-wide polymorphic microsatellite markers in Antarctic penguins through next generation sequencing. Genet Mol Biol. 2017; 40(3):676–687.

64. Ramos B, González-Acuña D, Loyola DE, et al. Landscape genomics: natural selection drives the evolution of mitogenome in penguins. BMC Genomics. 2018; 19:53.

65. Clucas GV, Younger JL, Kao D, et al. Dispersal in the sub-Antarctic: king penguins show remarkably little population genetic differentiation across their range. BMC Evol Biol. 2016; 16(1):211.

66. Younger JL, Clucas GV, Kao D, et al. The challenges of detecting subtle population structure and its importance for the conservation of Emperor penguins. Mol Ecol. 2017; 26(15):3883–3897.

67. Clucas GV, Younger JL, Kao D, et al. Comparative population genomics reveals key barriers to dispersal in Southern Ocean penguins. Mol Ecol. 2018; 27(23):4680–4697.

68. Younger J, Emmerson L, Southwell C, et al. Proliferation of East Antarctic Adélie penguins in response to historical deglaciation. BMC Evol Biol. 2015; 15(1):236.

69. Zhao H, Li J, Zhang J. Molecular evidence for the loss of three basic tastes in penguins. Curr Biol. 2015; 25(4):R141-R142.

70. Zhang G, Li C, Li Q, et al. Comparative genomics reveals insights into avian genome evolution and adaptation. Science. 2014; 346(6215):1311–1320.

71. Borges R, Khan I, Johnson WE, et al. Gene loss, adaptive evolution and the co-evolution of plumage coloration genes with opsins in birds. BMC Genomics. 2015; 16:751.

72. Jarvis ED, Mirarab S, Aberer AJ, et al. Whole genome analyses resolve early branches in the tree of life of modern birds. Science. 2014; 346(6215):1320–1331.

73. Grosser S, Scofield RP, Waters JM. Multivariate skeletal analyses support a taxonomic distinction between New Zealand and Australian *Eudyptula* penguins (Sphenisciformes: Spheniscidae). Emu. 2017; 177:176-283

74. Bi K, Linderoth T, Vanderpool D, et al. Unlocking the vault: next- generation museum population genomics. Mol Ecol. 2013; 22(24):6018-6032.

75. Stiller J, Zhang G. Comparative phylogenomics, a stepping stone for bird biodiversity studies. Diversity. 2019; 11(7):115.

76. Huang J, Liang X, Xuan Y, et al. A reference human genome dataset of the BGISEQ-500 sequencer. GigaScience. 2017; 6(5):1-9.

77. Edmunds S. HiSeq 4000 sequencing protocol. 2018; dx.doi.org/10.17504/protocols.io.q58dy9w.

78. Huang J, Liang X, Xuan Y, et al. Erratum to: A reference human genome dataset of the BGISEQ-500 sequencer. GigaScience. 2018;7(12):giy144.

79. Teh BT, Lim K, Yong CH, et al. The draft genome of tropical fruit durian (*Durio zibethinus*). Nat Genet. 2017; 49:1633–1641.

80. Heydari M, Miclotte G, Demeester P, et al. Evaluation of the impact of Illumina error correction tools on de novo genome assembly. BMC Bioinformatics. 2017; 18:374.

81. Luo R, Liu B, Xie Y, et al. SOAPdenovo2: an empirically improved memory-efficient short-read de novo assembler. GigaScience. 2012; 1(1):18.

82. Gnerre S, Maccallum I, Przybylski D, et al. High-quality draft assemblies of mammalian genomes from massively parallel sequence data. Proc Natl Acad Sci USA. 2011; 108(4):1513–8.

83. Weisenfeld NI, Kumar V, Shah P, et al. Direct determination of diploid genome sequences. Genome Res. 2017; 5:757–767.

84. Simão FA, Waterhouse RM, Ioannidis P, et al. BUSCO: assessing genome assembly and annotation completeness with single-copy orthologs. Bioinformatics. 2015; 31(19):3210–2.

85. Smit AFA, Hubley R & Green P. RepeatMasker Open-4.0. 2013–2015.

86. Benson G. Tandem repeats finder: a program to analyze DNA sequences. Nucleic Acids Res. 1999; 27(2):573–580.

87. Smit AFA, Hubley RR, Green PR. Open-1.0. 2008–2015. Institute for Systems Biology, Seattle, WA, USA. 2008.

88. Bao W, Kojima KK, Kohany O. Repbase Update, a database of repetitive elements in eukaryotic genomes. Mobile DNA-UK. 2015; 6(1):11.

89. Altschul SF, Gish W, Miller W, et al. Basic local alignment search tool. J Mol Biol. 1990; 215(3): 403–410.

90. She R, Chu JS, Wang K, et al. GenBlastA: enabling BLAST to identify homologous gene sequences. Genome Res. 2009; 19(1):143–9.

91. Birney E, Clamp M, Durbin R. GeneWise and genomewise. Genome Res. 2004; 14(5):988–995.

92. Edgar RC. MUSCLE: multiple sequence alignment with high accuracy and high throughput. Nucleic Acids Res. 2004; 32(5):1792–7.

93. Wheeler DL, Barrett T, Benson DA, et al. Database resources of the national center for biotechnology information. Nucleic Acids Res. 2006; 14(35,suppl_1):D5-12.

94. Silva GG, Dutilh BE, Matthews TD, et al. Combining de novo and reference-guided assembly with scaffold_builder. Source code for biology and medicine. 2013; 8(1):23.

95. Grabherr MG, Haas BJ, Yassour M, et al. Full-length transcriptome assembly from RNA-Seq data without a reference genome. Nat Biotechnol. 2011; 29(7):644.

96. Boeckmann B, Bairoch A, Apweiler R, et al. The SWISS-PROT protein knowledgebase and its supplement TrEMBL in 2003. Nucleic Acids Res. 2003; 31(1):365-70.

97. Jones P, Binns D, Chang HY, et al. InterProScan 5: genome-scale protein function classification. Bioinformatics. 2014; 30(9):1236-40.

98. Kanehisa M, Sato Y, Furumichi M, et al. New approach for understanding genome variations in KEGG. Nucleic Acids Res. 2018; 47(D1): D590-5.

99. Ashburner M, Ball CA, Blake JA, et al. Gene ontology: tool for the unification of biology. Nat Genet. 2000; 25(1):25.

100. Tipene- Matua B, Henaghan M. Establishing a Māori ethical framework for genetic research with Māori. In: Genes, Society and the Future (ed. Henaghan M). 2007; pp. 1–44. Human Genome Research Project, Dunedin, New Zealand.

101. Wilcox PL, Charity JA, Roberts MR, et al. A values-based process for cross-cultural dialogue between scientists and Māori. J Roy Soc New Zeal. 2008; 38:215–227.

102. Hudson M, Milne M, Reynolds P, et al. Te Ara Tika Guidelines for Māori research ethics: a framework for researchers and ethics committee members. 2010. ISBN: 978-1-877495-03-8.

103. Galla SJ, Buckley TR, Elshire R, et al. Building strong relationships between conservation genetics and primary industry leads to mutually beneficial genomic advances. Mol Ecol. 2016; 25(21):5267–81.

104. New Zealand Biodiversity Action Plan 2016 – 2020. Department of Conservation. 2016: ISBN: 978-0-478-15095-7.

105. Ngāi Tahu Taonga Animal Species. Department of Conservation. 2006: RS0082. <https://www.doc.govt.nz/globalassets/documents/about-doc/concessions-and-permits/conservation-revealed/ngai-tahu-taonga-animals-lowres.pdf>

106. Wong PB, Wiley EO, Johnson WE, et al. Tissue sampling methods and standards for vertebrate genomics. GigaScience. 2012; 1(1): 8.

107. Department of Conservation. <https://www.doc.govt.nz/get-involved/apply-for-permits/iwi-consultation/> Accessed 27 July 2019.

108. Greig E. The Māori right to development and new forms of property. Ph.D. Thesis. The University of Otago. 2010.

109. National Human Genome Institute. Reaffirmation and Extension of NHGRI Rapid Data Release Policies: Large-scale Sequencing and Other Community Resource Projects. <https://www.genome.gov/10506537/reaffirmation-and-extension-of-nhgri-rapid-data-release-policies> Accessed 27 July 2019.

110. Toronto International Data Release Workshop Authors. Prepublication data sharing. Nature. 2009; 461:168–170.

111. Lindblad-Toh K, Garber M, Zuk O, et al. A high-resolution map of human evolutionary constraint using 29 mammals. Nature. 2011; 478(7370):476–82.

112. i5K Consortium. The i5K Initiative: advancing arthropod genomics for knowledge, human health, agriculture, and the environment. J Hered. 2013; 104(5):500–600.

113. Koepfli KP, Paten B, Genome 10K Community of Scientists, et al. The Genome 10K Project: a way forward. Annu Rev Anim Biosci. 2015; 3(1):57–111.

114. Wang Y, Zhang C, Wang N, et al. Genetic basis of ruminant headgear and rapid antler regeneration. Science. 2019; 364(6446):eaav6335.

Figures

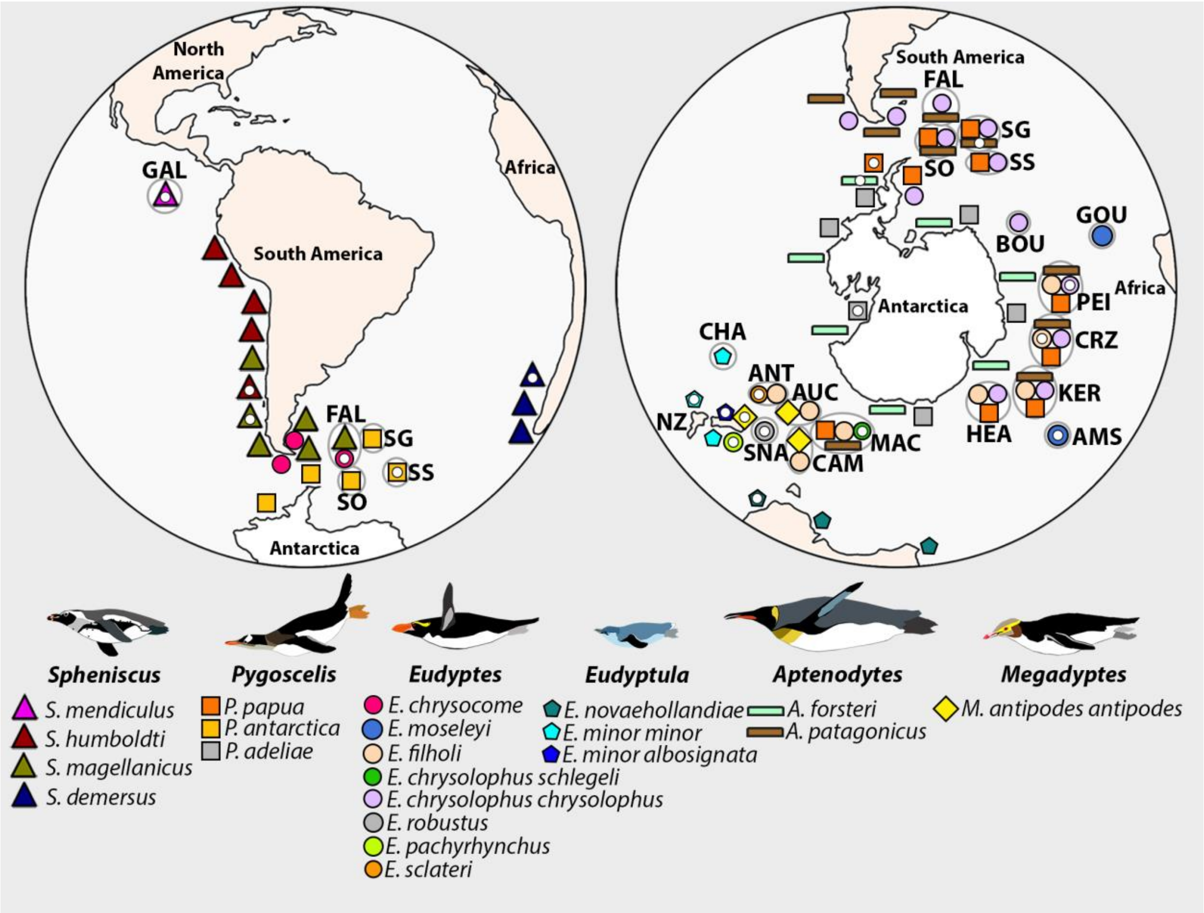

Figure 1. Locations of breeding colonies of penguins and sampling sites for the final genomes, adapted from (1). Sampling locations are shown with a small white ellipse. Note that the sampling location of *Spheniscus humboldti* is unclear, as this individual was bred in the Copenhagen zoo, with ancestors imported from Peru and Chile in 1972. GAL is Galapagos Islands; FAL is Falkland Islands/Malvinas; SG is South Georgia; SO is South Orkney Islands; SS is South Sandwich Islands; BOU is Bouvet; GOU is Gough Island; PEI is Prince Edward/Marion Island; CRZ is Crozet; KER is Kerguelen; HEA is Heard Island; AMS is Amsterdam Island; MAC is Macquarie Island; CAM is Campbell Island; AUC is Auckland Islands; ANT is Antipodes Islands; SNA is The Snares; NZ is New Zealand and CHA is Chatham Islands.

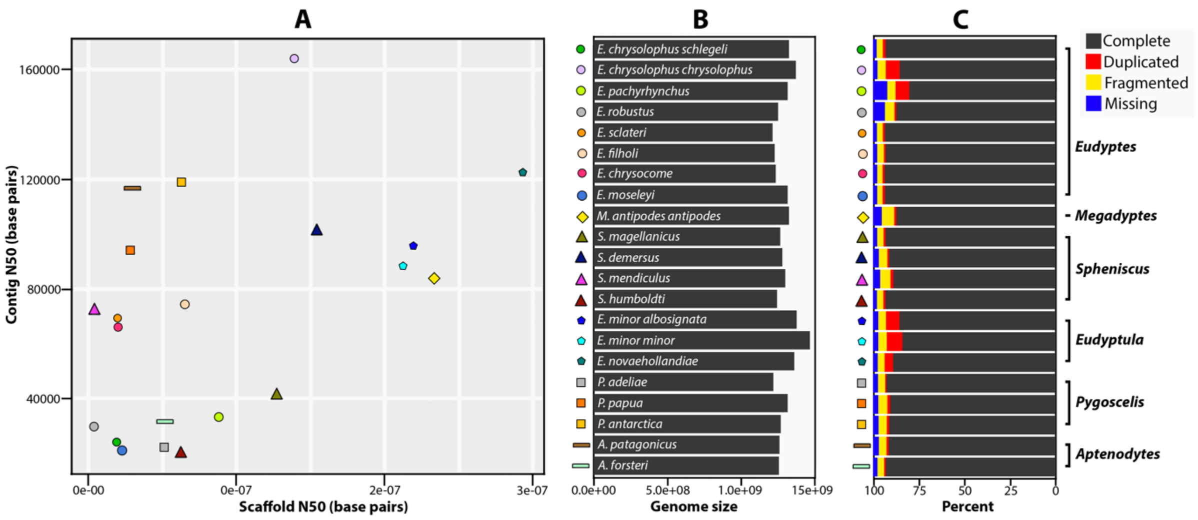

Figure 2. Genome assembly statistics of all penguin species; A) is a dot plot of the quality of each index showing Contig N50 (maximum is *Eudyptes chrysolophus chrysolophus* with 163,848 base pairs, the minimum is *Spheniscus humboldti* with 19,849 base pairs) and Scaffold N50 (the maximum is *Eudyptula novaehollandiae* with 292,802,09 base pairs, the minimum is *Eudyptes robustus* with 363,310 base pairs); B) shows the genome size for each penguin species (the maximum is *Eudyptula minor* with 14,666,868,31 base pairs, the minimum is *Eudyptes sclateri* with 12,117,378,99 base pairs); and C) shows the BUSCO assessments of all penguin genomes, showing the percentage of complete, duplicated, fragmented or missing data. The symbols for each penguin species correspond to the symbols used in Figure 1.

Tables

Table 1. Sample collection information for the 21 penguin genomes (including two obtained in (2).

Latin Name	Common Name	Sample Type	Sampling Location	Sample Label	Date Extracted
Eudyptes chrysolophus schlegeli	Royal	Wild	Green Gorge, Macquarie Island	4458	Oct-17
Eudyptes chrysolophus chrysolophus	Macaroni	Wild	Marion Island, Prince Edward Islands	MP PEI 1	Oct-17
Eudyptes pachyrhynchus	Fiordland-crested	Wild	Harrison Cove, Milford Sound, New Zealand South Island	MS 9	May-17
Eudyptes robustus	Snares-crested	Dunedin Wildlife Hospital	The Snares, New Zealand sub-Antarctic	68M 28/09/13	Sep-18
Eudyptes sclateri	Erect-crested	Wild	Antipodes Island, New Zealand sub-Antarctic	Ant 5	Sep-18
Eudyptes filholi	Eastern rockhopper	Wild	Crozet Island	GS 12	May-16
Eudyptes chrysocome	Southern rockhopper	Wild	Falkland Islands/Malvinas	RH 110-1	May-16
Eudyptes moseleyi	Northern rockhopper	Wild	Amsterdam Island	NRP 118-1	May-16
Megadyptes antipodes antipodes	Yellow-eyed	Wild	Otago Peninsula, New Zealand South Island	OT 2 9/2/18	Aug-18
Spheniscus magellanicus	Magellanic	Wild	Chiloe Island, Chile	AH 6	May-16
Spheniscus demersus	Jackass	Wild	Luderitz, Namibia	AP 173	Jul-18
Spheniscus mendiculus	Galápagos	Wild	Galápagos Islands	GAPE 212	Oct-17
Spheniscus humboldti	Humboldt	Copenhagen Zoo	Peru and Chile lineage	Z-67-15	Oct-16

Eudyptula minor albosignata	White-flippered	Christchurch Antarctic Centre	Banks Peninsula, Canterbury, New Zealand South Island	Fred	Jul-18
Eudyptula minor minor	Little blue	National Aquarium of New Zealand	New Zealand North Island	Gonzo	Aug-18
Eudyptula novaehollandiae	Fairy	Wild	Phillip Island, Victoria, Australia	10/9/18-1	Oct-18
Pygoscelis adeliae	Adélie	Wild	Inexpressible Island, Antarctica	From Li *et al.*, (2014)	From (2)
Pygoscelis papua	Gentoo	Wild	West Antarctic Peninsula, Antarctica	Gentoo penguin DNA -4	Jan-18
Pygoscelis antarctica	Chinstrap	Wild	Thule Island, South Sandwich Islands	CP TH 060	Nov-17
Aptenodytes patagonicus	King	Wild	Fortuna Bay, South Georgia	KP FORT 001	Nov-17
Aptenodytes forsteri	Emperor	Wild	Emperor Island, Antarctica	From Li *et al.*, (2014)	From (2)

Table 2. Details of the sequencing platform used and the data statistics for 21 penguin genomes. HiSeq X ten was used for sequencing small insert size libraries; HiSeq 4000 was used for sequencing mate-pair libraries.

Species	Sequencing Platform	Raw Data (Gbp)	Clean Data (Gbp)
Eudyptes schlegeli	BGIseq500	402.6	296.6
Eudyptes chrysolophus chrysolophus	BGIseq500	145.9	126.9
Eudyptes pachyrhynchus	HiSeq X ten and HiSeq 4000	146.4	104.7
Eudyptes robustus	HiSeq X ten and HiSeq 4000	171.2	107.6
Eudyptes sclateri	HiSeq X ten and HiSeq 4000	156.2	103.2
Eudyptes filholi	HiSeq X ten and HiSeq 4000	195	146.8
Eudyptes chrysocome	HiSeq X ten and HiSeq 4000	195.1	111.6
Eudyptes moseleyi	HiSeq X ten and HiSeq 4000	173.6	133.1
Megadyptes antipodes antipodes	BGIseq500	111.9	104.1
Spheniscus magellanicus	HiSeq X ten and HiSeq 4000	212.6	150.7
Spheniscus demersus	BGIseq500	141.1	131.3
Spheniscus mendiculus	BGIseq500	112.2	104.4
Spheniscus humboldti	HiSeq X ten and HiSeq 4000	208.8	137.2
Eudyptula minor albosignata	BGIseq500	132.5	124.8
Eudyptula minor minor	BGIseq500	121.4	112.7
Eudyptula novaehollandiae	BGIseq500	180.4	168.5
Pygoscelis papua	BGIseq500	134.5	124
Pygoscelis antarctica	BGIseq500	154.5	139.7
Aptenodytes patagonicus	BGIseq500	147.6	134

Table 3. Repeat annotation results for 21 penguins and five outgroups.

Species	DNA		LINE		SINE		LTR		Other		Unknown		TRF		Total	
	Length (bp)	% in genome	Length (bp)	% in genome	Length (bp)	% in genome	Length (bp)	% in genome	Length (bp)	% in genome	Length (bp)	% in genome	Length (bp)	% in genome	Length (bp)	% in genome
Eudyptes chrysolophus schlegeli	10,967,993	0.837	56,600,258	4.32	1,886,042	0.144	23,772,820	1.81	1,709	0.00013	7,181,843	0.548	27,041,073	2.06	122,778,314	9.37
Eudyptes chrysolophus chrysolophus	9,840,577	0.719	81,007,897	5.92	2,325,630	0.17	42,950,488	3.14	2,109	0.000154	6,349,669	0.464	7,624,752	0.557	147,221,283	10.8
Eudyptes pachyrhynchus	9,700,549	0.74	57,537,411	4.39	1,761,671	0.134	26,951,871	2.06	7,163	0.000546	8,778,995	0.67	15,315,109	1.17	115,154,499	8.78
Eudyptes robustus	10,035,161	0.804	54,876,908	4.4	1,694,896	0.136	21,900,240	1.75	1,197	0.000096	6,793,784	0.544	13,082,350	1.05	105,161,038	8.42
Eudyptes sclateri	9,603,106	0.793	57,388,336	4.74	1,648,534	0.136	22,555,283	1.86	2,155	0.000178	5,455,896	0.45	7,045,858	0.581	101,615,942	8.39
Eudyptes filholi	9,447,824	0.772	58,471,185	4.78	1,894,915	0.155	23,146,953	1.89	2,662	0.000217	8,146,713	0.666	7,812,634	0.638	104,766,914	8.56
Eudyptes chrysocome	9,067,962	0.737	58,040,264	4.71	1,608,644	0.131	22,515,809	1.83	2,095	0.00017	7,321,722	0.595	7,332,611	0.596	103,276,447	8.39
Eudyptes moseleyi	9,367,954	0.717	58,805,425	4.5	1,990,469	0.152	23,593,767	1.81	2,664	0.000204	9,786,633	0.749	45,959,293	3.52	141,103,330	10.8
Megadyptes antipodes antipodes	9,608,349	0.729	78,978,618	5.99	1,728,524	0.131	46,464,418	3.53	1,059	0.00008	8,168,785	0.62	7,802,048	0.592	148,977,693	11.3
Spheniscus magellanicus	10,393,349	0.823	65,351,067	5.18	1,812,355	0.144	26,759,543	2.12	1,546	0.000122	9,851,237	0.78	10,398,934	0.824	118,099,179	9.35
Spheniscus demersus	9,811,467	0.767	72,969,293	5.71	1,610,171	0.126	34,709,683	2.72	1,509	0.000118	20,385,557	1.59	6,712,698	0.525	130,219,709	10.2
Spheniscus mendiculus	10,792,037	0.83	80,340,773	6.18	1,694,428	0.13	43,906,026	3.38	2,265	0.000174	13,023,335	1	7,421,979	0.571	147,721,431	11.4
Spheniscus humboldti	9,850,523	0.792	63,427,971	5.1	2,095,439	0.169	26,032,187	2.09	2,610	0.00021	7,051,364	0.567	10,846,563	0.872	115,794,679	9.31
Eudyptula minor albosignata	10,287,254	0.749	86,732,446	6.31	2,230,442	0.162	49,548,759	3.61	2,285	0.000166	10,370,641	0.755	8,661,285	0.63	160,541,239	11.7
Eudyptula minor minor	10,691,141	0.729	95,293,482	6.5	1,790,448	0.122	62,515,534	4.26	2,245	0.000153	8,460,299	0.577	9,083,782	0.619	183,740,284	12.5
Eudyptula novaehollandiae	10,542,998	0.777	87,757,466	6.46	1,654,900	0.122	53,144,657	3.92	1,522	0.000112	12,914,720	0.951	8,531,830	0.629	164,989,801	12.2

Pygoscelis adeliae	8,905,965	0.732	52,089,816	4.28	1,643,684	0.135	17,580,686	1.45	1,685	0.000139	6,938,950	0.57	8,565,483	0.704	93,839,128	7.71
Pygoscelis papua	10,878,036	0.831	79,578,503	6.08	1,683,574	0.129	47,004,788	3.59	2,163	0.000165	8,393,877	0.641	7,857,958	0.6	151,240,877	11.6
Pygoscelis antarctica	10,021,109	0.792	75,467,782	5.96	1,660,023	0.131	36,515,988	2.89	1,645	0.00013	5,649,521	0.446	6,850,733	0.541	133,620,728	10.6
Aptenodytes patagonicus	9,883,830	0.786	72,143,844	5.74	1,669,248	0.133	33,210,718	2.64	2,273	0.000181	5,987,857	0.476	6,868,165	0.547	126,913,554	10.1
Aptenodytes forsteri	9,648,988	0.769	47,421,228	3.78	1,755,252	0.14	14,998,979	1.2	1,055	0.000084	5,984,114	0.477	28,075,518	2.24	103,411,467	8.24
Hydrobates tethys	10,174,835	0.851	43,642,750	3.65	1,593,248	0.133	13,363,132	1.12	1,780	0.000149	6,044,078	0.505	10,375,034	0.868	82,871,365	6.93
Oceanites oceanicus	8,172,757	0.694	53,982,174	4.58	1,518,213	0.129	19,561,601	1.66	2,202	0.000187	6,101,243	0.518	10,501,141	0.891	97,111,623	8.24
Thalassarche chlororhynchos	10,390,449	0.929	41,856,139	3.74	1,766,094	0.158	14,374,696	1.29	2,035	0.000182	5,822,959	0.521	6,943,803	0.621	79,491,403	7.11
Taeniopygia guttata	5,985,051	0.486	51,144,902	4.15	883,324	0.0717	50,817,604	4.12	4,713	0.000383	13,099,829	1.06	25,800,776	2.09	137,289,217	11.1
Gallus gallus	13,929,789	1.33	78,779,279	7.52	571,067	0.0545	21,043,114	2.01	1,638	0.000156	20,514,532	1.96	10,603,861	1.01	129,394,288	12.4

Table 4. Protein coding gene statistics of all 21 penguin genomes and five outgroups.

Species	Number of protein coding genes	Mean gene length (bp)	Mean coding sequence length (bp)	Mean exons per gene	Mean exon length (bp)	Mean intron length (bp)
Eudyptes chrysolophus schlegeli	17,191	18,860	1,351	7.9	171	2,540
Eudyptes chrysolophus chrysolophus	16,311	20,248	1,392	8.2	170	2,623
Eudyptes pachyrhynchus	19,170	17,394	1,306	7.4	178	2,535
Eudyptes robustus	17,126	16,254	1,295	7.4	174	2,329
Eudyptes sclateri	15,786	19,627	1,402	8.2	171	2,527
Eudyptes filholi	15,963	19,959	1,407	8.2	171	2,562
Eudyptes chrysocome	16,280	19,436	1,382	8.1	171	2,555
Eudyptes moseleyi	16,812	19,767	1,370	8	171	2,621
Spheniscus magellanicus	16,795	19,311	1,381	8.1	171	2,535
Megadyptes antipodes antipodes	16,563	18,509	1,334	7.8	171	2,533
Spheniscus demersus	16,134	19,029	1,344	7.8	171	2,584
Spheniscus mendiculus	16,390	17,097	1,311	7.6	172	2,382
Spheniscus humboldti	16,587	19,642	1,387	8.1	170	2,558
Eudyptula minor albosignata	17,424	18,837	1,338	7.8	172	2,574
Eudyptula minor minor	17,802	19,078	1,349	7.8	172	2,598
Eudyptula novaehollandiae	17,188	19,271	1,355	7.9	172	2,609
Pygoscelis adeliae	14,463	20,595	1,385	8.3	168	2,648
Pygoscelis papua	16,698	18,276	1,333	7.8	172	2,503
Pygoscelis antarctica	15,488	19,520	1,381	8.1	171	2,558
Aptenodytes patagonicus	15,195	19,596	1,384	8.1	170	2,552
Aptenodytes forsteri	15,593	19,844	1,381	8.1	170	2,584
Hydrobates tethys	15,915	17,898	1,344	8.1	165	2,323
Oceanites oceanicus	16,055	17,936	1,356	8	170	2,377
Thalassarche chlororhynchos	13,347	10,029	1,110	6.4	175	1,667
Taeniopygia guttata	19,174	14,787	1,196	7.2	167	2,198
Gallus gallus	17,883	16,965	1,414	8.3	171	2,135

Table 5. Function annotation results for protein coding genes for 21 penguins and five outgroups.

Species	Swissprot		KEGG		Interpro		Overall	
	Number	Percent	Number	Percent	Number	Percent	Number	Percent
Eudyptes chrysolophus schlegeli	16,739	97.37	15,347	89.27	16,916	98.40	17,064	99.26
Eudyptes chrysolophus chrysolophus	15,863	97.25	14,646	89.79	16,051	98.41	16,191	99.26
Eudyptes pachyrhynchus	18,680	97.44	17,250	89.98	18,873	98.45	19,028	99.26
Eudyptes robustus	16,580	96.81	15,500	90.51	16,816	98.19	16,988	99.19
Eudyptes sclateri	15,383	97.45	14,172	89.78	15,540	98.44	15,664	99.23
Eudyptes filholi	15,555	97.44	14,362	89.97	15,696	98.33	15,840	99.23
Eudyptes chrysocome	15,692	96.39	14,732	90.49	15,977	98.14	16,148	99.19
Eudyptes moseleyi	16,377	97.41	15,153	90.13	16,540	98.38	16,688	99.26
Megadyptes antipodes antipodes	15,755	95.12	14,993	90.52	16,264	98.19	16,445	99.29
Spheniscus magellanicus	16,371	97.48	15,136	90.12	16,532	98.43	16,670	99.26
Spheniscus demersus	15,388	95.38	14,579	90.36	15,839	98.17	16,001	99.18
Spheniscus mendiculus	15,714	95.88	14,801	90.31	16,090	98.17	16,254	99.17
Spheniscus humboldti	16,172	97.50	14,954	90.15	16,319	98.38	16,460	99.23
Eudyptula minor albosignata	16,615	95.36	15,778	90.55	17,098	98.13	17,297	99.27
Eudyptula minor minor	16,994	95.46	16,073	90.29	17,476	98.17	17,663	99.22
Eudyptula novaehollandiae	16,423	95.55	15,561	90.53	16,892	98.28	17,060	99.26
Pygoscelis adeliae	13,964	96.55	13,054	90.26	14,220	98.32	14,348	99.20
Pygoscelis papua	15,931	95.41	15,097	90.41	16,378	98.08	16,553	99.13
Pygoscelis antarctica	15,050	97.17	13,853	89.44	15,224	98.30	15,360	99.17
Aptenodytes patagonicus	14,808	97.45	13,493	88.80	14,954	98.41	15,063	99.13
Aptenodytes forsteri	15,053	96.54	14,112	90.50	15,308	98.17	15,478	99.26
Hydrobates tethys	15,493	97.35	14,273	89.68	15,628	98.20	15,775	99.12
Oceanites oceanicus	15,622	97.30	14,412	89.77	15,775	98.26	15,919	99.15
Thalassarche chlororhynchos	12,958	97.09	11,881	89.02	13,072	97.94	13,219	99.04
Taeniopygia guttata	18,367	95.79	17,115	89.26	18,537	96.68	18,918	98.66
Gallus gallus	16,760	93.72	15,585	87.15	17,079	95.50	17,263	96.53

Supplementary Tables

Supplementary Table 1. Sampling and permitting details of all penguin samples tested.

Latin Name	Sample Label	Sampling Location	Additional Samples Tested	Additional Sampling Locations	Animal Ethics	Permit Details	New Zealand consultation
Eudyptes chrysolophus schlegeli	ROPE 4458 (Gary Miller)	Green Gorge, Macquarie Island	ROPE 4449 (Gary Miller), ROPE 4461 (Gary Miller)	Green Gorge, Macquarie Island	Minister for Primary Industries and Water, Tasmania, Licence to Conduct Research 58932 (Gary Miller)	Department of Primary Industries, Water and Environment permit to take wildlife FA 06378 (Gary Miller)	NA
Eudyptes chrysolophus chrysolophus	MP PEI 1 (Peter Ryan)	Marion Island, Prince Edward Islands	MP PEI 2 (Peter Ryan), MP PEI 3 (Peter Ryan), MP PEI 4 (Peter Ryan), MP PEI 5 (Peter Ryan), MP PEI 6 (Peter Ryan), MP PEI 7 (Peter Ryan)	Marion Island, Prince Edward Islands	UCT Science Faculty Animal Ethics Committee permit 2013/V5/PR (Peter Ryan)	Samples collected with permission from the South African Department of Environment Affairs (Peter Ryan)	NA
Eudyptes pachyrhynchus	MS 9 (Thomas Mattern/Theresa Cole)	Harrison Cove, Milford Sound, New Zealand South Island	MS 8 (Thomas Mattern/Theresa Cole), WH 01 2016 (Ursula Ellenberg/David Houston)	Harrison Cove, Milford Sound, New Zealand South Island; Codfish Island, Foveaux Strait, New Zealand	University of Otago Animal Ethics Committee 61/2016 (Jonathan Waters/Theresa Cole); Animal Ethics number AUP#38/14 (Thomas Mattern)	50436-FAU (Theresa Cole), 54288-DOA (Theresa Cole), OT-25557-DOA (Bruce Robertson), RES-38882 (Thomas Mattern)	University of Otago Ngāi Tahu Research Consultation Committee (Jonathan Waters/Theresa Cole); Department of Conservation Māori Consultation (Theresa Cole); Department of Conservation Māori Consultation (Thomas Mattern)
Eudyptes robustus	68M 28/09/13 (David Thompson)	The Snares	38F 27/09/13 (David Thompson), 48 gvn 28/09/13 (David Thompson)	The Snares	University of Otago Animal Ethics Committee 61/2016 (Jonathan Waters/Theresa Cole); NIWA Animal Ethics Committee	62361-FAU (Lisa Argilla), 50436-FAU (Theresa Cole), 50437-DOA (Theresa Cole), 35682-FAU (David Thompson), OT-25557-DOA (Bruce Robertson)	University of Otago Ngāi Tahu Research Consultation Committee (Jonathan Waters/Theresa Cole); Department of Conservation Māori Consultation (Theresa Cole); Department of

					Approvals (David Thompson)		Conservation Māori Consultation (David Thompson)
Eudyptes sclateri	Ant 5 (Chris Rickard/Sarah Fraser)	Antipodes Island, New Zealand sub-Antarctic	Ant 48 (Chris Rickard/Sarah Fraser), Ant 11 (Chris Rickard/Sarah Fraser), 180236 Purakaunui Bay 11/3/18 (Lisa Argilla)	Antipodes Island, New Zealand sub-Antarctic; Purakaunui Bay, New Zealand South Island	National Institute of Water and Atmospheric Research ethics approval (David Thompson); University of Otago Animal Ethics Committee 61/2016 (Jonathan Waters/Theresa Cole)	SO-28366-FAU (Kath Walker/Graeme Elliott/Chris Rickard/Sarah Fraser/David Thompson); 62361-FAU (Lisa Argilla), 50436-FAU (Theresa Cole), 50464-DOA (Theresa Cole), OT-25557-DOA (Bruce Robertson)	University of Otago Ngāi Tahu Research Consultation Committee (Jonathan Waters/Theresa Cole); Department of Conservation Māori Consultation (Theresa Cole)
Eudyptes filholi	GS 12 (Yves Cherel)	Possession Island, Crozet Islands	NA	NA	Fieldwork was approved by the Ethic Committee of the French Polar Institute (IPEV) (Yves Cherel)	The permit (no number) was delivered to the IPEV program no. 109 (H. Weimerskirch)	NA
Eudyptes chrysocome	RH 110 (Dee Boersma)	Falkland Islands/Malvinas	NA	NA	IACUC eGC1 # A145289 (Dee Boersma)	MBTA Permit #: MB098566 (Robert Faucett); Sample collected with permission from the Falkland Islands Government (Amy van Buren/Dee Boersma) and the Burke Museum at the University of Washington (Amy van Buren/Dee Boersma)	NA
Eudyptes moseleyi	NRP 118 (Yves Cherel)	Amsterdam Island	NRP 115 (Yves Cherel)	Amsterdam Island	Fieldwork was approved by the Ethic Committee of the French Polar Institute (IPEV) (Yves Cherel)	The permit (no number) was delivered to the IPEV program no. 109 (H. Weimerskirch)	NA
Megadyptes antipodes antipodes	OT 2 9/2/18 (Melanie Young)	Otago Peninsula, New Zealand South Island	OT 3 9/2/18 (Melanie Young), OT 4 9/2/18 (Melanie Young), OT 5 9/2/18 (Melanie Young), OT 6 9/2/18 (Melanie Young)	Otago Peninsula, New Zealand South Island	University of Otago Animal Ethics Committee 48/2016 (Bruce Robertson/Melanie Young)	50925-FAU (Melanie Young)	Department of Conservation Māori Consultation (Theresa Cole)

Spheniscus magellanicus	AH 6 (Juan Bouzat	Chiloé Island, Chile	PA1 (Juan Bouzat), PA2 (Juan Bouzat), PA3 (Juan Bouzat), PA4 (Juan Bouzat), AH3 (Juan Bouzat), AH4 (Juan Bouzat), AH5 (Juan Bouzat), S1 (Juan Bouzat), S2 (Juan Bouzat), S3 (Juan Bouzat), S4 (Juan Bouzat), E1 (Dee Boersma/Juan Bouzat), E2 (Dee Boersma/Juan Bouzat), E3 (Dee Boersma/Juan Bouzat), E4 Dee Boersma/Juan Bouzat) JAC29 (Dee Boersma/Juan Bouzat), JAC34 (Dee Boersma/Juan Bouzat), JAC74 (Dee Boersma/Juan Bouzat), JAC76 (Dee Boersma/Juan Bouzat), H1 (Dee Boersma/Juan Bouzat), H2 (Dee Boersma/Juan Bouzat), H3 (Dee Boersma/Juan Bouzat), H20 (Dee Boersma/Juan Bouzat), M1 (Dee Boersma/Juan Bouzat), M2 (Dee Boersma/Juan Bouzat), M3 (Dee Boersma/Juan Bouzat), M4 (Dee Boersma/Juan Bouzat)	Atlantic and Pacific, various locations	Division of Fisheries of the National Government of Chile and the Forestry National Division (CONAF) Permit # 3523 (Juan Bouzat) University of Washington IACUC (Dee Boersma)	Division of Fauna and Flora of the Government of Argentina Permit # 18/02FyFS; USDA-APHIS Importation Permit # 42579 (Robert Faucett/Dee Boersma; Burke Museum at the University of Washington)	NA
Spheniscus demersus	AP 173 (Kim Labuschagne)	Luderitz, Namibia	NUP 1 (Lisa Nupen), NUP 39 (Lisa Nupen), NUP 77 (Lisa Nupen), AP 127 (Kim Labuschagne)	St Croix Island, Eastern Cape Coast, South Africa; Dassen Island, South Western coast South Africa; Mercury Island, Namibia; Luderitz, Namibia	University of Cape Town Science Faculty Animal Ethics clearance number 2009/V21/LN (Lisa Nupen); SANCCOB (Kim Labuschagne)	the South African Department of Environmental Affairs permit number RES2010/66 (Lisa Nupen); CapeNature collection permit number AAA-004-00520-0035 (Lisa Nupen); South African National Parks approval (Lisa Nupen); the Namibian Ministry of Environment, Natural Resources and Transport: Wildlife Enforcement and Permits Division, Wildlife Trade and Conservation Section (Lisa Nupen); CITES permit 204790 (Lisa Nupen); NZG/RES/P17/42; the South African Department of	NA

						Environmental Affairs Standing permit number S07901 (Kim Labuschagne), National Zoological Garden, South African National Biodiversity Institute NZG/RES/P17/42 (Kim Labuschagne) CITES Permit/Certificate No. 207882 (Kim Labuschagne)	
Spheniscus mendiculus	GAPE 212 (Patricia Parker)	Galápagos Islands	GAPE PR 21 (Patricia Parker), GAPE 458 (Patricia Parker), GAPE PR 2039 (Patricia Parker)	Galápagos Islands	University of Missouri – St. Louis IACUC Permit (Patricia Parker)	United States Veterinary Permit for Importation and Transport of Controlled Materials and Organisms and Vectors Permit Number: 47418 (Patricia Parker); U.S. Fish and Wildlife Service Declaration for Importation or Exportation of fish or wildlife O.M.B. No. 1018-0012 (Patricia Parker); Department of the Interior U.S. Fish and Wildlife Service, Federal Fish and Wildlife Permit Number: MA42831A-0 (Jeffrey Brown/Dana Brown/Patricia Parker); Convenio de Cooperacion Interinstitucional Letter (Patricia Parker)	NA
Spheniscus humboldti	Z-67-15 (Mads Bertelsen)	Peru and Chile lineage	NA	NA	Z-67-15 2016-10, Collection approval from Copenhagen Zoo internal scientific committee (IACUC) (Mads Bertelsen)	Z-67-15 2016-10, Collection approval from Copenhagen Zoo internal scientific committee (IACUC) (Mads Bertelsen)	NA
Eudyptula minor albosignata	Fred (Pauline Howard)	Banks Peninsula, Canterbury, New Zealand South Island	Dane (Pauline Howard), BP 06 (Stefanie Grosser), BP 17 (Stefanie Grosser)	Christchurch Antarctic Centre, originally Banks Peninsula, New Zealand South Island; Otanerito Bay,	University of Otago Animal Ethics Committee 59/2010 (Yolanda van Heezik/Scott Flemming)	50436-FAU (Theresa Cole), 54288-DOA (Theresa Cole), 39997-FAU (Thomas Stracke/Kristina Schutt), CA-32742-CAP (International Antarctic Centre Ltd), CA-28817-FAU (Scott Flemming)	University of Otago Ngāi Tahu Research Consultation Committee (Yolanda van Heezik/Scott Flemming); Department of Conservation Māori

				Banks Peninsula, New Zealand South Island			Consultation (Theresa Cole)
Eudyptula minor minor	Gonzo (Helen Taylor)	New Zealand North Island	Bop 48 (Stefanie Grosser), LBP Waitarere 13/4/18 (Emily Kay), Peppa (Helen Taylor), Lulle (Helen Taylor), Dora F (Helen Taylor), Elmo F (Helen Taylor), Draco (Helen Taylor)	Bay of Plenty, New Zealand North Island; Waitarere, New Zealand North Island; National Aquarium of New Zealand, originally New Zealand North Island	Animal Ethics as referred to in (3)	OT-34124-DOA (Stefanie Grosser), OT-25557-DOA (Bruce Robertson), 36555-FAU (Wildbase Hospital); 50436-FAU (Theresa Cole), 54288-DOA (Theresa Cole), 40460-CAP (National Aquarium of New Zealand)	Department of Conservation Māori Consultation (Theresa Cole)
Eudyptula novaehollandiae	10/9/18-1 (Peter Dann)	Phillip Island, Victoria, Australia	Oa 201 (Stefanie Grosser), Oa 93 (Stefanie Grosser), 10/9/18-2 (Peter Dann)	Otago, New Zealand South Island; Phillip Island, Victoria, Australia	Animal Ethics as referred to in (3); Phillip Island Nature Park Animal Ethics Committee 3.2018	OT 34124-DOA (Stefanie Grosser), OT-25557-DOA (Bruce Robertson), 50436-FAU (Theresa Cole), 54288-DOA (Theresa Cole), DELWP 10008780 (Peter Dann)	Department of Conservation Māori Consultation (Theresa Cole)
Pygoscelis papua	Gentoo penguin DNA -4	West Antarctic Peninsula, Antarctica	GP MOOT 011 (Tom Hart), GP OH 101 (Tom Hart), GP 006 (Tom Hart)	West Antarctic Peninsula, Antarctica	Oxford University Animal Ethics committee (Tom Hart)	FCO 34/2016 (Tom Hart)	NA
Pygoscelis antarctica	CP TH 060 (Tom Hart)	Thule Island, South Sandwich Islands	CP TH 058 (Tom Hart)	Thule Island, South Sandwich Islands	ZSL animal ethics committee (Tom Hart), Oxford Animal Ethics Committee (Tom Hart)	Letter of permit from GSGSSI no number (Tom Hart); David Lambert Permit	NA
Aptenodytes patagonicus	KP FORT 001 (Tom Hart)	Fortuna Bay, South Georgia	KP FORT 002 (Tom Hart), KP FORT 003 (Tom Hart), KP FORT 010 (Tom Hart), King penguin DNA (Tom Hart/Steven Fiddaman)	Fortuna Bay, South Georgia	ZSL animal ethics committee (Tom Hart); Oxford Animal Ethics Committee (Tom Hart)	Letter of permit from GSGSSI no number (Tom Hart)	NA

Supplementary Table 2. Assemblers and Kmer sizes used for each penguin. The Kmer sizes were only for species assembled by SOAPdenovo2. Kmer sizes of *Pygoscelis adeliae* and *Aptenodytes forsteri* were from (2).

Species	Assembler	Kmer size (bp)
Eudyptes chrysolophus schlegeli	SOAPdenovo2-2.04	25
Eudyptes chrysolophus chrysolophus	Supernova-2.0.0	NA
Eudyptes pachyrhynchus	SOAPdenovo2-2.04	35
Eudyptes robustus	SOAPdenovo2-2.04	35
Eudyptes sclateri	Allpaths-lg	NA
Eudyptes filholi	Allpaths-lg	NA
Eudyptes chrysocome	Allpaths-lg	NA
Eudyptes moseleyi	SOAPdenovo2-2.04	35
Megadyptes antipodes antipodes	Supernova-2.0.0	NA
Spheniscus magellanicus	SOAPdenovo2-2.04	25
Spheniscus demersus	Supernova-2.0.0	NA
Spheniscus mendiculus	Supernova-2.0.0	NA
Spheniscus humboldti	SOAPdenovo2-2.04	43
Eudyptula minor albosignata	Supernova-2.0.0	NA
Eudyptula minor minor	Supernova-2.0.0	NA
Eudyptula novaehollandiae	Supernova-2.0.0	NA
Pygoscelis adeliae	SOAPdenovo2	19
Pygoscelis papua	Supernova-2.0.0	NA
Pygoscelis antarctica	Supernova-2.0.0	NA
Aptenodytes patagonicus	Supernova-2.0.0	NA
Aptenodytes forsteri	SOAPdenovo2	19

Supplementary Table 3. Information of 71 avian transcriptomic samples downloaded from NCBI.

Organism Name	Order	Family	Number of Samples	Date Publicly Released	SRP ID	Sequencing Platform
Anas platyrhynchos	Anseriformes	Anatidae	2	4-Feb-13	SRP018391	Illumina HiSeq 2000
Aegypius monachus	Falconiformes	Accipitridae	1	29-Apr-14	SRP041562	Illumina HiSeq 2500
Alectoris rufa	Galliformes	Phasianidae	8	26-Nov-14	SRP050314	Illumina Genome Analyzer IIx, SE
Anas platyrhynchos	Anseriformes	Anatidae	4	27-Mar-13	SRP020086	Illumina HiSeq 2000
Anser cygnoides	Anseriformes	Anatidae	1	15-Jan-14	SRP035437	Illumina HiSeq 2000, SE
Anser sp.(goose)	Anseriformes	Anatidae	1	26-Dec-13	SRP034727	Illumina HiSeq 2000
Apteryx australis mantelli	Apterygiformes	Apterygidae	1	20-Dec-11	SRP003481	454 GS FLX, SE
Carduelis chloris	Passeriformes	Fringillidae	2	1-Apr-14	SRP040761	Illumina HiSeq 2500
Corvus macrorhynchos	Passeriformes	Corvidae	1	29-Oct-13	SRP032433	454 GS FLX Titanium, SE
Coturnix japonica	Galliformes	Phasianidae	1	6-May-14	SRP043003	454 GS FLX, SE
Coturnix japonica	Galliformes	Phasianidae	2	24-Sep-15	SRP066885	Illumina HiSeq 2000
Cyanopica cyana	Passeriformes	Corvidae	1	16-Mar-15	SRP056221	Illumina HiSeq 2000, SE
Dromaius novaehollandiae	Casuariiformes	Dromaiidae	1	18-Mar-13	SRP019802	Illumina HiSeq 2000
Gallinago media	Charadriiformes	Scolopacidae	14	30-Oct-12	SRP016881	454 GS FLX Titanium, SE
Haemorhous mexicanus	Passeriformes	Fringillidae	2	6-Mar-13	SRP018959	Illumina HiSeq 2000
Junco hyemalis	Passeriformes	Passerellidae	2	28-Jul-14	SRP052228	Illumina HiSeq 2000, SE
Junco hyemalis	Passeriformes	Passerellidae	1	13-Apr-12	SRP012466	454 GS FLX Titanium, SE
Lamprotornis superbus	Passeriformes	Sturnidae	2	4-Sep-14	SRP046157	Illumina HiSeq 2000, SE
Meleagris gallopavo	Galliformes	Phasianidae	2	3-Jun-14	SRP042724	Illumina Genome Analyzer IIx, SE
Melospiza melodia	Passeriformes	Passerellidae	2	17-Mar-14	SRP040239	Illumina HiSeq 2000
Numida meleagris	Galliformes	Numididae	3	6-Jun-12	SRP021481	Illumina HiSeq 2000, SE
Paradoxornis webbianus bulomachus (Sinosuthora webbiana)	Passeriformes	Sylviidae	1	1-Sep-11	SRP007844	Illumina HiSeq 2000, SE
Parus major	Passeriformes	Paridae	8	20-Dec-11	SRP004680	454 GS FLX Titanium, SE
Phylloscopus trochilus(acredula)	Passeriformes	Phylloscopidae	2	25-Jul-12	SRP014608	454 GS FLX Titanium, SE
Pseudopodoces humilis	Passeriformes	Paridae	2	1-Mar-13	SRP018927	Illumina HiSeq 2000
Uraeginthus granatina	Passeriformes	Estrildidae	1	22-Aug-13	SRP029159	454 GS FLX, SE
Zonotrichia albicollis	Passeriformes	Emberizidae	2	30-Aug-13	SRP029385	Illumina HiSeq 2000, SE
Zonotrichia leucophrys gambelii	Passeriformes	Emberizidae	1	10-Apr-14	SRP041107	Illumina HiSeq 2500

Supplementary References

1. Cole TL, Ksepka DT, Mitchell KJ, et al. Mitogenomes uncover extinct penguin taxa and reveal island formation as a key driver of speciation. Mol Biol Evol. 2019; 36(4):784–797.

2. Li C, Zhang Y, Li J, et al. Two Antarctic penguin genomes reveal insights into their evolutionary history and molecular changes related to the Antarctic environment. GigaScience. 2014; 3(1):27.

3. Grosser S. Molecular systematics and phylogeography of little penguins. Ph.D. Thesis. The University of Otago. 2015.

Figure 1

Click here to download Figure Figure 1.tif

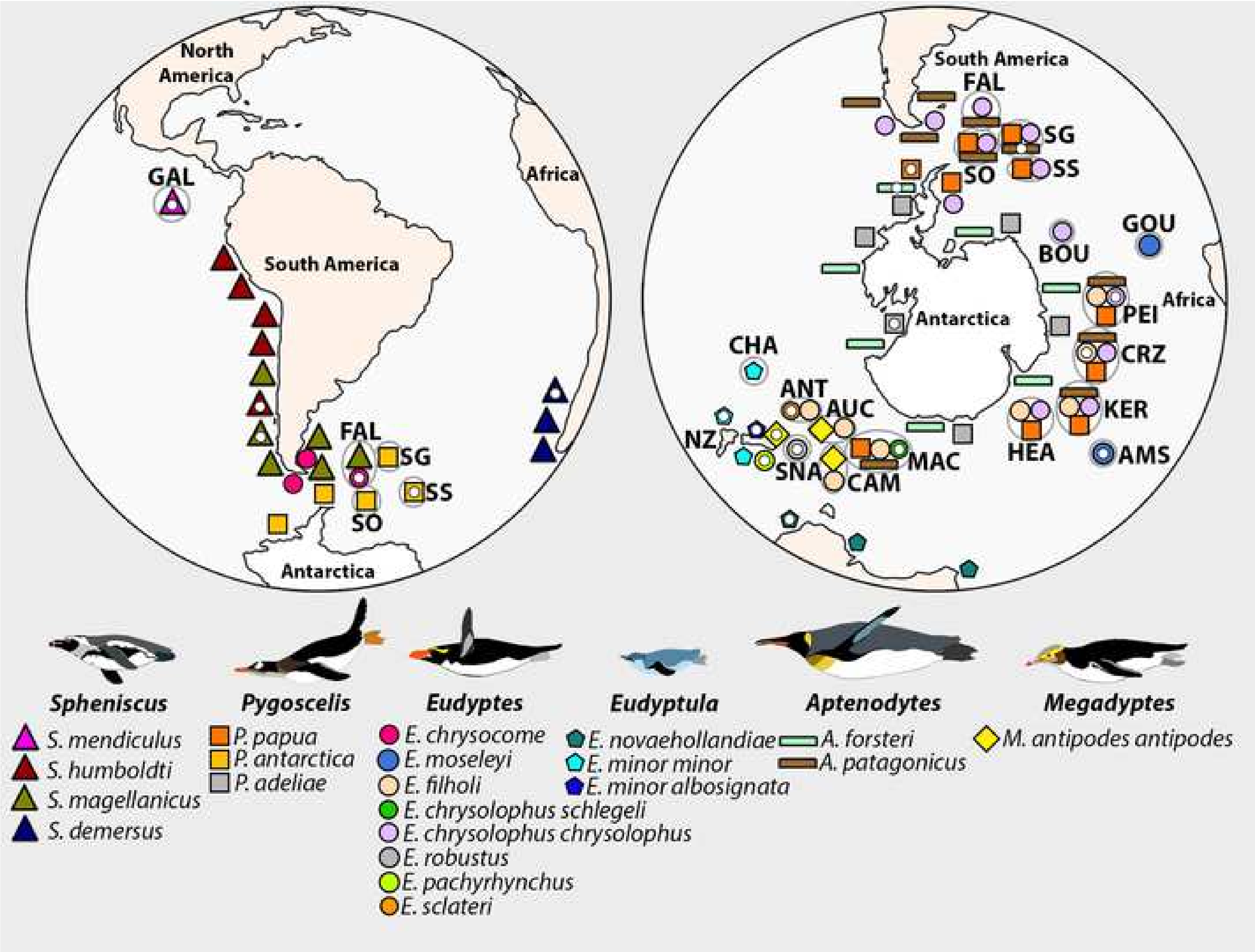

Figure 2 Click here to download Figure Figure 2.tif

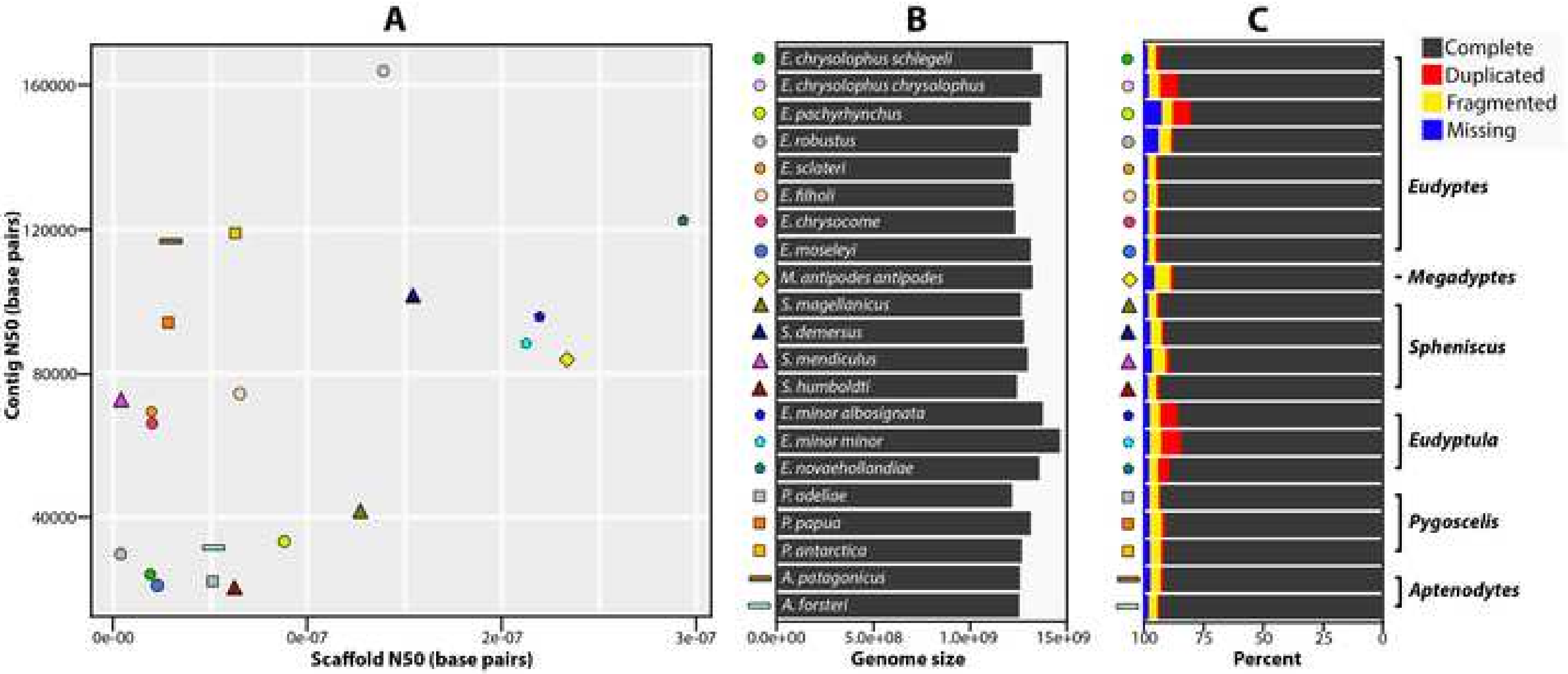

Cover Letter Click here to download Personal Cover COVER LETTER.pdf

DEPARTMENT OF BIOLOGY

UNIVERSITY OF COPENHAGEN

Cover letter to Penguin genome DATANOTE in GIGASCIENCE

3 AUGUST 2019

GUOJIE ZHANG,

PROFESSOR

DEPARTMENT OF BIOLOGY
UNIVERSITETSPARKEN 15
UNIVERSITY OF COPENHAGEN
DK-2100 COPENHAGEN
DENMARK

TEL +45 35 32 12 49
FAX +45 35 32 12 50

Guojie.Zhang@bio.ku.dk

http://bio.ku.dk/

Dear Editor,

Thanks for your interest of considering our manuscript entitled ***High-coverage genomes to elucidate the evolution of penguins*** for publication as a Data Note in *GigaScience*. We appreciate if you can arrange a fast track for peer review, so the community can access our genomic resources at the earliest.

Penguins are an exciting system for understanding the evolutionary processes of speciation, adaptation and demography. In our manuscript, we present a novel dataset of 19 high-coverage penguin genomes for early-release. These new genomes, together with two previously published penguin genomes, encompass all extant penguin species. In addition, we introduce a major consortium of international scientists dedicated to studying these genomes and highlight emerging issues on ensuring legal and respectful indigenous consultation for genomic data originating from New Zealand Taonga species. We believe that our dataset and project will be important for cultural heritage and the conservation of this iconic Southern Hemisphere species assemblage.

The conservation and evolution of the Southern Ocean's unique biodiversity has always been of great interest to the general public, and news about penguin biology generally excites considerable attention from the media. We have a strong track record of communicating key research findings through media releases, interviews and popular science articles – with previous findings highlighted by hundreds of news articles globally. Our submitted manuscript is expected to have a strong influence on understanding the evolution of penguins.

Thank you for considering our manuscript for publication in *GigaScience*. We look forward to hearing from you soon.

Yours sincerely,
Guojie Zhang, also on behalf of the consortium

Professor
Department of Biology, University of Copenhagen
Universitetsparken 15, 2100 Copenhagen, Denmark
Email guojie.zhang@bio.ku.dk Phone: +45 91855431
