# Supplementary material for: High-coverage genomes to elucidate the evolution of penguins
Source: Gigascience. 2019 Sep 18;8(9):giz117. doi: 10.1093/gigascience/giz117 (PMC6904868; doi:10.1093/gigascience/giz117)
Supplement: giz117_Supplemental_File [file giz117_supplemental_file.docx]

**Supplementary Information**

**Supplementary Figures**

**
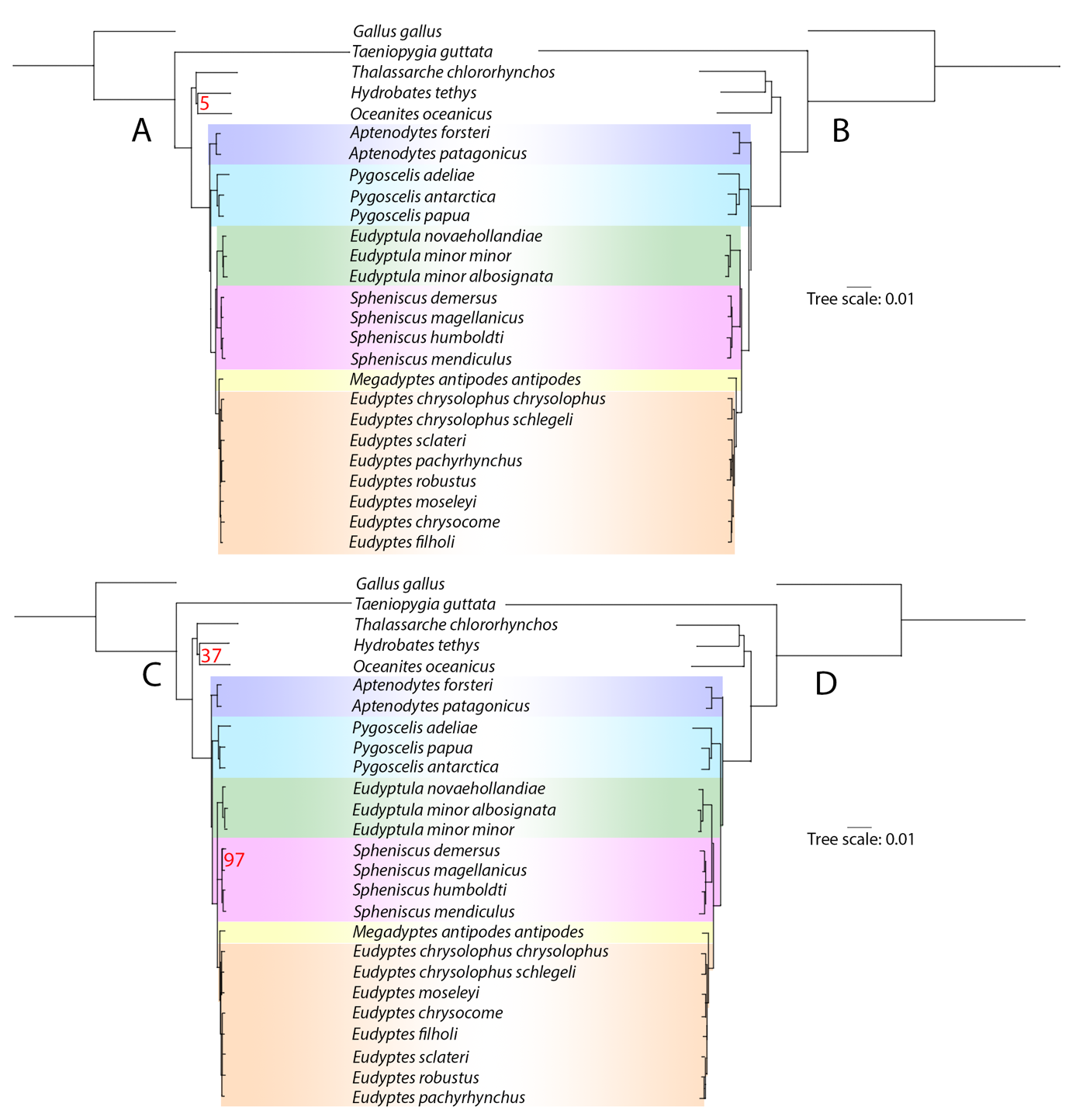
**

Supplementary Figure 1. Phylogenomic trees obtained using coalescent-based (A) MP-EST/ASTRAL compared with (B) concatenation-based ExaML “TrimAl data” and (C) the MP-EST/ASTRAL compared with (D) concatenation-based ExaML “No missing data”. Bootstrap support was 100 unless indicated in red.

**Supplementary Tables**

Supplementary Table 1. Sampling and permitting details of all penguin samples tested.

| **Latin Name** | **Sample Label** | **Sampling Location** | **Additional Samples Tested** | **Additional Sampling Locations** | **Animal Ethics** | **Permit Details** | **New Zealand consultation** |
| --- | --- | --- | --- | --- | --- | --- | --- |
| *Eudyptes chrysolophus schlegeli* | ROPE 4458 (Gary Miller) | Green Gorge, Macquarie Island | ROPE 4449 (Gary Miller), ROPE 4461 (Gary Miller) | Green Gorge, Macquarie Island | Minister for Primary Industries and Water, Tasmania, Licence to Conduct Research 58932 (Gary Miller) | Department of Primary Industries, Water and Environment permit to take wildlife FA 06378 (Gary Miller) | NA |
| *Eudyptes chrysolophus chrysolophus* | MP PEI 1 (Peter Ryan) | Marion Island, Prince Edward Islands | MP PEI 2 (Peter Ryan), MP PEI 3 (Peter Ryan), MP PEI 4 (Peter Ryan), MP PEI 5 (Peter Ryan), MP PEI 6 (Peter Ryan), MP PEI 7 (Peter Ryan) | Marion Island, Prince Edward Islands | UCT Science Faculty Animal Ethics Committee permit 2013/V5/PR (Peter Ryan) | Samples collected with permission from the South African Department of Environment Affairs (Peter Ryan) | NA |
| *Eudyptes pachyrhynchus* | MS 9 (Thomas Mattern/Theresa Cole) | Harrison Cove, Milford Sound, New Zealand South Island | MS 8 (Thomas Mattern/Theresa Cole), WH 01 2016 (Ursula Ellenberg/David Houston) | Harrison Cove, Milford Sound, New Zealand South Island; Codfish Island, Foveaux Strait, New Zealand | University of Otago Animal Ethics Committee 61/2016 (Jonathan Waters/Theresa Cole); Animal Ethics number AUP#38/14 (Thomas Mattern) | 50436-FAU (Theresa Cole), 54288-DOA (Theresa Cole), OT-25557-DOA (Bruce Robertson), RES-38882 (Thomas Mattern) | University of Otago Ngāi Tahu Research Consultation Committee (Jonathan Waters/Theresa Cole); Department of Conservation Māori Consultation (Theresa Cole); Department of Conservation Māori Consultation (Thomas Mattern) |
| *Eudyptes robustus* | 68M 28/09/13 (David Thompson) | The Snares | 38F 27/09/13 (David Thompson), 48 gvn 28/09/13 (David Thompson) | The Snares | University of Otago Animal Ethics Committee 61/2016 (Jonathan Waters/Theresa Cole); NIWA Animal Ethics Committee Approvals (David Thompson) | 62361-FAU (Lisa Argilla), 50436-FAU (Theresa Cole), 50437-DOA (Theresa Cole), 35682-FAU (David Thompson), OT-25557-DOA (Bruce Robertson) | University of Otago Ngāi Tahu Research Consultation Committee (Jonathan Waters/Theresa Cole); Department of Conservation Māori Consultation (Theresa Cole); Department of Conservation Māori Consultation (David Thompson) |
| *Eudyptes sclateri* | Ant 5 (Chris Rickard/Sarah Fraser) | Antipodes Island, New Zealand sub-Antarctic | Ant 48 (Chris Rickard/Sarah Fraser), Ant 11 (Chris Rickard/Sarah Fraser), 180236 Purakaunui Bay 11/3/18 (Lisa Argilla) | Antipodes Island, New Zealand sub-Antarctic; Purakaunui Bay, New Zealand South Island | National Institute of Water and Atmospheric Research ethics approval (David Thompson); University of Otago Animal Ethics Committee 61/2016 (Jonathan Waters/Theresa Cole) | SO-28366-FAU (Kath Walker/Graeme Elliott/Chris Rickard/Sarah Fraser/David Thompson); 62361-FAU (Lisa Argilla), 50436-FAU (Theresa Cole), 50464-DOA (Theresa Cole), OT-25557-DOA (Bruce Robertson) | University of Otago Ngāi Tahu Research Consultation Committee (Jonathan Waters/Theresa Cole); Department of Conservation Māori Consultation (Theresa Cole) |
| *Eudyptes filholi* | GS 12 (Yves Cherel) | Possession Island, Crozet Islands | NA | NA | Fieldwork was approved by the Ethic Committee of the French Polar Institute (IPEV) (Yves Cherel) | The permit (no number) was delivered to the IPEV program no. 109 (H. Weimerskirch) | NA |
| *Eudyptes chrysocome* | RH 110 (Dee Boersma) | Falkland Islands/Malvinas | NA | NA | IACUC eGC1 # A145289 (Dee Boersma) | MBTA Permit #: MB098566 (Robert Faucett); Sample collected with permission from the Falkland Islands Government (Amy van Buren/Dee Boersma) and the Burke Museum at the University of Washington (Amy van Buren/Dee Boersma) | NA |
| *Eudyptes moseleyi* | NRP 118 (Yves Cherel) | Amsterdam Island | NRP 115 (Yves Cherel) | Amsterdam Island | Fieldwork was approved by the Ethic Committee of the French Polar Institute (IPEV) (Yves Cherel) | The permit (no number) was delivered to the IPEV program no. 109 (H. Weimerskirch) | NA |
| *Megadyptes antipodes antipodes* | OT 2 9/2/18 (Melanie Young) | Otago Peninsula, New Zealand South Island | OT 3 9/2/18 (Melanie Young), OT 4 9/2/18 (Melanie Young), OT 5 9/2/18 (Melanie Young), OT 6 9/2/18 (Melanie Young) | Otago Peninsula, New Zealand South Island | University of Otago Animal Ethics Committee 48/2016 (Bruce Robertson/Melanie Young) | 50925-FAU (Melanie Young) | Department of Conservation Māori Consultation (Theresa Cole) |
| *Spheniscus magellanicus* | AH 6 (Juan Bouzat | Chiloé Island, Chile | PA1 (Juan Bouzat), PA2 (Juan Bouzat), PA3 (Juan Bouzat), PA4 (Juan Bouzat), AH3 (Juan Bouzat), AH4 (Juan Bouzat), AH5 (Juan Bouzat), S1 (Juan Bouzat), S2 (Juan Bouzat), S3 (Juan Bouzat),  S4 (Juan Bouzat),  E1 (Dee Boersma/Juan Bouzat), E2 (Dee Boersma/Juan Bouzat), E3 (Dee Boersma/Juan Bouzat), E4 Dee Boersma/Juan Bouzat)  JAC29 (Dee Boersma/Juan Bouzat), JAC34 (Dee Boersma/Juan Bouzat), JAC74 (Dee Boersma/Juan Bouzat), JAC76 (Dee Boersma/Juan Bouzat), H1 (Dee Boersma/Juan Bouzat), H2 (Dee Boersma/Juan Bouzat), H3 (Dee Boersma/Juan Bouzat), H20 (Dee Boersma/Juan Bouzat), M1 (Dee Boersma/Juan Bouzat), M2 (Dee Boersma/Juan Bouzat), M3 (Dee Boersma/Juan Bouzat), M4 (Dee Boersma/Juan Bouzat) | Atlantic and Pacific, various locations | Division of Fisheries of the National Government of Chile and the Forestry National Division (CONAF) Permit # 3523 (Juan Bouzat)  University of Washington IACUC (Dee Boersma) | Division of Fauna and Flora of the Government of Argentina Permit # 18/02FyFS; USDA-APHIS Importation Permit # 42579 (Robert Faucett/Dee Boersma; Burke Museum at the University of Washington) | NA |
| *Spheniscus demersus* | AP 173 (Kim Labuschagne) | Luderitz, Namibia | NUP 1 (Lisa Nupen), NUP 39 (Lisa Nupen), NUP 77 (Lisa Nupen), AP 127 (Kim Labuschagne) | St Croix Island, Eastern Cape Coast, South Africa; Dassen Island, South Western coast South Africa; Mercury Island, Namibia; Luderitz, Namibia | University of Cape Town Science Faculty Animal Ethics clearance number 2009/V21/LN (Lisa Nupen); SANCCOB (Kim Labuschagne) | the South African Department of Environmental Affairs permit number RES2010/66 (Lisa Nupen); CapeNature collection permit number AAA-004-00520-0035 (Lisa Nupen); South African National Parks approval (Lisa Nupen); the Namibian Ministry of Environment, Natural Resources and Transport: Wildlife Enforcement and Permits Division, Wildlife Trade and Conservation Section (Lisa Nupen); CITES permit 204790 (Lisa Nupen); NZG/RES/P17/42; the South African Department of Environmental Affairs Standing permit number S07901 (Kim Labuschagne), National Zoological Garden, South African National Biodiversity Institute NZG/RES/P17/42 (Kim Labuschagne) CITES Permit/Certificate No. 207882 (Kim Labuschagne) | NA |
| *Spheniscus mendiculus* | GAPE 212 (Patricia Parker) | Galápagos Islands | GAPE PR 21 (Patricia Parker), GAPE 458 (Patricia Parker), GAPE PR 2039 (Patricia Parker) | Galápagos Islands | University of Missouri – St. Louis IACUC Permit (Patricia Parker) | United States Veterinary Permit for Importation and Transport of Controlled Materials and Organisms and Vectors Permit Number: 47418 (Patricia Parker); U.S. Fish and Wildlife Service Declaration for Importation or Exportation of fish or wildlife O.M.B. No. 1018-0012 (Patricia Parker); Department of the Interior U.S. Fish and Wildlife Service, Federal Fish and Wildlife Permit Number: MA42831A-0 (Jeffrey Brown/Dana Brown/Patricia Parker); Convenio de Cooperacion Interinstitucional Letter (Patricia Parker) | NA |
| *Spheniscus humboldti* | Z-67-15 (Mads Bertelsen) | Peru and Chile lineage | NA | NA | Z-67-15 2016-10, Collection approval from Copenhagen Zoo internal scientific committee (IACUC) (Mads Bertelsen) | Z-67-15 2016-10, Collection approval from Copenhagen Zoo internal scientific committee (IACUC) (Mads Bertelsen) | NA |
| *Eudyptula minor albosignata* | Fred (Pauline Howard) | Banks Peninsula, Canterbury, New Zealand South Island | Dane (Pauline Howard), BP 06 (Stefanie Grosser), BP 17 (Stefanie Grosser) | Christchurch Antarctic Centre, originally Banks Peninsula, New Zealand South Island; Otanerito Bay, Banks Peninsula, New Zealand South Island | University of Otago Animal Ethics Committee 59/2010 (Yolanda van Heezik/Scott Flemming) | 50436-FAU (Theresa Cole), 54288-DOA (Theresa Cole), 39997-FAU (Thomas Stracke/Kristina Schutt), CA-32742-CAP (International Antarctic Centre Ltd), CA-28817-FAU (Scott Flemming) | University of Otago Ngāi Tahu Research Consultation Committee (Yolanda van Heezik/Scott Flemming); Department of Conservation Māori Consultation (Theresa Cole) |
| *Eudyptula minor minor* | Gonzo (Helen Taylor) | New Zealand North Island | Bop 48 (Stefanie Grosser), LBP Waitarere 13/4/18 (Emily Kay), Peppa (Helen Taylor), Lulle (Helen Taylor), Dora F (Helen Taylor), Elmo F (Helen Taylor), Draco (Helen Taylor) | Bay of Plenty, New Zealand North Island; Waitarere, New Zealand North Island; National Aquarium of New Zealand, originally New Zealand North Island | Animal Ethics as referred to in (3), Stefanie Grosser; Sample collected for clinical purposes by Wildbase Hospital within the Massey University Veterinary Teaching Hospital (Emily Kay) | OT-34124-DOA (Stefanie Grosser), OT-25557-DOA (Bruce Robertson), 36555-FAU (Wildbase Hospital); 50436-FAU (Theresa Cole), 54288-DOA (Theresa Cole), 40460-CAP (National Aquarium of New Zealand) | Department of Conservation Māori Consultation (Theresa Cole) |
| *Eudyptula novaehollandiae* | 10/9/18-1 (Peter Dann) | Phillip Island, Victoria, Australia | Oa 201 (Stefanie Grosser), Oa 93 (Stefanie Grosser), 10/9/18-2 (Peter Dann) | Otago, New Zealand South Island; Phillip Island, Victoria, Australia | Animal Ethics as referred to in (3); Phillip Island Nature Park Animal Ethics Committee 3.2018 (Peter Dann) | OT 34124-DOA (Stefanie Grosser), OT-25557-DOA (Bruce Robertson), 50436-FAU (Theresa Cole), 54288-DOA (Theresa Cole), DELWP 10008780 (Peter Dann) | Department of Conservation Māori Consultation (Theresa Cole) |
| *Pygoscelis papua* | Gentoo penguin DNA -4 | West Antarctic Peninsula, Antarctica | GP MOOT 011 (Tom Hart), GP OH 101 (Tom Hart), GP 006 (Tom Hart) | West Antarctic Peninsula, Antarctica | Oxford University Animal Ethics committee (Tom Hart) | FCO 34/2016 (Tom Hart) | NA |
| *Pygoscelis antarctica* | CP TH 060 (Tom Hart) | Thule Island, South Sandwich Islands | CP TH 058 (Tom Hart) | Thule Island, South Sandwich Islands | ZSL animal ethics committee (Tom Hart), Oxford Animal Ethics Committee (Tom Hart) | Letter of permit from GSGSSI no number (Tom Hart); David Lambert Permit | NA |
| *Aptenodytes patagonicus* | KP FORT 001 (Tom Hart) | Fortuna Bay, South Georgia | KP FORT 002 (Tom Hart), KP FORT 003 (Tom Hart), KP FORT 010 (Tom Hart), King penguin DNA (Tom Hart/Steven Fiddaman) | Fortuna Bay, South Georgia | ZSL animal ethics committee (Tom Hart); Oxford Animal Ethics Committee (Tom Hart) | Letter of permit from GSGSSI no number (Tom Hart) | NA |

Supplementary Table 2. Assemblers and Kmer sizes used for each penguin. The Kmer sizes were only for species assembled by SOAPdenovo2. Kmer sizes of *Pygoscelis adeliae* and *Aptenodytes forsteri* were from (2).

| **Species** | **Assembler** | **Kmer size (bp)** |
| --- | --- | --- |
| *Eudyptes chrysolophus schlegeli* | SOAPdenovo2-2.04 | 25 |
| *Eudyptes chrysolophus chrysolophus* | Supernova-2.0.0 | NA |
| *Eudyptes pachyrhynchus* | SOAPdenovo2-2.04 | 35 |
| *Eudyptes robustus* | SOAPdenovo2-2.04 | 35 |
| *Eudyptes sclateri* | Allpaths-lg | NA |
| *Eudyptes filholi* | Allpaths-lg | NA |
| *Eudyptes chrysocome* | Allpaths-lg | NA |
| *Eudyptes moseleyi* | SOAPdenovo2-2.04 | 35 |
| *Megadyptes antipodes antipodes* | Supernova-2.0.0 | NA |
| *Spheniscus magellanicus* | SOAPdenovo2-2.04 | 25 |
| *Spheniscus demersus* | Supernova-2.0.0 | NA |
| *Spheniscus mendiculus* | Supernova-2.0.0 | NA |
| *Spheniscus humboldti* | SOAPdenovo2-2.04 | 43 |
| *Eudyptula minor albosignata* | Supernova-2.0.0 | NA |
| *Eudyptula minor minor* | Supernova-2.0.0 | NA |
| *Eudyptula novaehollandiae* | Supernova-2.0.0 | NA |
| *Pygoscelis adeliae* | SOAPdenovo2 | 19 |
| *Pygoscelis papua* | Supernova-2.0.0 | NA |
| *Pygoscelis antarctica* | Supernova-2.0.0 | NA |
| *Aptenodytes patagonicus* | Supernova-2.0.0 | NA |
| *Aptenodytes forsteri* | SOAPdenovo2 | 19 |

Supplementary Table 3. Information of 71 avian transcriptomic samples downloaded from NCBI.

| **Organism Name** | **Order** | **Family** | **Number of Samples** | **Date Publicly Released** | **SRP ID** | **Sequencing Platform** |
| --- | --- | --- | --- | --- | --- | --- |
| *Anas platyrhynchos* | Anseriformes | Anatidae | 2 | 4-Feb-13 | SRP018391 | Illumina HiSeq 2000 |
| *Aegypius monachus* | Falconiformes | Accipitridae | 1 | 29-Apr-14 | SRP041562 | Illumina HiSeq 2500 |
| *Alectoris rufa* | Galliformes | Phasianidae | 8 | 26-Nov-14 | SRP050314 | Illumina Genome Analyzer IIx, SE |
| *Anas platyrhynchos* | Anseriformes | Anatidae | 4 | 27-Mar-13 | SRP020086 | Illumina HiSeq 2000 |
| *Anser cygnoides* | Anseriformes | Anatidae | 1 | 15-Jan-14 | SRP035437 | Illumina HiSeq 2000, SE |
| *Anser sp.(goose)* | Anseriformes | Anatidae | 1 | 26-Dec-13 | SRP034727 | Illumina HiSeq 2000 |
| *Apteryx australis mantelli* | Apterygiformes | Apterygidae | 1 | 20-Dec-11 | SRP003481 | 454 GS FLX, SE |
| *Carduelis chloris* | Passeriformes | Fringillidae | 2 | 1-Apr-14 | SRP040761 | Illumina HiSeq 2500 |
| *Corvus macrorhynchos* | Passeriformes | Corvidae | 1 | 29-Oct-13 | SRP032433 | 454 GS FLX Titanium, SE |
| *Coturnix japonica* | Galliformes | Phasianidae | 1 | 6-May-14 | SRP043003 | 454 GS FLX, SE |
| *Coturnix japonica* | Galliformes | Phasianidae | 2 | 24-Sep-15 | SRP066885 | Illumina HiSeq 2000 |
| *Cyanopica cyana* | Passeriformes | Corvidae | 1 | 16-Mar-15 | SRP056221 | Illumina HiSeq 2000, SE |
| *Dromaius novaehollandiae* | Casuariiformes | Dromaiidae | 1 | 18-Mar-13 | SRP019802 | Illumina HiSeq 2000 |
| *Gallinago media* | Charadriiformes | Scolopacidae | 14 | 30-Oct-12 | SRP016881 | 454 GS FLX Titanium, SE |
| *Haemorhous mexicanus* | Passeriformes | Fringillidae | 2 | 6-Mar-13 | SRP018959 | Illumina HiSeq 2000 |
| *Junco hyemalis* | Passeriformes | Passerellidae | 2 | 28-Jul-14 | SRP052228 | Illumina HiSeq 2000, SE |
| *Junco hyemalis* | Passeriformes | Passerellidae | 1 | 13-Apr-12 | SRP012466 | 454 GS FLX Titanium, SE |
| *Lamprotornis superbus* | Passeriformes | Sturnidae | 2 | 4-Sep-14 | SRP046157 | Illumina HiSeq 2000, SE |
| *Meleagris gallopavo* | Galliformes | Phasianidae | 2 | 3-Jun-14 | SRP042724 | Illumina Genome Analyzer IIx, SE |
| *Melospiza melodia* | Passeriformes | Passerellidae | 2 | 17-Mar-14 | SRP040239 | Illumina HiSeq 2000 |
| *Numida meleagris* | Galliformes | Numididae | 3 | 6-Jun-12 | SRP021481 | Illumina HiSeq 2000, SE |
| *Paradoxornis webbianus bulomachus (Sinosuthora webbiana)* | Passeriformes | Sylviidae | 1 | 1-Sep-11 | SRP007844 | Illumina HiSeq 2000, SE |
| *Parus major* | Passeriformes | Paridae | 8 | 20-Dec-11 | SRP004680 | 454 GS FLX Titanium, SE |
| *Phylloscopus trochilus(acredula)* | Passeriformes | Phylloscopidae | 2 | 25-Jul-12 | SRP014608 | 454 GS FLX Titanium, SE |
| *Pseudopodoces humilis* | Passeriformes | Paridae | 2 | 1-Mar-13 | SRP018927 | Illumina HiSeq 2000 |
| *Uraeginthus granatina* | Passeriformes | Estrildidae | 1 | 22-Aug-13 | SRP029159 | 454 GS FLX, SE |
| *Zonotrichia albicollis* | Passeriformes | Emberizidae | 2 | 30-Aug-13 | SRP029385 | Illumina HiSeq 2000, SE |
| *Zonotrichia leucophrys gambelii* | Passeriformes | Emberizidae | 1 | 10-Apr-14 | SRP041107 | Illumina HiSeq 2500 |

**Supplementary References**

1. Cole TL, Ksepka DT, Mitchell KJ, et al. Mitogenomes uncover extinct penguin taxa and reveal island formation as a key driver of speciation. Mol Biol Evol. 2019; 36(4):784–797.

2. Li C, Zhang Y, Li J, et al. Two Antarctic penguin genomes reveal insights into their evolutionary history and molecular changes related to the Antarctic environment. GigaScience. 2014; 3(1):27.

3. Grosser S. Molecular systematics and phylogeography of little penguins. Ph.D. Thesis. The University of Otago. 2015.
