# Supplementary material for: High-coverage genomes to elucidate the evolution of penguins
Source: Gigascience. 2019 Sep 18;8(9):giz117. doi: 10.1093/gigascience/giz117 (PMC6904868; doi:10.1093/gigascience/giz117)
Supplement: giz117_Response_to_Reviewer_Comments_Original_Submission [file giz117_response_to_reviewer_comments_original_submission.pdf]

Author's Response To Reviewer Comments

Close

Your manuscript "High-coverage genomes to elucidate the evolution of penguins" (GIGA-D-19-00280) has been assessed by our reviewers. Based on these reports, and my own assessment as Editor, I am pleased to inform you that it is potentially acceptable for publication in GigaScience, once you have carried out some minor revisions suggested by our reviewers.
Thank you for giving us the opportunity to improve our manuscript. We hope this revised version is acceptable for publication in GigaScience.

Reviewer 1

The manuscript "High-coverage genomes to elucidate the evolution of penguins" by Pan et al reports a high-coverage genome assembly for the 19 penguins. Penguins are very interesting birds as they are the unique physiological and morphological adaptations species including interesting adaptations to the extreme environment they are living in. This study could provide massive information to understanding all clade of penguins and also birds biology. From my point of view, I suggest two things are added.
Thank you for your positive review. We have now incorporated your two suggestions which have improved the manuscript.

First one is assembly statistics make the table and might be provided.
Done. We have now added a table (Table 3) with the assembly statistics.

Second is phylogenetic tree of all penguins should be provided which can explain how penguins are evolving.
Done. We have now added a phylogenetic tree (Figure 3), including the methods.

Overall this is an exciting paper, despite the lack of further functional validations.
Thank you. This is a big project that we believe will provide some exciting insights into functional adaptation and evolution.

Reviewer 2

This data note paper introduces a new consortium of dedicated scientists who presents a high-coverage dataset of 19 penguin genomes completes the coverage all living penguin species in the world, which will allow further studies in comparative evolutionary history of birds. Penguins evolutionary history is interesting not only because of their novel adaptation to the aquatic lifestyle, but also an excellent case study for researching speciation from the biogeographic perspective. However, while penguins represent a relatively well-studied and extremely charismatic group, ecological and evolutionary studies in these species have previously been limited by the availability of genetic markers. I am very excited about the publication of this new and important data resource.
Thank you. We believe it will be an important contribution to the scientific community.

The paper is written in a clear and simple language and represents a straightforward and standard genome assembly and annotation approach that has been employed for the bird genomes in the past. However, there are some small issues that I wish had been made clear before the publication of this data. Here I list four of them:
Thank you for your constructive suggestions for improving our manuscript. We have amended/clarified our manuscript, and hope that it is satisfactory.

There are several technologies mixed in this data note, as well as several assembly approaches. It was not easy to figure out which genomes were done with the 10X after the statement "We constructed 10X genomic libraries for each species with DNA fragments longer than 40 Kbp." (line #210). Also in in this sense, it needs to be clarified what are the quality impacts of each assembly. Especially valuable would be the contrast between the Illumina and the BGISeq.

Thank you for your comments and for letting us know that this was confusing. We have included the library information in table 2 which shows the sequencing and assembly strategy for each species. While we did not observe a difference on assembly quality between sequencing platforms, we did observe the 10X strategy normally produces better assembly than the multi-libraries strategy. We have indicated these two categories in table 3.

Table 2. Since for the for the Supernova assemblies usually there is no need for pre-filtration, it is look a bit strange that in Table 2 most of the data 25-30% re filtered out. Can you explain what was the purpose for this procedure, and when it was done?
The filtered reads for the 10X libraries were only used for estimating the genome size with 17 k-mer, while all reads were used for Supernova assembly. We have clarified this in the text.

Please clarify Figure 2(A) - is difficult to understand in the current format. Perhaps a table can accompany it so numbers are easier to read.
We have included the assembly statistics in Table 3. In Figure 2A, Each point (represented by triangles, squares, circles, rectangles and pentagon symbols) indicates a penguin species, the x-axis indicates the scaffold N50 and the y-axis indicates the contig N50 for each species. We have clarified this in the title.

I would recommend to add the newest EggNOG 5.0 functional annotation (Huerta-Cepas et al., 2019), it would be easy to do and would contribute to the possibility of cross-referencing of this dataset.
We have used the annotation pipeline developed by the B10K consortium specifically for bird genomes. The same pipeline has also been used in all other bird genomes produced by B10K. So far, over 400 bird genomes have been annotated in the same method, which we believe is crucial for the entire community as only by doing this all data can be comparable. Therefore, while we agree that it might be a good idea to use different annotation pipelines for selecting the best gene model, as a consortium, we have to prioritize on generating a standard annotation that can be widely used for most researchers.

Close
