# Supplementary material for: High-coverage genomes to elucidate the evolution of penguins
Source: Gigascience. 2019 Sep 18;8(9):giz117. doi: 10.1093/gigascience/giz117 (PMC6904868; doi:10.1093/gigascience/giz117)
Supplement: giz117_Reviewer_2_Report_Original_Submission — Taras K Oleksyk, Ph.D. -- 8/26/2019 Reviewed [file giz117_reviewer_2_report_original_submission.pdf]

**Reviewer Report**

**Title: High-coverage genomes to elucidate the evolution of penguins**

**Version: Original Submission** **Date:** 8/26/2019

**Reviewer name: Taras K Oleksyk, Ph.D.**

**Reviewer Comments to Author:**

This data note paper introduces a new consortium of dedicated scientists who presents a high-coverage dataset of 19 penguin genomes completes the coverage all living penguin species in the world, which will allow further studies in comparative evolutionary history of birds.  Penguins evolutionary history is interesting not only because of their novel adaptation to the aquatic lifestyle, but also an excellent case study for researching speciation from the biogeographic perspective.  However, while penguins represent a relatively well-studied and extremely charismatic group, ecological and evolutionary studies in these species have previously been limited by the availability of genetic markers.  I am very excited about the publication of this new and important data resource.
The paper is written in a clear and simple language and represents a straightforward and standard genome assembly and annotation approach that has been employed for the bird genomes in the past.  However, there are some small issues that I wish had been made clear before the publication of this data.  Here I list four of them:
1. There are several technologies mixed in this data note, as well as several assembly approaches.  It was not easy to figure out which genomes were done with the 10X after the statement "We constructed 10X genomic libraries for each species with DNA fragments longer than 40 Kbp." (line #210).  Also in in this sense, it needs to be clarified what are the quality impacts of each assembly.  Especially valuable would be the contrast between the Illumina and the BGISeq.  
2. Table 2.  Since for the for the Supernova assemblies usually there is no need for pre-filtration, it is look a bit strange that in Table 2 most of the data 25-30% re filtered out.  Can you explain what was the purpose for this procedure, and when it was done?
3. Please clarify Figure 2(A) - is difficult to understand in the current format.  Perhaps a table can accompany it so numbers are easier to read.  
4. I would recommend to add the newest EggNOG 5.0 functional annotation (Huerta-Cepas et al., 2019), it would be easy to do and would contribute to the possibility of cross-referencing of this dataset.

**Level of Interest**

Please indicate how interesting you found the manuscript: Choose an item.

**Quality of Written English**

Please indicate the quality of language in the manuscript: Choose an item.

**Declaration of Competing Interests**

Please complete a declaration of competing interests, considering the following questions:

- Have you in the past five years received reimbursements, fees, funding, or salary from an organisation that may in any way gain or lose financially from the publication of this manuscript, either now or in the future?
- Do you hold any stocks or shares in an organisation that may in any way gain or lose financially from the publication of this manuscript, either now or in the future?
- Do you hold or are you currently applying for any patents relating to the content of the manuscript?
- Have you received reimbursements, fees, funding, or salary from an organization that holds or has applied for patents relating to the content of the manuscript?
- Do you have any other financial competing interests?
- Do you have any non-financial competing interests in relation to this paper?

If you can answer no to all of the above, write 'I declare that I have no competing interests' below. If your reply is yes to any, please give details below.

I declare that I have no competing interest

I agree to the open peer review policy of the journal. I understand that my name will be included on my report to the authors and, if the manuscript is accepted for publication, my named report including any attachments I upload will be posted on the website along with the authors' responses. I agree for my report to be made available under an Open Access Creative Commons CC-BY license (http://creativecommons.org/licenses/by/4.0/). I understand that any comments which I do not wish to be included in my named report can be included as confidential comments to the editors, which will not be published.

Choose an item.

To further support our reviewers, we have joined with Publons, where you can gain additional credit to further highlight your hard work (see: https://publons.com/journal/530/gigascience). On publication of this paper, your review will be automatically added to Publons, you can then choose whether or not to claim your Publons credit. I understand this statement.

Yes Choose an item.
